# Gene signature associated with benign neurofibroma transformation to malignant peripheral nerve sheath tumors

**DOI:** 10.1371/journal.pone.0178316

**Published:** 2017-05-24

**Authors:** Marta Martínez, Carlos O. S. Sorzano, Alberto Pascual-Montano, Jose M. Carazo

**Affiliations:** 1 Biocomputing Unit, Nacional Center for Biotechnology (CSIC), Campus Universidad Autónoma de Madrid, Cantoblanco, Madrid, Spain; 2 Bioengineering Lab., Universidad CEU San Pablo, Campus Urb. Montepríncipe, Boadilla del Monte, Madrid, Spain; Universidad de Navarra, SPAIN

## Abstract

Benign neurofibromas, the main phenotypic manifestations of the rare neurological disorder neurofibromatosis type 1, degenerate to malignant tumors associated to poor prognosis in about 10% of patients. Despite efforts in the field of (epi)genomics, the lack of prognostic biomarkers with which to predict disease evolution frustrates the adoption of appropriate early therapeutic measures. To identify potential biomarkers of malignant neurofibroma transformation, we integrated four human experimental studies and one for mouse, using a gene score-based meta-analysis method, from which we obtained a score-ranked signature of 579 genes. Genes with the highest absolute scores were classified as promising disease biomarkers. By grouping genes with similar neurofibromatosis-related profiles, we derived panels of potential biomarkers. The addition of promoter methylation data to gene profiles indicated a panel of genes probably silenced by hypermethylation. To identify possible therapeutic treatments, we used the gene signature to query drug expression databases. Trichostatin A and other histone deacetylase inhibitors, as well as cantharidin and tamoxifen, were retrieved as putative therapeutic means to reverse the aberrant regulation that drives to malignant cell proliferation and metastasis. This *in silico* prediction corroborated reported experimental results that suggested the inclusion of these compounds in clinical trials. This experimental validation supported the suitability of the meta-analysis method used to integrate several sources of public genomic information, and the reliability of the gene signature associated to the malignant evolution of neurofibromas to generate working hypotheses for prognostic and drug-responsive biomarkers or therapeutic measures, thus showing the potential of this *in silico* approach for biomarker discovery.

## Introduction

Neurofibromatosis type 1 disease (NF1; Online Mendelian Inheritance in Man/OMIM—database #162200) is a rare chronic neurological disorder caused by a deficient autosomal dominant genetic background, which affects 1 in 3000 live births [1]. Alterations in the tumor suppressor gene neurofibromin *(NF1)* enhance expression of the Ras signaling pathway, which is involved in the evolution of many cancers [2]. Patients develop various anomalies in skin, eyes and skeleton, as well as in the cardiovascular, endocrine and nervous systems. In the peripheral nervous system, disorders typically manifest as benign neurofibromas (NF). Dermal neurofibromas (dNF) arise from small cutaneous nerves, whereas plexiform neurofibromas (pNF) have a deeper location within larger nerves; dNF and pNF gene expression patterns are indistinguishable [3]. In ~10% of NF1 patients, pNF can degenerate to malignant peripheral nerve sheath tumors (MPNST). About 50% of MPNST cases associate to NF1 disease, whereas the other 50% appear sporadically. Whether there are significant biological differences between sporadic and NF1-associated MPNST cases is debated [4–7].

The likelihood of MPNST development in NF1 patients depends on several risk factors [8], and appropriate prediction of pNF evolution would help to stratify patients and to choose the best early treatment. Despite recent advances based on studies of concomitant alterations in genes other than *NF1*, gene copy number alteration, epigenetic changes and gene expression, no prognostic biomarkers are available. Unlike other types of sarcoma, MPNST show a wide spectrum of chromosomal alterations [9]. In both sporadic and NF1-associated MPNST, amplifications are more frequent than deletions and affect the distal part of chromosome arm 17q [10]. Deletions mainly involve band p21 of chromosome 9, thus driving a dose reduction of the kinase inhibitor CDKN2A [11], and the proximal part of 17q, where *NF1* appears to be co-deleted with *SUZ12*, which encodes a member of the epigenetic regulator polycomb repressor complex 2 (PRC2). Genetic modifications due to PRC2 silencing are suggested as biomarkers for NF1 patient stratification [12].

Hypermethylation of tumor suppressor genes and hypomethylation of oncogenes are epigenetic changes reported for most cancers. A whole-methylome analysis identified 3690 genes probably associated with MPNST development and progression; among these were genes that encode CDKN2A and the tumor suppressors SOX10 and RASSF1, a Ras association domain family member [13]. *RASSF1A* silencing by promoter methylation is a biomarker of NF1-associated MPNST patients with poor prognosis [14].

Genome-wide RNA expression studies encompass patient samples [3,6,10,15,16], human cell cultures including NF- and MPNST-derived cell lines [1,3], and mouse models [17] that replicate human NF histology [18]. Gene expression profiles serve to identify disease biomarkers, such as *BIRC5*, *TOP2A* and *TK1*, that categorize MPNST patients with poor prognosis after surgery [10], or might also help to identify new therapeutic agents through drug repositioning, *i*.*e*., use of tested drugs to treat new disease indications [19]. NFFinder and other bioinformatics tools compare gene signatures to seek potential repurposing medicines in the context of orphan diseases [20]. Although a unique average gene signature associated to changes from NF to MPNST would be desirable as input for NFFinder, differences in sample nature, array platforms, and hybridization protocols hamper direct comparison of results among studies, which explains the current lack of attention to genomic data integration in neurofibromatosis.

The combination of data from public databases such as the Gene Expression Omnibus (GEO) and ArrayExpress [21,22] and development of high-throughput technologies has led generalization of data integration by meta-analysis in genomic research. By using statistical tools to combine independent studies, microarray meta-analysis approaches extract consistent average gene expression signatures as well as interaction networks [23]. Robust, unsupervised meta-analysis approaches are currently being developed based on projections of high-dimensional data into a low-dimensional space to infer dependency among data [24]. These approaches nonetheless require a relatively large sample number [25] and connections among the different -omics data, which precludes their use in studies for which information is limited. Combining pre-calculated values (P-values and effect sizes) led to a useful method to integrate heterogeneous data, qualitative and quantitative, for analysis of diabetes mellitus and Down syndrome [23,26]. This method, based on adding a score determined for each gene in each experiment using a formula similar to that applied in correlation studies, weights the size of the effect with reproducibility for all sample replicates and the statistical significance of the differentially expressed genes. As a starting point to identify biomarkers in the context of neurofibromatosis, we used a slight modification of this formula to integrate public genomic data and obtained a robust ranked score gene signature associated to the NF transition to MPNST.

## Results

### Experimental sets used to define the gene expression signature associated to NF-to-MPNST transition

**Fig 1:**
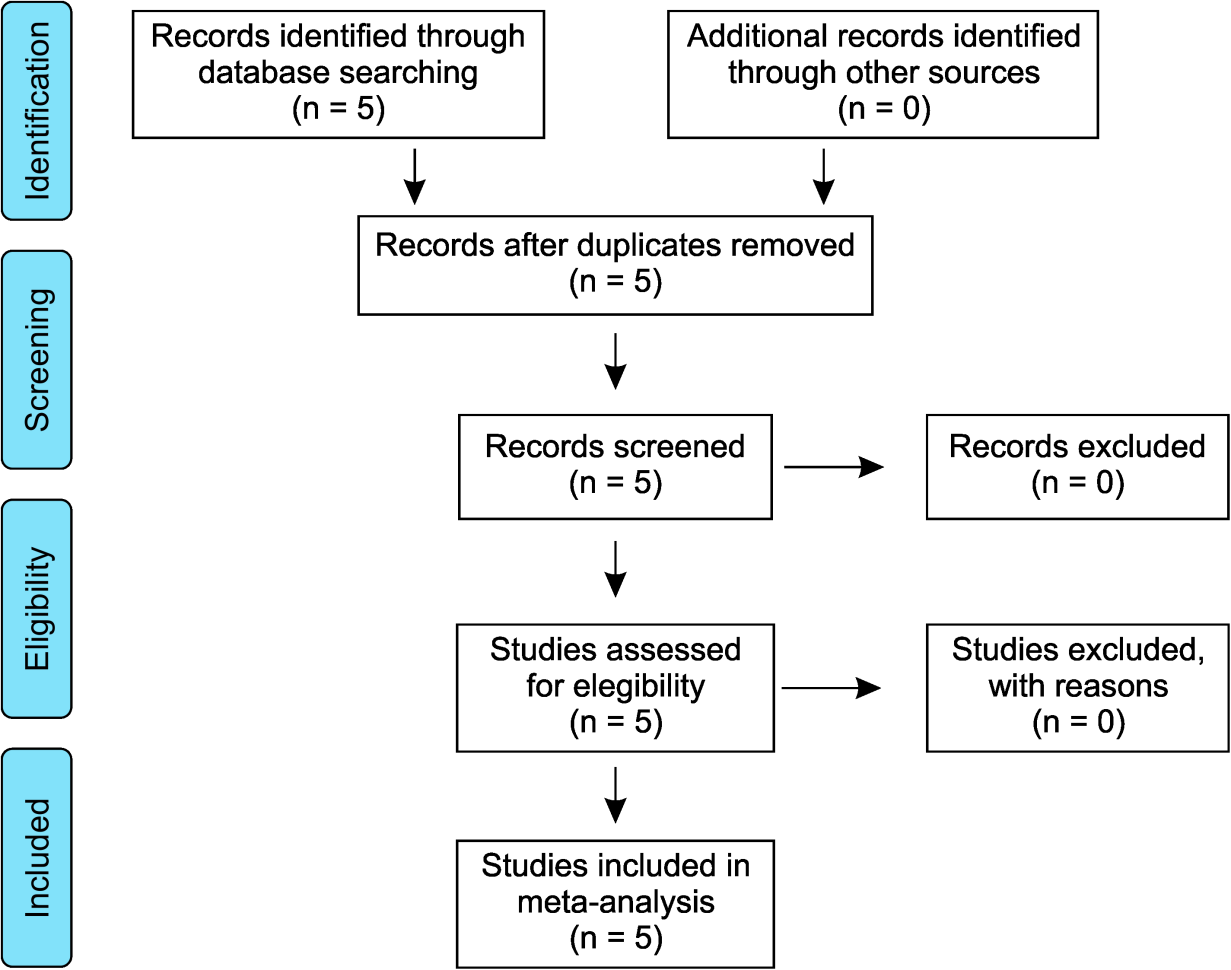
Prisma flow diagram.

To define a unique MPNST vs. NF gene signature that integrates data from diverse expression studies, we sought cited accessions in GEO and ArrayExpress databases that include MPNST and NF samples, and found five microarray studies (four human and one murine). These studies are referenced in Fig 1 and the heterogeneity of these data sets is detailed in Table 1 (bold). Despite the different number of genes represented by each microarray platform, all of them satisfied the selection criteria, segregated properly NF and MPNST samples, and supported relevant works that identified potential biomarkers and therapeutic targets [3,10,16,17] or discriminated neurofibromas and MPNST from other mesenchymal tumors [15]. Based on expression profile similarity [3], we grouped dNF and pNF samples from studies E-TABM-69, GSE41747 (human) and GSE66743, respectively. We also grouped segregated sporadic and NF1-associated MPNST samples from the GSE66743 study, as there were no differentially expressed genes between samples [10]. The final number of MPNST and NF samples compared is shown in Table 1; see Materials and methods for details of sample selection and preprocessing, and probe mapping to ENSEMBL genes as common identifiers.

**Table 1:** Microarray studies selected from public databases and included in the MPNST vs. NF meta-analysis.

Tissue	Organism	NF vs. control			MPNST vs. control			MPNST vs. NF			Reference	Platform	Probe Number
		Accession	Samples		Accession	Samples		Accession	Samples				
			NF	control		MPNST	control		MPNST	NF			
Cell cultures	Human	GSE14038^1^	22	10	GSE14038^1^	13	10	GSE14038^1^	22	10	[3]	Affy U133 Plus 2.0	54675
					GSE39764^1^	3	3				[1]	Agilent-014850 4x44K	45015
Nerve tumors	Human	GSE41747^1^	26	3	GSE41747^1^	6	3	**E-MEXP-353**^**2**^	**4**	**14**	[15]	Affy U133A	22283
								**E-TABM-69**^**2**^	**4**	**16**	[16]	Agilent 011521 G4110A	19061
								**GSE41747**^1^^**,**^^**3**^	**6**	**26**	[17]	Affy U133 Plus 2.0	54675
								**GSE66743**^1^	**30**	**8**	[10]	ABI Version 2	33025
	Mouse	GSE41747^1^	15	15	GSE41747^1^	18	15	**GSE41747**^**1**^	**18**	**15**	[17]	Affy 430 2.0	45101

^1^Accession number from GEO database.

^2^Accession number from ArrayExpress database.

^3^Data included in GSE14038 accession.

### MPNST vs. NF gene expression signature

**Fig 2:**
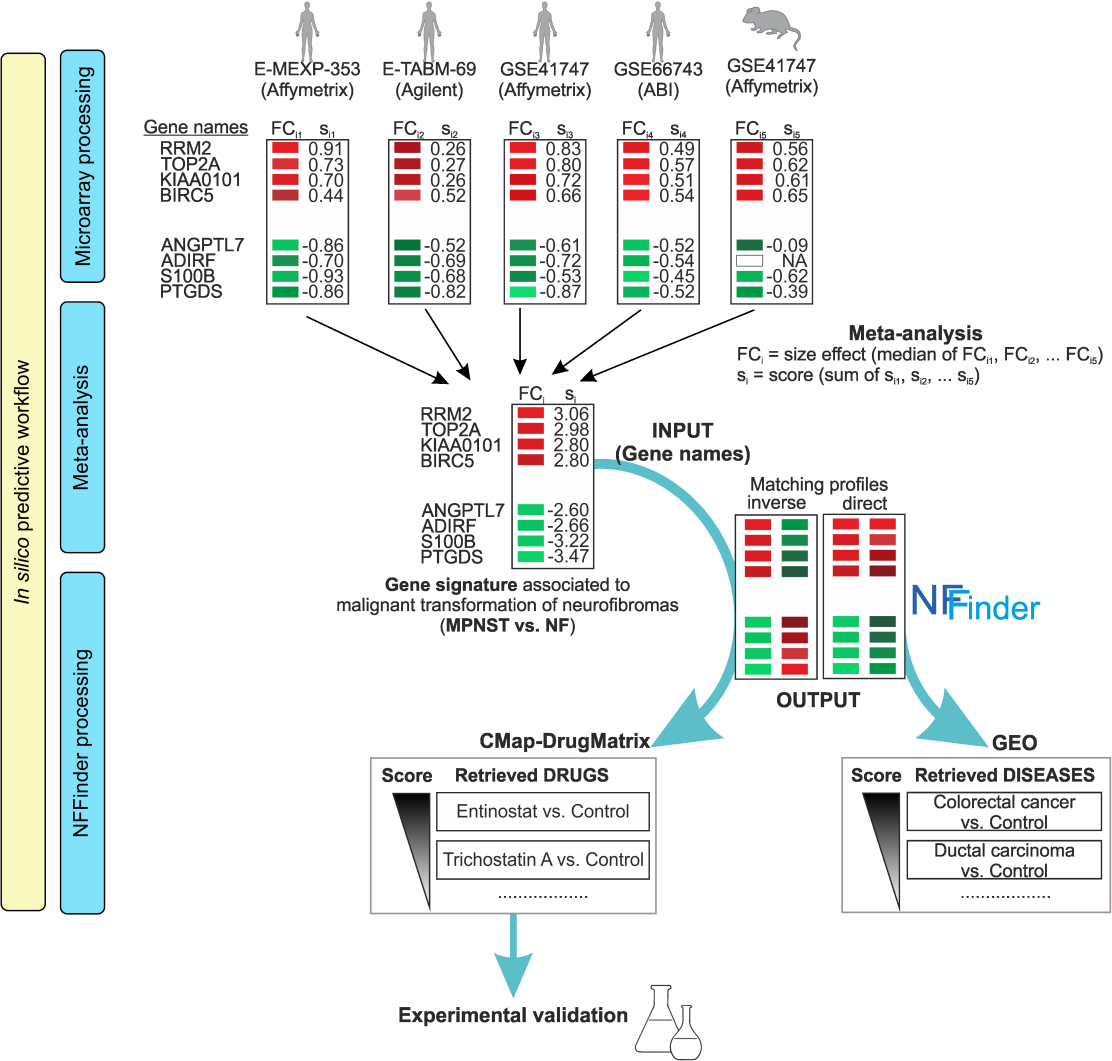
General schema of the integration of studies to generate the MPNST vs. NF gene signature and its processing by NFFinder. The upper panel shows the five microarray studies used to compute the MPNST vs. NF gene signature by meta-analysis (central panel). Score values calculated for each gene in each study (s_ij_) and for each signature gene (s_i_) are shown next to rectangles that indicate the respective gene size effect or fold change (FC_ij_ and FC_i_); red for upregulated genes and green for downregulated ones. The lower panel describes the results obtained from NFFinder when GEO and CMap-DrugMatrix databases are interrogated for direct or inverse matching of gene expression patterns, respectively, by using the signature gene names as input. Experimental validation should verify the hypotheses generated by this in-silico predictive workflow.

To integrate the five data sets, we calculated a score for each gene in each experiment (Fig 2, Microarray processing, and S1 Table), based on a formula used to combine data from heterogeneous sources [23]. To obtain a single score (s_i_) for each gene, we added individual scores (s_ij_) across human and mouse data sets (Fig 2, Meta-analysis). The final MPNST vs. NF gene signature contained 579 unique ENSEMBL human genes with non-null score and absolute median logFC value >0.99. The 336 up- and 243 downregulated genes included in this signature are highlighted in Table A in S2 Table (bold), embedded in the larger list from which the gene signature was filtered. The list includes the starting 7064 unique ENSEMBL human genes (4059 up- and 3005 downregulated) with non-null score in at least one independent study for which s_ij_ was computed. The comparison of non-null score profiles of this unfiltered integrative gene list and the lists derived from each individual study revealed comparable and overlapping patterns of gene scores (S1 Fig). Genes with the highest absolute scores in each study were at both ends of the unfiltered list, and were thus included in the final gene signature. However, some mice genes showed opposite behavior to that set in human, which corroborated differences in transcriptional responses between human and mouse models, particularly in neurodegenerative diseases [27]. Given the difficulty of interspecies comparison of results, we restricted the number of murine genes used to compute final scores (for score computation details, see Materials and methods).

The most promising biomarkers, 20 up- and 20 downregulated genes with the highest and the lowest score values from the MPNST vs. NF signature, were extracted from Table A in S2 Table (Table 2). In addition to gene score values, we determined attributes for estimating the relevance of each gene in the list, such as the median value of effect size, computed regarding the mean (logFC) or the median (logFC_m) across studies, as well as to assess the bias across the studies, like the number of studies in which each gene is represented, the inclusion or exclusion of mouse data, and the Bhattacharyya distance (BD) ratio, which replaced the entropy used by Rasche et al. [23] as an estimator of the homogenous contribution of each study to the final score (see Materials and methods) (Table A in S2 Table). The relationships between gene scores and additional attributes (Results A in S1 Appendix) and the final contribution of each individual experiment to the gene signature (Results B in S1 Appendix) are also evaluated.

**Table 2:** List of 20 genes with the highest and the lowest scores of the MPNST vs. NF signature^1^.

ENSGENE^2^	hgnc_symbol^3^	chrom_name^4^	band	score	logFC^5^	logFC_m^6^	studies^7^	mouse^8^	BD_ratio^9^
ENSG00000171848	RRM2	2	p25.1	3.06	4.19	4.47	5	1	13.68
ENSG00000131747	TOP2A	17	q21.2	2.98	3.73	3.94	5	1	10.01
ENSG00000166803	KIAA0101	15	q22.31	2.80	4.02	4.08	5	1	8.80
ENSG00000089685	BIRC5	17	q25.3	2.80	3.13	3.22	5	1	5.24
ENSG00000137804	NUSAP1	15	q15.1	2.59	3.67	3.75	5	1	16.50
ENSG00000185686	PRAME	22	q11.22	2.50	3.43	3.65	4	0	7.08
ENSG00000149948	HMGA2	12	q14.3	2.44	3.54	3.57	5	1	21.31
ENSG00000117724	CENPF	1	q41	2.43	3.08	3.20	5	1	9.38
ENSG00000198901	PRC1	15	q26.1	2.43	3.05	3.14	5	1	16.71
ENSG00000157456	CCNB2	15	q22.2	2.34	2.99	3.11	5	1	14.17
ENSG00000128045	RASL11B	4	q12	2.19	3.39	3.81	4	0	17.65
ENSG00000156076	WIF1	12	q14.3	2.17	4.70	4.99	4	0	22.10
ENSG00000134057	CCNB1	5	q13.2	2.16	2.54	2.48	5	1	20.67
ENSG00000066279	ASPM	1	q31.3	2.15	2.93	3.02	5	1	11.88
ENSG00000088325	TPX2	20	q11.21	2.14	2.96	3.19	5	1	23.98
ENSG00000123975	CKS2	9	q22.2	2.07	2.21	2.30	5	1	17.55
ENSG00000143476	DTL	1	q32.3	2.07	3.20	3.30	5	1	26.58
ENSG00000170312	CDK1	10	q21.2	2.01	3.12	3.17	4	1	17.50
ENSG00000007062	PROM1	4	p15.32	1.99	4.19	4.54	4	0	12.37
ENSG00000115163	CENPA	2	p23.3	1.94	2.77	2.93	5	1	18.19
ENSG00000021300	PLEKHB1	11	q13.4	-1.66	-2.96	-3.13	5	1	35.86
ENSG00000109846	CRYAB	11	q23.1	-1.68	-3.13	-3.20	5	1	38.39
ENSG00000100146	SOX10	22	q13.1	-1.74	-2.81	-2.93	5	1	35.15
ENSG00000100307	CBX7	22	q13.1	-1.79	-2.33	-2.37	5	1	18.08
ENSG00000149218	ENDOD1	11	q21	-1.82	-2.40	-2.44	4	1	18.78
ENSG00000197766	CFD	19	p13.3	-1.87	-2.98	-2.53	5	1	43.75
ENSG00000148180	GSN	9	q33.2	-1.90	-2.60	-2.58	5	1	7.053
ENSG00000134121	CHL1	3	p26.3	-1.94	-3.63	-3.36	5	1	32.93
ENSG00000172005	MAL	2	q11.1	-1.97	-3.05	-3.24	5	1	16.49
ENSG00000174944	P2RY14	3	q25.1	-2.01	-3.39	-3.47	5	1	35.95
ENSG00000108381	ASPA	17	p13.2	-2.06	-2.29	-2.40	5	1	31.63
ENSG00000168477	TNXB	6	p21.32	-2.07	-3.13	-3.34	5	1	26.58
ENSG00000127951	FGL2	7	q11.23	-2.08	-3.92	-3.75	5	1	27.43
ENSG00000196616	ADH1B	4	q23	-2.08	-3.86	-3.88	5	1	38.97
ENSG00000071991	CDH19	18	q22.1	-2.15	-4.21	-4.01	5	1	33.78
ENSG00000147588	PMP2	8	q21.13	-2.33	-4.39	-4.80	5	1	14.94
ENSG00000171819	ANGPTL7	1	p36.22	-2.60	-3.85	-4.12	5	1	18.30
ENSG00000148671	ADIRF	10	q23.2	-2.66	-3.81	-4.00	4	0	3.22
ENSG00000160307	S100B	21	q22.3	-3.22	-4.31	-4.38	5	1	11.91
ENSG00000107317	PTGDS	9	q34.3	-3.47	-4.89	-4.85	5	1	7.88

^1^The complete MPNST vs. NF gene signature of 579 genes, embedded in the unfiltered list of 7064 genes, is bold-highlighted in Table A in S2 Table.

^2^ENSEMBL gene ID.

^3^Gene symbol from HUGO Gene Nomenclature Committee.

^4^Human chromosome name.

^5^Median of logFC_ij_ computed for each gene across the studies.

^6^Median of logFC_m_ij_ computed for each gene across the studies.

^7^Number of studies included in the MPNST vs. NF meta-analysis.

^8^Inclusion (1) or exclusion (0) of mouse data in MPNST vs. NF meta-analysis. Exclusion may be due to the absence of mouse data or because current data differed from human data.

^9^Bhattacharyya distance ratio.

### *In silico* search for therapeutic drugs to reverse malignant phenotype

Analysis of similar or opposite gene signatures is one of the most important applications of gene signatures for generating hypotheses for the study of NF1 and other rare diseases. We used NFFinder [20] to explore repurposing of drugs that might reverse the NF1-associated MPNST malignant phenotype by inspecting CMap and DrugMatrix databases for gene expression patterns opposite to the MPNST vs. NF gene signature (Fig 2, NFFinder processing). The complete score-ranked list of drugs with pval <0.005 can be seen at goo.gl/IdyV1N; Table A in S3 Table shows the first 50 drug entries retrieved. In the top two positions was Entinostat MS-275, a histone deacetylase (HDAC) inhibitor selective for class I HDAC. The non-specific HDAC inhibitor Trichostatin A (TSA) appeared in 39 of the top 50 entries retrieved. Two other HDAC inhibitors, the class I-selective HC-toxin and the non-specific HDAC inhibitor Scriptaid, were also on the shortlist. Other anti-cancer compounds were rifabutin, an antibiotic effective against lung cancer cells, PNU-0251126, which correlated positively with drugs for leukemia treatment, the protein phosphatase 2A inhibitor cantharidin, which induces cell death, the anti-inflammatory steroid medrysone, the topoisomerase II inhibitor ellipticine, a potent antineoplastic agent, and the non-steroidal selective estrogen receptor (ER) modulator tamoxifen, used to treat ER-positive breast cancer. To identify conditions similar to the NF-to-MPNST transformation that might share therapeutic treatments, we used NFFinder to search for disease gene signatures from GEO experiments with expression patterns resembling the MPNST vs. NF gene signature; results are shown in  goo.gl/36xlB1 and the first 50 GEO studies are summarized in Table B in S3 Table. The most similar diseases were other types of cancer (58%), of which 73% were solid tumors, especially in prostate and breast, 20% were leukemias and lymphomas, and 7%, tumor cell lines. We also found premalignant neoplasias of epithelial tissue in endometrium and kidney (12%), lipid metabolism conditions (6%), pulmonary diseases (4%), muscular dystrophy (4%) and neuronal conditions (2%).

The experimental validation of the therapeutic effectiveness of drugs retrieved by NFFinder to treat NF1-associated MPNST was reported for HDAC inhibitors [28], cantharidin [29] and tamoxifen [30], and clinical trials have been suggested for all of them. The conclusion of our in-silico workflow (Fig 2) with these experimental data confirms the predictive value of the MPNST vs. NF gene signature and its usefulness to identify robust biomarkers and therapeutic agents in neurofibromatosis disease.

### Functional characterization of the MPNST vs. NF gene signature

We provide detailed lists of GO term enrichment of MPNST vs. NF up- and downregulated genes (Tables A to F in S4 Table), as well as over-represented pathways derived by analysis of KEGG (Tables G and H), Wiki (Tables I and J), and Reactome (Tables K and L) databases. Results C in S1 Appendix details genes in the pathways identified.

For upregulated genes, DNA replication and cell cycle pathways were shared in the three pathway databases; 70% of GO terms associated to these genes were thus involved mainly in processes that underlie positive regulation of cell proliferation, mitosis and meiosis. We also found terms related to morphogenesis and development of skeletal, nervous, cardiovascular and digestive systems. GO terms directly related to malignancy of proliferating cells included collagen catabolic process, which can affect extracellular matrix (ECM) organization, a process involved in epithelial to mesenchymal transition (EMT) and cell migration. The KEGG ECM-receptor interaction pathway was also over-represented, which supports this observation. In accordance with GO terms and pathways, components from nucleus and cytoplasm were associated predominantly with upregulated genes.

For the downregulated genes, all three pathway databases identified the immunity pathway linked to complement activation, including regulatory elements and complement components. Downregulated genes also appeared to control peripheral nervous system development, particularly *via* myelination and axonogenesis. Other downregulated genes participated in the response to steroid hormones and in cell migration, chemotaxis, and cell adhesion to another cell or to a substrate such as the ECM. Unlike upregulated genes, downregulated genes accumulated GO terms related to plasma membrane and its intrinsic components, including elements from proteinaceous ECM involved in cell junction formation. When we analyzed the unfiltered list of 3005 downregulated genes, from which we derived the MPNST vs. NF downregulated gene signature, we found that specific KEGG pathways in cancer were over-represented (Table P in S4 Table).

In accordance with this functional characterization, most upregulated genes from Table 2 are involved in cell cycle progression, have been linked with carcinogenic processes in which they are upregulated, and many have also been described as diagnostic biomarkers (S5 and S6 Tables). HMGA2 is the only gene product that is an architectural transcription factor, although some other genes act as regulatory elements in mitosis, especially cyclins CCNB2, CCNB1, and kinase CDK1. As anticipated, and in contrast to the mitosis-related products of upregulated genes, which were found mainly in nucleus and cytoskeleton, the 20 most-downregulated gene products (Table 2) act mainly in the extracellular space. Whereas upregulated genes are overexpressed in cancer, some downregulated genes can be silenced or overexpressed, and thus have dual roles in cancer, as tumor suppressors or cell proliferation promoters. Some of these genes appear to be involved in signal transduction and development in the nervous system *(PLEKHB1*, *SOX10*, *CHL1*, *PTGDS)*, in maintenance of structural integrity of cells and tissues, e.g., ECM formation or myelin synthesis *(CRYAB*, *CBX7*, *GSN*, *MAL*, *TNXB*, *CDH19*, *PMP2*, *ANGPTL7*, *ADIRF)*, or to have various roles in the immune response *(ENDOD1*, *P2RY14*, *FGL2*, *S100B)*. SOX10 and S100B coregulate Schwann cell proliferation and myelination [31]. CBX7 is involved in epigenetic transcriptional repression. The metabolic proteins ASPA and ADH1B are also in the most-downregulated group. ASPA participates in increasing the pool of acetate, an essential precursor for histone acetylation reactions. ADH1B oxidizes alcohol, thus also helping to generate acetate precursors, as well as retinol, an early step in synthesis of retinoic acid, a basic molecule in epithelial tissue growth and differentiation.

### Chromosome distribution of genes in the MPNST vs. NF signature

**Fig 3:**
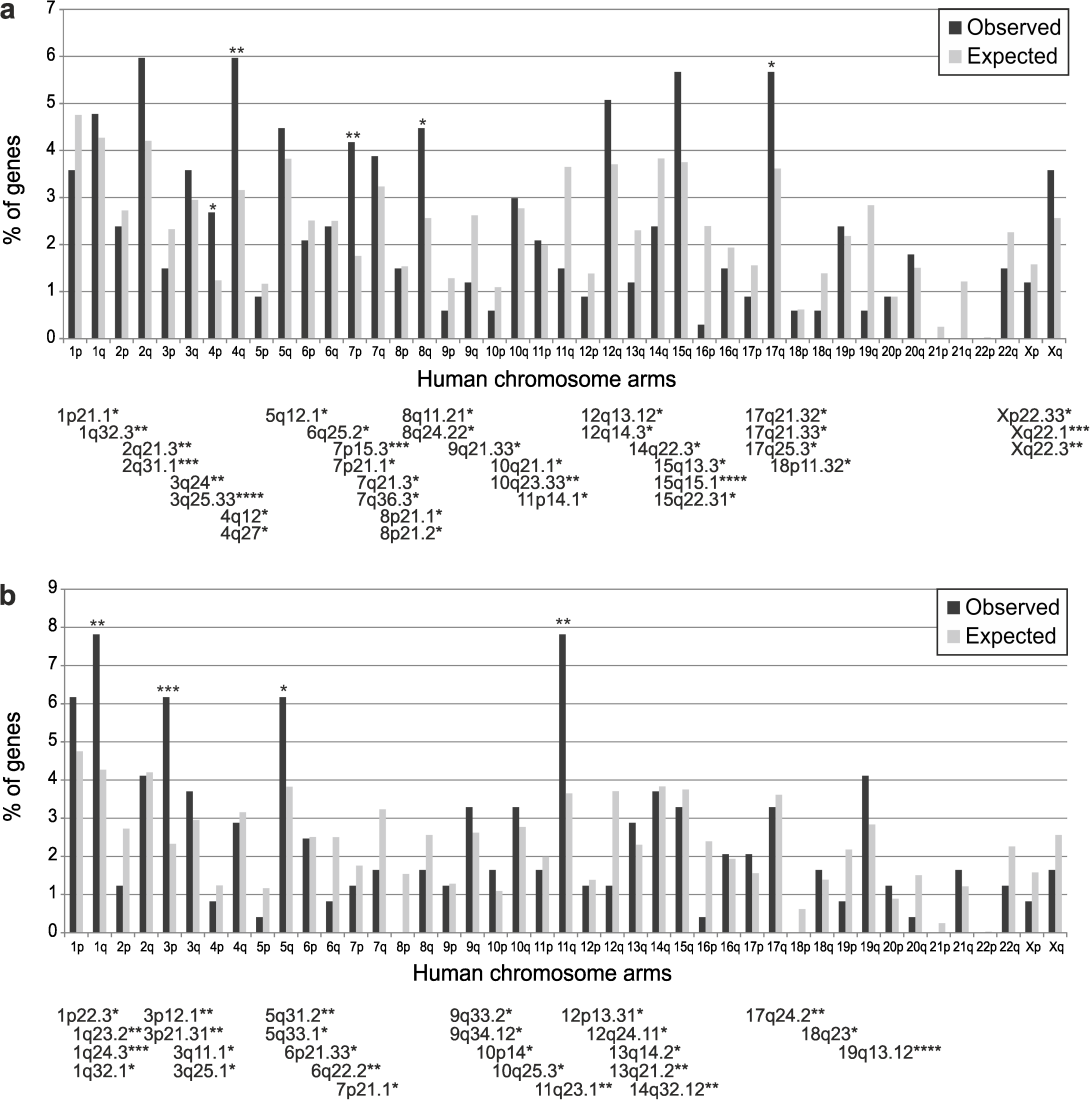
Chromosome distribution of the MPNST vs. NF gene signature. The gene signature distribution was calculated from the 336 genes with positive score **(a)**, and from the 243 genes with negative score **(b)**. Bar diagrams compare the observed distribution of MPNST vs. NF gene percentage in the human chromosome arms (dark bars) with the expected distribution according to the human ENSEMBL database (light bars). Statistical significance of the gene signature over-represented chromosome arms is above the bars. Over-represented human chromosome bands in the MPNST vs. NF gene signature are shown below each chart. Their statistical significance is shown at the top right side of band names. (****) P(X≥x) < 0.0001, (***) 0.0001< P(X≥x) < 0.001, (**) 0.001< P(X≥x) < 0.01, (*) 0.01< P(X≥x) < 0.05.

To establish whether there are significant distribution differences in human chromosomes for genes in the MNPST vs. NF signature (336 up- and 243 downregulated genes), we computed frequencies of the number of genes per chromosome arm from the gene signature and the whole human genome (Fig 3). Upregulated genes accumulated significantly in chromosome arms 4q, 7p, 8q and 17q, whereas downregulated genes were over-represented mainly in 1q, 3p, 5q and 11q. Another binomial distribution identified chromosome bands in which both up- and downregulated genes were over-represented. The highest concentration of upregulated genes was found in bands 3q25.33 and 15q15.1, with others concentrated in bands 7p15.3 and Xq22.1; the largest number of bands with over-representation of upregulated genes was seen in arm 15q and the distal part of 17q. The highest significant concentration of downregulated genes was found in bands 1q24.3 and 19q13.12; arm 1q showed the largest number of over-represented bands. The function of genes in over-represented chromosome regions is shown in S7 Table. Enrichment in GO biological process terms coincided with the functions of up- and downregulated genes in the MPNST vs. NF signature, as described above.

Comparison of chromosome region enrichment from the MPNST vs. NF gene signature with that in S2 Fig for the unfiltered gene list indicated some shared over-represented regions; chromosome arms 7p, 8q and 17q accumulated upregulated genes on the unfiltered list, whereas arms 3p and 11q concentrated downregulated genes. The distal part of chromosome arm 17q showed the largest number of over-represented bands. In addition, the unfiltered list showed over-represented upregulated genes in arms 1q, 2p, 2q, 6p, 7q, 19p and Xq, and downregulated genes in 1p, 9q, 10q, 14q, 17p and 20p.

### Panels of biomarkers with similar expression profiles in the context of NF1 disease

Given the greater potential prognostic capacity of biomarker panels compared to individual biomarkers, we built robust gene profiles to group functionally related genes with similar expression patterns. To obtain gene profiles, we extended the gene score methodology to identify other average gene signatures in the NF1 context. We performed five additional differential expression analyses; Table 1 groups the accession numbers of the studies used to determine gene signatures for the five new comparisons. Two of these new comparisons involved nerve tumors, whereas the other three comprised cell cultures. For tumor tissue, we carried out two meta-analyses that integrated human and mouse nerve tumors by comparing NF vs. control (nerve tissue) and MPNST vs. control. For cell cultures, we made three comparisons. Only the comparison of MPNST cell lines vs. control (NHSC; normal human Schwann cells) integrated data from two studies; for the other two comparisons, primary NFSC (neurofibroma Schwann cells) vs. control (NHSC) and MPNST cell lines vs. primary NFSC, we used standard differential expression analyses, as only one study was available. These additional gene signatures were added as Tables B to J in S2 Table. Their composition, and a comparison with the MPNST vs. NF gene signature are detailed in Results D in S1 Appendix.

**Fig 4:**
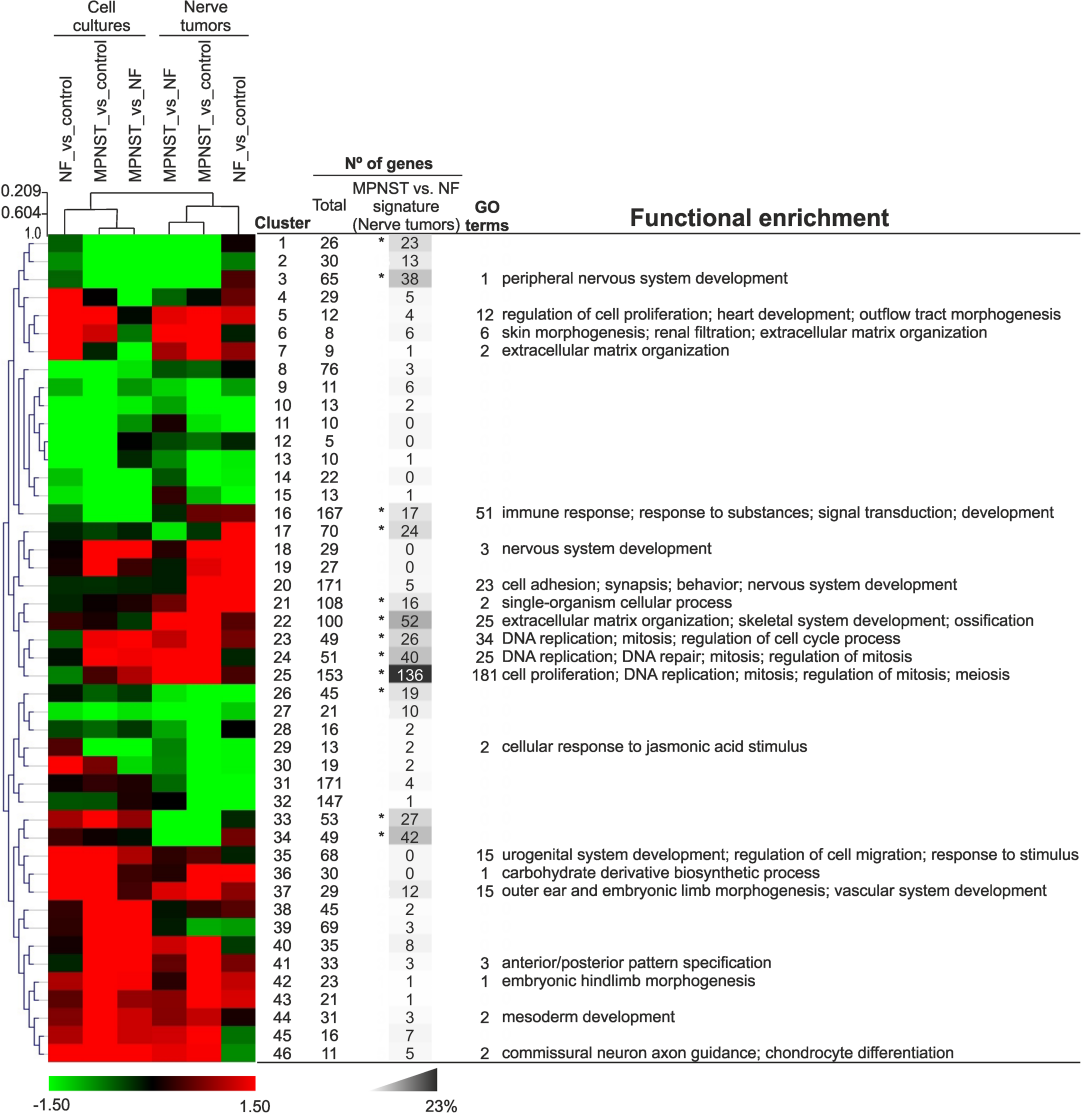
Clustering of phenotype comparisons and of NF1-related genes. Comparisons among MPNST, NF and control phenotypes were grouped through hierarchical clustering. Cell culture and nerve tumor comparisons are shown on the top of the upper dendrogram, and the node height scale is detailed on the left of this tree. The hierarchical relationship among gene clusters obtained by grouping the logFC_m values of 2209 NF1-related genes by the Self-Organizing Tree Algorithm (SOTA) is represented by the dendrogram on the left. Clusters are described by their SOTA centroid vectors. Color scale of logFC_m values is shown below. The right side of the Fig details the number of genes in each cluster, the gene number of the MPNST vs. NF signature in each cluster (*, > 15 genes), the percentage of genes of this signature in each cluster (grey scale shown below), the number of biological process GO terms over-represented in each cluster, and the summary of the GO term enrichment as functional characterization of clusters. A complete list of terms is shown in S9 Table.

To search for function-related genes in the context of neurofibromatosis, we sought genes with similar expression patterns in the six comparisons of NF, MPNST and control phenotypes in cell cultures and nerve tumors; S8 Table shows the list of 2209 genes obtained by gathering signatures of these comparisons. Comparisons (clustered by tissue type) and genes grouped independently, and dendrograms were determined of the comparisons and of the genes, grouped in 46 clusters represented by their self-organizing tree algorithm (SOTA) centroid vectors (Fig 4). Divergence between sample tissue types coincided with dissimilarities in the functional characterization of genes in the cell culture and nerve tumor signatures (Results D in S1 Appendix). As predicted, the second line of sample divergence separated MPNST samples (vs. NF or vs. control) from NF vs. control comparisons. Results E in S1 Appendix evaluates gene pattern homogeneity in each cluster.

Although almost every cluster contained genes from the MPNST vs. NF tumor tissue signature (highlighted in S8 Table), the most populated clusters (>15 genes) appeared in four cluster areas, from which were derived four biomarker panels that included the majority of the 20 most up- and downregulated genes (Fig 4). Cluster 3, which includes genes involved in development of the peripheral nervous system, contained six genes of the 20 most-downregulated (Table 2), *CRYAB*, *SOX10*, *FGL2*, *CDH19*, *PMP2* and *S100B*; *SOX10* and *S100B* are markers used to diagnose neural crest-derived tumors [32]. Cluster 17 incorporated three of the most-downregulated genes, *PLEKHB1*, *GSN* and *CHL1*. Cluster 25 included 153 genes and 181 GO terms, and thus concentrated the highest functional enrichment. This cluster, with adjacent clusters 23 and 24, accumulated the majority of genes involved in cell proliferation. Cluster 25 included 17 of the 20 genes with the highest scores from the MPNST vs. NF comparison (Tables 2 and S8), particularly the genes that encode the putative NF1 prognostic markers *TOP2A* and *BIRC5* [10]. Finally, cluster 34 contained five of the 20 most-downregulated genes in the MPNST vs. NF signature, *CBX7*, *P2RY14*, *TNXB*, *ADIRF* and *PTGDS*. Details for gene accumulation in clusters from the MPNST vs. NF signature, and for the five additional signatures, are shown in S3 Fig.

### Panel of hypermethylated biomarkers in the context of NF1 disease

Aberrant epigenetic changes are associated to most cancers, particularly hypomethylation of oncogenes and hypermethylation of tumor suppressors. To establish relationships between gene expression and aberrant promoter methylation, we associated our six gene expression signatures with the methylation status of gene promoters. In GEO and ArrayExpress databases, we sought studies that provide raw methylation data relative to NF1 evolution. GEO accession GSE21714 genome-wide methylome analysis compared immunoprecipitated DNA from 10 pooled MPNST samples, 10 pooled NF samples, and 6 pooled control Schwann cell samples [13]; for the process used to identify methylated promoters, see Materials and methods. The edgeR.logFC and edgeR.adj.pval (<0.1) values obtained for MPNST vs. NF (Table A in S2 Table), MPNST vs. control (Table B), and NF vs. control (Table C) methylome comparisons showed 428, 594 and 9 differentially methylated gene promoters, respectively (right columns); most were hypermethylated. MPNST vs. NF and MPNST vs. control comparisons showed 181 promoters in common, and MPNST vs. control and NF vs. control Schwann cells indicated 4 shared promoters.

Hypermethylation of the *RASSF1A* promoter identified a NF1-associated MPNST subgroup with poor prognosis [14]; we therefore tested *RASSF1* promoter methylation status as a control of our analysis. The *RASSF1* promoter was differentially hypermethylated in MPNST vs. NF and in MPNST vs. control Schwann cells, but not in NF vs. control Schwann cells. To find other potential regulatory elements with aberrant promoter methylation, we searched for other downregulated genes with hypermethylated promoters in MPNST vs. NF and MPNST vs. control Schwann cell comparisons. Using the unfiltered gene lists for all comparisons (Table J in S2 Table), we selected genes whose expression pattern in tumor tissue included a negative MPNST vs. NF score, as well as logFC values < -0.5 for MPNST vs. NF and MPNST vs. control comparisons. Genes selected also showed edgeR.logFC and edgeR.adj.pval promoter methylation values >1.5 and <0.1, respectively. We found 56 gene promoters that fit these selection conditions. The *RASSF1* gene and 9 of the selected genes found in the MPNST vs. NF gene signature are listed in Table 3; the full list is given in S10 Table.

**Table 3:** MPNST vs. NF signature genes potentially down-regulated by promoter hypermethylation^1^.

ENSGENE^2^	hgnc symbol^3^	ch^4^	band	NF vs. control				MPNST vs. control						MPNST vs. NF					
				Cell cultures		Nerve tumors		Cell cultures		Nerve tumors				Cell cultures		Nerve tumors			
				score	logFC^5^	score	logFC^5^	score	logFC^5^	score	logFC^5^	*edgeR*^6^		score	logFC^5^	score	logFC^5^	*edgeR*^6^	
												*logFC*	*adj*.*pval*					*logFC*	*adj*.*pval*
ENSG00000068028	RASSF1	3	p21.31	0	-0.36	-0.08	-0.47	-0.07	-0.91	0	-1.07	3.35	1.64e-03	-0.03	-0.70	-0.09	-0.70	2.77	1.30e-02
ENSG00000159200	RCAN1	21	q22.12	0	-0.93	0	0.73	-0.76	-3.18	0	-1.11	2.99	1.88e-07	-0.19	-2.37	-0.64	-2.08	2.41	1.28e-02
ENSG00000197971	MBP	18	q23	-0.56	-4.38	-1.28	-4.28	-1.69	-5.89	-1.54	-7.13	3.80	6.60e-59	-0.02	-2.99	-0.69	-2.96	3.41	1.06e-53
ENSG00000175785	PRIMA1	14	q32.12	0	-2.20	0	-0.32	-0.58	-2.75	-0.67	-2.40	3.09	5.52e-07	0	-1.43	-0.71	-2.43	2.87	4.01e-05
ENSG00000152061	RAGAP1L	1	q25.1	0	0.50	0	-0.40	0.06	1.11	-0.24	-1.94	2.55	5.11e-06	0	0.86	-0.73	-1.54	2.24	1.74e-05
ENSG00000213088	ACKR1	1	q23.2	0	0.07	0	-0.77	0	-0.02	-0.78	-3.15	2.03	2.30e-04	0	0	-1.00	-2.84	3.33	1.04e-02
ENSG00000158887	MPZ	1	q23.3	0	-1.83	0	-1.45	-1.22	-4.66	-1.01	-5.09	4.37	1.95e-08	-0.70	-5.51	-1.11	-2.49	2.27	3.46e-03
ENSG00000070731	ST6GALNAC2	17	q25.1	-0.18	-1.97	-0.25	-1.26	-1.33	-4.69	-0.69	-2.93	2.74	4.47e-06	-0.47	-3.54	-1.56	-2.81	2.01	1.31e-02
ENSG00000132470	ITGB4	17	q25.1	0	0.02	0	-0.62	0	0.16	-0.55	-2.29	2.16	4.77e-02	0	-0.12	-1.62	-3.13	1.90	6.70e-05
ENSG00000160307	S100B	21	q22.3	0	-1.40	0	-0.72	-1.81	-8.33	-1.06	-4.94	2.37	6.82e-04	-0.86	-7.66	-3.22	-4.31	2.56	2.02e-02

^1^Data extracted and ordered from gene list showed in Table J in S2 Table. The complete list of genes potentially down-regulated by hypermethylation in MPNST vs. NF and vs. control comparisons is shown in S10 Table.

^2^ENSEMBL gene ID.

^3^Gene symbol from HUGO Gene Nomenclature Committee.

^4^Human chromosome name.

^5^Median of logFC_ij_ computed for each gene across the studies comparing two phenotypes.

^*6*^*edgeR*.*logFC* and *edgeR*.*adj*.*pval* computed by the analysis of promoter methylation. NA values of *edgeR*.*logFC* and *edgeR*.*adj*.*pval* were omitted for NF vs. control comparison.

## Discussion

### MPNST vs. NF gene expression signature

Here we report the integrative MPNST vs. NF gene signature associated to the NF-to-MPNST transition in the context of neurofibromatosis type 1 disease. For this signature, filtered from a larger list of all differentially expressed genes from five studies comparing MPNST to NF, we included genes with the highest scores, size effects and consensus among individual studies, and used it as a core to study biomarkers and drugs that might control evolution to malignity in NF1 patients.

The 20 highest and 20 lowest score-ranked genes suggested as promising biomarkers (Table 2) are all implicated in carcinogenic processes and many are suggested biomarkers. Among the top 20 genes in the MPNST vs. NF signature are 4 genes reported as induced in the transition from benign NF to MPNST (S11 Table); their products are the topoisomerase TOP2A, needed for correct chromosome segregation in mitosis, the apoptosis inhibitor BIRC5, a member of the mitotic chromosome passenger complex, the architectural transcription regulator HMGA2, and TPX2, which is essential for correct mitotic spindle assembly and activates the AURKA kinase to control cell cycle progression. Among the 20 most-downregulated genes are 4 NF- and/or MPNST-associated genes (S12 Table), the transcriptional regulator SOX10, needed for neural crest multipotent cell and peripheral nervous system development, the extracellular matrix glycoprotein TNXB, the cell-cell adhesion Ca^2+^-dependent cadherin CDH19, specific to myelin-forming cells, and the Ca^2+^-binding S100B, whose inhibition is associated to MPNST transformation [33].

The *NF1* gene, the basic reference of reliability for the gene signature generated, had a null score and was thus absent from the MPNST vs. NF signature. This suggested that most samples of sporadic MPNST had somatic deactivating *NF1* mutations, which corroborates previous studies [34]. Our results coincide with gene expression data that compare MPNST with benign NF, although given the strict score threshold, some known MPNST-related genes on the unfiltered gene list were lacking in the gene signature, such as *SOX9*, which encodes a developmental transcription factor [3], and *TNC*, which is involved in axon guidance during development [16]. Other cancer-related genes were absent from the signature for similar reasons, such as *CDH1*, which codes for the tumor suppressor cadherin 1. CDH1 silencing is a marker of the EMT associated to cell proliferation, invasion and metastasis [35].

We found that despite some exceptions, our results for over-represented chromosome regions in the MPNST vs. NF gene signature corroborated previous data on chromosome region enrichment. In addition, we identified other regions such as chromosome arm 3p, in which many downregulated genes are concentrated. A detailed comparison of these regions and reported aberrant chromosome modifications is shown in Discussion A in S1 Appendix.

### Biomarker panels that share NF1-related gene expression profiles

In addition to the study of individual genes as potential biomarkers, tests of groups of genes in the MPNST vs. NF signature can improve prospects for diagnosis or prognosis. Kolberg et al. [10] defined a prognostic test for post-tumor resection MPNST patients, based on expression of three proteins encoded by genes located in the distal part of chromosome 17q *(BIRC5*, *TK1*, *TOP2A)*. These genes were included in a 31-gene cell cycle progression (CCP) signature that is a robust predictor of clinical outcome for prostate cancer patients [36].

Given the limitation of the MPNST vs. NF score-ranked gene signature for grouping genes with similar function, we built profiles that integrated other NF1-related signatures. These signatures, obtained from tumor tissue and cultured cells, were based on one or two studies each and were thus less robust than our original MPNST vs. NF, built from five studies. Genes were grouped by their profiles using SOTA, which allows hierarchical clustering by a neural network. We obtained a large number of SOTA clusters containing homogeneous groups of functionally related genes that shared similar expression profiles.

Of the upregulated genes in the MPNST vs. NF signature, most grouped to Cluster 25 and are linked to cell cycle progression. Mechanisms that regulate the complex process of cell cycling are precisely regulated, and deregulation drives to aberrant cell proliferation and cancer development [37]. Not surprisingly, many genes from the CCP signature [38] were included in the cluster 25. Our group of the 20 most-upregulated genes contained 9 of the 31 CCP signature genes (*RRM2*, *TOP2A*, *KIAA0101*, *BIRC5*, *NUSAP1*, *CENPF*, *PRC1*, *ASPM*, *DTL*), and 14 more CCP genes were upregulated in the MPNST vs. NF gene signature (*FOXM1*, *TK1*, *CDC20*, *BUB1B*, *PBK*, *CDKN3*, *ASF1B*, *CEP55*, *DLGAP5*, *RAD51*, *KIF11*, *KIF20A*, *PTTG1*, *CDCA8*). Due to its potential as MPNST biomarker [39], we propose the inclusion of *HMGA2* in any similar list built to interrogate the expression status of genes involved in cell cycle progression in MPNST prognosis. A panel from cluster 3 should contain at least *CRYAB*, *SOX10*, *FGL2*, *CDH19*, *PMP2*, and *S100B*. Cluster 17 genes to be evaluated would be *PLEKHB1*, *GSN* and *CHL1*. A cluster 34 panel should include at least *CBX7*, *P2RY14*, *TNXB*, *ADIRF* and *PTGDS* genes.

The divergence between expression values in comparisons of cell cultures and nerve tumors indicated clear differences due to the nature of the samples. Many essential characteristics of cancers in which the ECM plays a fundamental role, such as those involving EMT, invasion and metastasis, cannot be appropriately mimicked by cultured cells. In contrast to MPNST, which contain a mixture of different cell types, cultured cells derive from a single cell type. Differences between cultured cells and nerve tumors, exemplified by *SOX9*, *SUZ12*, *EGFR*, *SPP1* and *BMP2* genes, are detailed in Discussion B in S1 Appendix.

### Genes potentially silenced by hypermethylation of their CpG-island promoter region

To identify potential biomarkers whose expression is inhibited by hypermethylation, we sought genes that were downregulated and showed promoter hypermethylation in tumor tissue for MPNST vs. NF and MPNST vs. control comparisons; 42% of the differentially methylated promoters were shared, most of which were hypermethylated. This result correlated with gene expression data, as more than 58% of genes from the MPNST vs. NF showed similar behavior in the MPNST vs. control comparison. In addition to the tumor suppressor *RASSF1*, whose differential promoter hypermethylation status and expression inhibition are associated in MPNST and other cancers [14], we found additional regulatory genes. The genes identified by common expression and methylation patterns were implicated mainly in cancer, which corroborates the importance of epigenetic changes in the control and evolution of cancerous processes; they were also linked to immune response, nervous system development, lipid metabolism, metabolic energy balance and detoxification (S10 Table). Many act as mediators in signal transduction pathways, have a structural role, or interact with other proteins to control various biological processes related to cell proliferation and apoptosis. Most genes in the MPNST vs. NF signature that are potentially silenced by promoter hypermethylation are associated with structural functions in the peripheral nervous system (*PRIMA1*, *ST6GALNAC2*, *ITGB4*, *MBP*, *MPZ*) or with signal transduction pathways (*RABGAP1L*, *ACKR1*, *S100B*) (S10 Table, bold). Differentially methylated genes are discussed in detail in Discussion C in S1 Appendix.

### From MPNST vs. NF gene signature to working hypotheses: Therapeutic applications of HDAC inhibitors, cantharidin and tamoxifen

To identify diseases or biological problems similar to malignant NF evolution, or to define potential therapeutic drugs that could reverse malignant phenotype, we used NFFinder to compare the MPNST vs. NF signature with similar or contrasting signatures. Gene signatures in GEO studies most similar to that of MPNST vs. NF were associated with cancer, mainly solid tumors, followed by other diseases that share several phenotypic alterations with neurofibromatosis. Most signatures in CMap and DrugMatrix databases that contrasted from the MPNST vs. NF signature indicated that the reported as effective compounds to treat MPNST, cantharidin and tamoxifen, and especially HDAC inhibitors, could potentially reverse the malignant phenotype. Our in-silico prediction corroborates these data, which indicates the reliability of our gene signature as representative of NF evolution to malignancy.

HDAC inhibitors control gene expression by blocking deacetylation of histone and non-histone proteins. These inhibitors modify the chromatin condensation status [40] and also control several chromatin structure-independent processes that alter gene expression, such as transcription factor activity, miRNA expression, and signal transduction [41]. Some HDAC inhibitors act as epigenetic regulators by modifying the DNA methylation status, which reveals crosstalk between acetylation and methylation [42]. This ability of HDAC inhibitors to reverse epigenetic aberrations make them effective therapeutic agents in cancer, as well as in neurological and immune disorders [43]. In cancer, HDAC inhibitors impair cell proliferation, neoangiogenesis and metastasis, and increase differentiation and apoptosis. Blockade of tumor angiogenesis nonetheless hinders drug delivery and limits the use of these inhibitors for solid tumors [44]. Combination therapies are thus necessary.

Based on the sensitivity of Ras signaling tumors to HDAC inhibitors, López *et al*. assessed these compounds in MPNST, both *in vitro* and in tumor xenografts [28]. The strong reaction of NF1-associated MPNST cell lines to HDAC inhibitors led the authors to suggest their therapeutic value for inclusion in clinical trials. NFFinder also found that the pan-HDAC inhibitor TSA can replace the effective combination of synergy-acting compounds PD-901 and JQ1 to kill MPNST cells and shrink tumors [12,20]. Robust biomarkers are nonetheless needed to predict the effectiveness of TSA and other HDAC inhibitors, alone or in combination, in clinical trials for treatment of NF1-associated MPNST.

Among the 20 most up- and downregulated, several genes alter their expression in response to HDAC inhibitors such as the upregulated genes *TOP2A*, *BIRC5*, *HMGA2*, *CCNB1*, *CCNB2*, *TPX5* and *CDK1* [45–49], and the downregulated genes *SOX10* [50] and *GSN* [51]. As components of the epigenetic regulation system, HMGA2 and GSN are main targets of HDAC inhibitors, and suggest their utility as predictive treatment markers.

HMGA2 controls gene transcription directly *via* chromatin remodeling, or indirectly, by altering the binding affinity of regulators and nuclear proteins to DNA through protein-protein interactions. Involved in the control of fetal development, HMGA2 also has a central role in tumor growth and metastasis. HMGA2 protein levels rise acutely in malignancies [52], and its overexpression correlates with poor prognosis in colon, lung, pancreas, ovary and gastric cancers [53,54]. Besides use of *HMGA2* as a diagnostic/prognostic biomarker in NF progression to malignity, the reported silencing of HMGA2 with HDAC inhibitors justifies its use as a biomarker of treatment effectiveness of these drugs [55].

In addition to its structural role as one of the most abundant actin-binding proteins, the multifunctional regulator gelsolin GSN is involved in apoptosis and regulates processes related to pathological states such as amyloidosis, inflammation, Alzheimer's disease, cardiovascular diseases, cancer and aging [56]. In cancer, GSN has a dual effect as a promoter of cell growth and invasion [57] and as a tumor suppressor that inhibits metastasis. Its tumor suppressor effect is reported for most cancers, and it is downregulated in all of them [58]. GSN transcriptional repression is associated with epigenetic control through DNA methylation and histone deacetylation, and addition of HDAC inhibitors increases GSN expression [51], which supports its use as a biomarker for the effectiveness of MPNST treatment with HDAC inhibitors.

HMGA2 and GSN, as well as EZH2 and CBX7, respective members of PRC2 and PRC1 epigenetic repressor complexes, probably impaired during NF malignant transformation, are involved in the control of cell proliferation and metastatic phenotype by regulation of the tumor suppressor protein CDH1 and other markers of EMT, whose expression is reversed after treatment with HDAC inhibitors, particularly TSA [59]. HDAC inhibitor activity counteracts CBX7 silencing and EZH2 protein overexpression over the *CDH1* promoter, as discussed in Discussion D in S1 Appendix.

In addition to genes involved in epigenetic regulation as possible HDAC inhibitor targets, we found two other genes related to acetate metabolism and availability (*ASPA*, *ADH1B*) among the 20 most-underexpressed genes of the MPNST vs. NF signature. Supplementation with acetate precursors as coadjuvant chemotherapy to complement two metabolic pathways that involve ASPA and ADH1B in acetate synthesis is considered in Discussion E in S1 Appendix.

Cantharidin and tamoxifen were also retrieved by NFFinder and confirmed experimentally as effective drugs to inhibit MPNST cultured cell proliferation and survival. The protein phosphatase 2A inhibitor cantharidin was found by screening from a library of 472 small bioactive compound library [29] and shown to avoid growth of NF1-associated MPNST cultured cells, though additional studies should clarify the relevance of cantharidin *in vivo*.

Due to MPNST are not sex steroid hormone sensitive, the ER modulator tamoxifen inhibits MPNST cultured cell growth independently of ER [30,60]. Combination therapies of tamoxifen and trifluoperazine have shown to be effective on treatment of sporadic and NF1-associated MPNST, suggesting the quick repurposing of these drugs for clinical and prophylactic uses. Tamoxifen, which induces NF1-associated MPNST cell death mediated by autophagy in a K-Ras degradation dependent-process [61], might replace cloroquine and combine with HDAC inhibitors to induce apoptosis-oriented autophagy in “resistant” sporadic MPNST cells [28]. Tamoxifen and HDAC inhibitors might thus boost productive autophagy in a similar way as the combination of tamoxifen with panobinostat has been proposed for treatment of other solid tumors like hepatocellular carcinoma [62].

### Meta-analysis method to define the MPNST vs. NF gene signature

Various meta-analysis approaches have been developed to integrate data from independent studies. Due to their inherent difficulties, most meta-analyses restrict the inclusion of studies to a small number of different platforms or to a single platform. Integration of genomics data for rare diseases presents even greater challenges. Scanty heterogeneous data, common for rare diseases, might hide consistent information and patterns potentially present across studies. The meta-analysis method used here, based on previously described gene scores [23,26], identifies differentially expressed genes between two conditions, by integrating data from platforms of distinct size. Although we used a similar formula, our gene score differed slightly from that published in five aspects; a) scores were computed for genes as up- and downregulated, b) the logFC computed, based on the mean of expression ratios, was replaced by the logFC_m based on the median value of expression ratios, which increased score robustness, c) logFC_m values were normalized to the interval [0,1] for upregulated and [-1,0] for downregulated genes to avoid expression ratio bias among studies, d) the use of MAD (median absolute deviation) rather than standard error values to compute the penalty term due to expression deviation among replicates, which resulted in lower values for the penalty term and thus in more stringent scores, and e) stringency was reinforced by a third condition imposed for calculation of scores (B factor >0). Computation of the gene final score by the addition of each gene study score might favor genes represented in a higher number of platforms. The last filter step selected 10% of the genes on the complete list, which yielded the MPNST vs. NF signature; this is nonetheless a more balanced representation of the gene list for each of the starting studies, as it contains a larger proportion of genes common to various studies.

In addition to problems frequently encountered when comparing transcriptomes, integration of human and mouse data has drawbacks inherent to species-specific differences. As mouse models do not appear to mimic human neurodegenerative diseases at molecular level in every respect [27], we prioritized the human over the mouse transcriptome. Although doubts remained regarding the incorporation of mouse data in the meta-analysis, we included those data since robust genes expressed similarly in humans and mice could be candidates for preclinical studies. As the gene signature includes the behavior of each gene regarding the species and tissue type, it provides comprehensive information for the design of experiments.

The integrative gene signature associated to neurofibroma malignant evolution is the main contribution of this work. This signature constitutes the first step to generate working hypotheses concerning biomarkers of disease evolution and treatment effectiveness, as well as therapeutic drugs. Our *in silico* methodology used to define the signature and derive hypotheses for experimental purposes could be applied in the study of orphan diseases other than NF1.

## Conclusions

Here we used a meta-analysis method based on gene scores to define the gene signature associated to the transition of benign neurofibromas to MPNST. Signature components showing the highest/lowest scores are proposed as disease biomarkers. Given the greater diagnostic and prognostic robustness of biomarker panels compared to individual genes, we clustered functionally related genes from profiles that integrate additional NF1-related gene signatures, and established four panels derived from clusters 3, 17, 34 and cluster 25, which comprises genes involved in cell cycle progression. By further studying the epigenetic regulation of malignant transformation, we identified potential hypermethylated, silenced biomarkers, which links promoter methylation status to gene expression profiles.

The gene signature was used to search for drugs able to reverse malignant phenotype. Retrieved from NFFinder and previously tested for effectiveness, cantharidin, tamoxifen, TSA and other HDAC inhibitors have yielded promising effects as candidates for chemotherapy. We suggest *HMGA2* and *GSN* genes, two targets of HDAC inhibitors, as epigenetic biomarkers for testing the therapeutic effectiveness of HDAC inhibitors.

## Materials and methods

The detailed workflow of selection and pre-processing of microarrays used to obtain the MPNST vs. NF gene signature, translation to ENSEMBL gene names, and computation of gene scores and of effect size medians, and final filters are shown in S4 Fig. Where appropriate, tables are indicated for some of the main results generated (right). The meta-analysis PRISMA checklist is included as supporting information (S2 Appendix).

### Study selection

Microarray and DNA methylation studies were selected from GEO and ArrayExpress public databases using the key words NF1, MPNST, neurofibromatosis, and neurofibroma; we included high-throughput sequencing, microarray and methylation data. Additional selection criteria were 1) for microarray studies, only data from Affymetrix, Agilent, ABI and Illumina platforms were allowed, and 2) we only considered studies accepted for publication, with raw data from samples from cell cultures or nerve tumors.

### Microarray data preprocessing

Each microarray was preprocessed and evaluated independently using the R/BioConductor software environment [63]. R packages used in pre-processing steps are detailed in Materials and methods A in S1 Appendix. After grouping dNF and pNF samples as NF, and sporadic and NF1-associated as MPNST, the resulting expression set was filtered to discard features with FDR values >0.05 obtained by ANOVA. The expression set derived was used to assess appropriate grouping of samples from the independent studies by principal component analysis (PCA), as shown in S5 and S6 Figs. The final derived expression set was used to analyze gene differential expression.

### Probe name translation to human ENSEMBL and HUGO gene IDs and mapping in human chromosome arms

As integration of scores from different platforms required common identifiers, we translated probe names from each study to ENSEMBL human gene IDs according to Ensembl Archive release 82 (September 2015). To obtain a unique score for each gene, we selected the score of the probe with the greatest variation when the identifier was present more than once. This variation was calculated as the variance of normalized expression values of all samples from the two phenotypes compared. Mouse gene IDs were also translated to human homologous gene IDs. Human ENSEMBL gene IDs were associated with HUGO gene nomenclature IDs and mapped to human chromosomes and bands. Details of ENSEMBL gene ID association to human chromosome arms are shown in Materials and methods B in S1 Appendix.

### Gene scores across studies and final individual gene score in a two-phenotype comparison

Gene scores were also computed in the R/Bioconductor environment [63]; Materials and methods C in S1 Appendix details the formula and computation method used. In an example illustrating the factors involved in gene score computation, S13 Table shows values for the differentially expressed genes obtained by comparing MPNST vs. NF from the GSE66743 study. The final score across studies of a single species was calculated as the sum of standardized individual s_ij_ scores. Final human and mouse scores were weighted for human results. Genes exclusive to mouse were ignored and mouse data that differed from human data in the expression ratio sign were discarded. The human score was calculated by adding scores from human studies. Inclusion of murine data was limited to genes for which the sign for the final human and mouse scores were identical, and final human scores were not null.

### logFC and logFC_m of genes across studies

Gene logFC were calculated across the studies as the median value of logFC_ij_; logFC_ij_ values for each gene (i) in each individual study (j) were obtained in the phenotype comparison. Gene logFC_m were computed as the median value of logFC_m_ij_; we have defined logFC_m_ij_ as the log_2_ of the median value of all expression ratios between phenotypes. To avoid inconsistency, the same restriction imposed on the final score was used for logFC and logFC_m values. The signs for human median values of computed logFC/logFC_m were compared with those of mouse. If human and mouse signs were equal, the mouse logFC/ logFC_m was used to compute the final logFC/logFC_m. If the signs were different, mouse values were ignored and human values were used as the final logFC/logFC_m.

### Final filters to obtain the gene signature

To avoid inconsistency, the resulting list of genes with non-null score was screened to remove genes showing score signs opposite to those of logFC values (S4 Fig, third filter). To select genes with the highest score and median size effect values, a final filter was applied to the previous gene list (S4 Fig, fourth filter). This filter screened 10% of genes with the highest scores (genes with positive score) and 10% of genes with the lowest score (genes with negative score). In both cases, the absolute logFC value should be >0.99.

### Computation of bias in score values among studies: Bhattacharyya distance (BD) ratio

To determine the relative influence of individual experiments on final score values, we computed for each gene the BD between the observed distribution of score frequency and the expected discrete distribution if each study contributed equally to the final score. The lowest BD values thus correlated with similar contributions of individual studies to final score values, while the highest values indicated disparity in the contribution of individual studies. To compare BD without considering the number of studies available for each gene, we computed the BD ratio by dividing the BD by the maximum possible BD value considering the number of studies for each gene. The lowest and highest BD ratio values indicated the similar or dissimilar contribution of each study to each gene score, respectively. A BD ratio equal to zero was applied exceptionally to those genes for which only one study was available, to distinguish these genes from those for which there was more than one study, although only one showed a non-null score. Computation details are given in Materials and methods D in S1 Appendix.

### Statistical analyses

To determine the statistical significance of the different gene distribution frequencies in human chromosome regions, we calculated the accumulated probability of a binomial distribution P(X≥x) using the pbinom function from the R stats package; **** P ≤0.0001, *** 0.0001<P≤0.001, ** 0.001 <P≤0.01, * 0.01<P≤0.01. The expected frequency of gene distribution in human chromosome arms/bands was calculated considering the association of ENSEMBL gene IDs to human chromosome arms/bands. To assess correlation between values of two vectors, Pearson's product-moment correlation was calculated using the cor.test function from the R stats package.

### Gene and pathway functional enrichment

The over-representation test for GO terms from GO Ontology database release 2016_09_24 (The Gene Ontology Consortium, 2015) was performed through PANTHER v.10 (release 20160715; [64]) using ENSEMBL IDs as input, Homo sapiens (all genes in the database) as reference list, GO biological process, GO molecular function and GO cellular component complete as Annotation Data Set, and the Bonferroni correction for multiple testing (P <0.05). The EnrichNet tool [65] was used to inspect KEGG, BioCarta, Wiki Pathways and Reactome databases to search for pathways in which a differentially expressed group of genes is enriched compared to the whole human genome; ENSEMBL IDs were used as input. Only pathways with a significant XD-score or Fischer q-value <0.1 were considered. The search for opposite and similar gene signatures in CMap-DrugMatrix and GEO databases in NFFinder was performed as described [20].

### Gene clustering and visualization

Genes were grouped using SOTA [66] implemented in the analysis and visualization tool MultiexperimentViewer MeV v. 4.9 [67]. Parameters were fit by default, including distance (Pearson correlation), and Max. Cycles (100). Sample tree was carried out using hierarchical clustering to optimize sample leaf order. Pearson correlation was selected as the distance metric, and average linkage clustering as the linkage method.

### DNA methylation analysis

Methylation data were downloaded from GEO, and inspected and analyzed using the MEDIPS R package [68]. Linux command lines, R code based on Lienhard *et al*. [68], and details of the analysis are shown in Materials and methods E in S1 Appendix.

## Supporting information

S1 Table Gene score (s_ij_) and size effect values (logFC_ij_ and logFC_m_ij_) for the five studies included in MPNST vs. NF meta-analysis. (XLS)

S2 Table Gene signatures in the context of neurofibromatosis type 1. (ZIP)

S3 Table Results retrieved from NFFinder. (XLS)

S4 Table Functional characterization of the MPNST vs. NF gene signature. (XLS)

S5 Table Functional description of the 20 genes with the highest and lowest scores in the MPNST vs. NF signature. (PDF)

S6 Table Characterization of the 20 genes with the highest and lowest scores in the MPNST vs. NF signature. (PDF)

S7 Table Functional description of genes included in the over-represented chromosome regions. (XLS)

S8 Table List of 2209 genes included in signatures from cell cultures (NF vs. control, MPNST vs. control, MPNST vs. NF) and tumor tissue (MPNST vs. NF, MPNST vs. control, NF vs. control). (XLS)

S9 Table Functional description of genes included in SOTA clusters (biological process GO term enrichment). (XLS)

S10 Table MPNST vs. NF unfiltered list of genes potentially down-regulated by promoter hypermethylation. (XLS)

S11 Table Up-regulated genes from the MPNST vs. NF signature previously related with NF and/or MPNST. (PDF)

S12 Table Down-regulated genes from the MPNST vs. NF signature previously related with NF and/or MPNST. (PDF)

S13 Table Head and tail of GSE66743 data extracted from differential expression analysis between MPNST and NF phenotypes with other factors required to compute gene scores. (PDF)

S14 Table Functional characterization of additional gene signatures in the context of NF1. (XLS)

S1 Fig Comparison of gene score profiles of the integrative MPNST vs. NF gene signature and the five individual studies. **a.** Plot showing the percentage of score values for up- (score>0) and down- (score<0) regulated genes of the MPNST vs. NF unfiltered list. This list contains the score values of the 7064 unique ENSEMBL human genes sorted in x-axis from the highest to the lowest score value (Table A in S2 Table). Vertical red dot lines discriminate the first 336 up- and the last 243 downregulated genes with the highest absolute score values, included in the MPNST vs. NF gene signature. **b.** Plots with x and y axes equal to plot A showing the percentage of score values from non-null score genes represented in each of the five studies integrated in the MPNST vs. NF gene signature (E-MEXP-353, E-TABM-69, GSE41747 (human), GSE66743 and GSE41747 (mouse)). Unlike these five plots, that contain differentially expressed genes derived from the MPNST vs. NF comparison, the last plot (Control), as negative control, includes non-null score genes obtained in the NF vs. Control cell culture comparison from the GSE14038 accession.
(PDF)

S2 Fig Chromosome distribution of the MPNST vs. NF gene signature. The distribution was calculated from 4059 genes with positive score **(a)**, and from 3005 genes with negative score **(b)**. Bar diagrams compare the observed distribution of MPNST vs. NF gene percentage in the human chromosome arms (blue bars) with the expected distribution according to the human ENSEMBL database (red bars). Statistical significance of the gene signature over-represented chromosome arms is above the bars. Over-represented human chromosome bands in the MPNST vs. NF gene signature are shown below each chart. Their statistical significance is shown at the top right side of band names. (****) P(X≥x) < 0.0001, (***) 0.0001< P(X≥x) < 0.001, (**) 0.001< P(X≥x) < 0.01, (*) 0.01< P(X≥x) < 0.05.
(PDF)

S3 Fig Grey scale diagram that shows the percentage of genes from each gene signature included in the clustering process. Numbers over the gray scale diagram indicate the number of genes included in each cluster. The interval of color scale values is shown below the diagram. The right side of diagram details the number of genes in each cluster, the number of biological process GO terms over-represented in each cluster, and the summary of that GO term enrichment as functional characterization of clusters. A complete list of terms is shown in S9 Table.
(PDF)

S4 Fig Workflow of selection and pre-processing of microarrays selected to obtain the gene signature MPNST vs. NF, translation to ENSEMBL gene names, and computation of gene scores and median of logFC/logFC_m. Main outputs of some individual steps appear on the right. i: Each individual gene. j: Each individual study.
(PDF)

S5 Fig Principal components (PCA) plots obtained for each study included in MPNST vs. NF meta-analysis. The final number of probes (Table A in Results B in S1 Appendix) considered in the computation of PCA plots is shown. The legend of colored circles on the left shows sample phenotypes compared.
(PDF)

S6 Fig Principal components (PCA) plots obtained for other studies included in additional comparisons other than tumor tissue from MPNST vs. NF. The final number of probes (Table A in Results B in S1 Appendix) considered in the computation of PCA plots is shown. Colored circles on the left show the sample phenotypes compared in the analyses. PCA plot from GSE14038 (cell cultures) includes the samples from the previously described comparison MPNST vs. NF from GSE41747 (human tumor tissue) depicted in S5 Fig.
(PDF)

S1 Appendix Results A: Relationships between score values and additional attributes in the MPNST vs. NF gene signature.
Results B: Contribution of individual studies to the MPNST vs. NF gene signature.
Results C: Genes included in main functional pathways associated to the MPNST vs. NF signature.
Results D: Characterization of additional NF1-related gene signatures.
Results E: Homogeneity of gene profiles in each SOTA cluster.
Discussion A: Comparison between over-represented chromosome regions in the MPNST vs. NF gene signature and previously described MPNST aberrant chromosome modifications.
Discussion B: Expression profile differences between cultured cells and nerve tumors observed in *SOX9*, *SUZ12*, *EGFR*, *SPP1* and *BMP2* genes.
Discussion C: Panel of genes potentially silenced by hypermethylation of their CpG-island promoter region.
Discussion D: HDAC inhibitors counteract repression of CBX7 and over-expression of EZH2.
Discussion E: Supplementation with acetate precursors as coadjuvant chemotherapy.
Materials and methods A: Microarray data pre-processing.
Materials and methods B: Translation from probe names to human ENSEMBL gene IDs, HUGO IDs and mapping in human chromosome arms.
Materials and methods C: Scores of genes across studies and final score for each gene in a comparison between two phenotypes.
Materials and methods D: Computation of bias in score values among studies: Bhattacharya distance (BD) ratio.
Materials and methods E: DNA methylation analysis.
(PDF)

S2 Appendix Meta-analysis PRISMA checklist. (PDF)
