# Supplementary material for: Gene signature associated with benign neurofibroma transformation to malignant peripheral nerve sheath tumors
Source: PLoS One. 2017 May 24;12(5):e0178316. doi: 10.1371/journal.pone.0178316 (PMC5443557; doi:10.1371/journal.pone.0178316)
Supplement: S5 Table — (PDF) [file pone.0178316.s005.pdf]

S5 Table: Functional description of the 20 genes with the highest and lowest scores in the MPNST vs. NF signature.

ENSGENE[1]	hgnc symbol[2]	chrom name[3]	band	Description[4]
ENSG00000171848	RRM2	2	p25.1	Ribonucleotide Reductase Regulatory Subunit M2; It provides precursors for DNA synthesis.
ENSG00000131747	TOP2A	17	q21.2	Topoisomerase (DNA) II Alpha; It overcomes DNA topological problems resulting in DNA replication, transcription and repair
ENSG00000166803	KIAA0101	15	q22.31	Regulator of DNA repair during DNA replica□on; regulator of centrosome number.
ENSG00000089685	BIRC5	17	q25.3	Apoptosis Inhibitor Survivin; Essential for chromosome alignment and segregation during mitosis and cytokinesis.
ENSG00000137804	NUSAP1	15	q15.1	Nucleolar And Spindle Associated Protein 1; it promotes the organization of mitotic spindle microtubules around chromosomes.
ENSG00000185686	PRAME	22	q11.22	Preferentially Expressed Antigen In Melanoma; transcriptional repressor that prevents retinoic acid-induced cell proliferation arrest, differentiation and apoptosis.
ENSG00000149948	HMGA2	12	q14.3	High-Mobility Group (Non histone Chromosomal) Protein Isoform I-C; transcriptional regulator; it functions in cell cycle regulation and in chromosome condensation during meiotic G2/M transition.
ENSG00000117724	CENPF	1	q41	Centromere Protein F; required for kinetochore function and chromosome segregation in mitosis.
ENSG00000198901	PRC1	15	q26.1	Protein Regulator Of Cytokinesis 1; present at high levels during the S and G2/M phases of mitosis.
ENSG00000157456	CCNB2	15	q22.2	Cyclin B2; essential for the control of the cell cycle at mitotic G2/M transition.
ENSG00000128045	RASL11B	4	q12	RAS Like Family 11 Member B; small GTPase protein family with a high degree of similarity to RAS; involved in germ layer development and in tumors.
ENSG00000156076	WIF1	12	q14.3	WNT Inhibitory Factor 1; it may be involved in mesoderm segmentation.
ENSG00000134057	CCNB1	5	q13.2	Cyclin B1; essential for the control of the cell cycle at mitotic G2/M transition.
ENSG00000066279	ASPM	1	q31.3	Abnormal Spindle Microtubule Assembly; essential for normal mitotic spindle function in embryonic neuroblasts.
ENSG00000088325	TPX2	20	q11.21	Microtubule Nucleation Factor; required for normal assembly of mitotic spindles.
ENSG00000123975	CKS2	9	q22.2	Protein Kinase Regulatory Subunit; essential for the biological function of cyclin dependent kinases to which it binds.
ENSG00000143476	DTL	1	q32.3	Denticleless E3 Ubiquitin Protein Ligase Homolog; required for cell cycle control, DNA damage response and translesion DNA synthesis.
ENSG00000170312	CDK1	10	q21.2	Cyclin-Dependent Kinase 1; it controls cell cycle by modulating the centrosome cycle and mitotic onset.
ENSG00000007062	PROM1	4	p15.32	Prominin 1; pentaspan transmembrane glycoprotein; it maintains stem cell properties by suppressing differentiation.
ENSG00000115163	CENPA	2	p23.3	Centromere Protein A; required for recruitment and assembly of kinetochore proteins, mitotic progression and chromosome segregation.
ENSG00000021300	PLEKHB1	11	q13.4	Pleckstrin Homology Domain Containing B1; required for proper localization of retinogeniculate projections but not for eye-specific segregation.
ENSG00000109846	CRYAB	11	q23.1	Crystallin Alpha B; chaperone that prevents aggregation of proteins under stress conditions; it may contribute to the transparency and refractive index of the lens.
ENSG00000100146	SOX10	22	q13.1	SRY-Box 10; regulator of neural crest and peripheral nervous system development.
ENSG00000100307	CBX7	22	q13.1	Component of a Polycomb PRC1-like complex required to maintain the transcriptionally repressive state of many genes, including Hox genes, throughout development; it maintains the repression of CDKN2A.
ENSG00000149218	ENDOD1	11	q21	Endonuclease Domain Containing 1; androgen-regulated gene, down-regulated in prostate cancer.
ENSG00000197766	CFD	19	p13.3	Complement Factor D (Adipsin); chymotrypsin that catalyzes the cleavage of factor B, the rate-limiting step of the alternative pathway of complement activation.
ENSG00000148180	GSN	9	q33.2	Gelsolin; calcium-regulated, actin-modulating protein that binds to actin monomers or filaments.
ENSG00000134121	CHL1	3	p26.3	Cell Adhesion Molecule L1 Like; it plays a role in nervous system development and in synaptic plasticity.
ENSG00000172005	MAL	2	q11.1	Mal T-Cell Differentiation Protein; highly hydrophobic membrane proteolipid involved in myelin biogenesis and/or myelin function.
ENSG00000174944	P2RY14	3	q25.1	Purinergic Receptor P2Y14; P2Y purinergic receptor for UDP-glucose and other UDP-sugars coupled to G-proteins.
ENSG00000108381	ASPA	17	p13.2	Aspartoacylase; it catalyzes the conversion of N-acetyl_L-aspartic acid (NAA) to aspartate and acetate to maintain brain white matter.
ENSG00000168477	TNXB	6	p21.32	Tenascin XB; extracellular matrix glycoprotein with anti-adhesive effect.
ENSG00000127951	FGL2	7	q11.23	Fibrinogen Like 2; it may play a role in physiologic lymphocyte functions at mucosal sites.
ENSG00000196616	ADH1B	4	q23	Alcohol Dehydrogenase 1B (Class I), Beta Polypeptide; it plays a major role in ethanol catabolism.
ENSG00000071991	CDH19	18	q22.1	Cadherin 19; calcium-dependent cell adhesion protein.
ENSG00000147588	PMP2	8	q21.13	Peripheral Myelin Protein 2; it may play a role in lipid transport protein in Schwann cells.
ENSG00000171819	ANGPTL7	1	p36.22	Angiopoietin Like 7.
ENSG00000148671	ADIRF	10	q23.2	Adipogenesis Regulatory Factor; it plays a role in fat cell development; promotes adipogenic differentiation and stimulates transcription initiation of master adipogenesis factors.
ENSG00000160307	S100B	21	q22.3	S100 Calcium Binding Protein B; calcium-binding protein involved in cell cycle progression and differentiation.
ENSG00000107317	PTGDS	9	q34.3	Prostaglandin D2 Synthase; its product, PGD2, functions as a neuromodulator as well as a trophic factor in the central nervous system.

[1]ENSEMBL gene ID.

[2]Gene symbol from HUGO Gene Nomenclature Committee.

[3]Human chromosome name.

[4]Description according Human Gene Database GeneCards.
