# Supplementary material for: Gene signature associated with benign neurofibroma transformation to malignant peripheral nerve sheath tumors
Source: PLoS One. 2017 May 24;12(5):e0178316. doi: 10.1371/journal.pone.0178316 (PMC5443557; doi:10.1371/journal.pone.0178316)
Supplement: S6 Table — (PDF) [file pone.0178316.s006.pdf]

S6 Table: Characterization of the 20 genes with the highest and lowest scores in the MPNST vs. NF signature.

hgnc symbol[1]	Involved in	Localization	TF[2]	Up-regulated in cancer	Down-regulated in cancer	Prognostic value
RRM2	Cell cycle	Cytosol; Nucleus		[1]	[2]	
TOP2A	Cell cycle: controls and alters the topologic states of DNA during transcription.	Nucleus		[3,4]		[5,6]
KIAA0101	Cell cycle: regulator of DNA repair during DNA replication; regulator of centrosome number.	Nucleus		[6,7]	[8]	
BIRC5	Cell cycle: negative regulatory protein that prevents apoptotic cell death; regulator of the localization of chromosome passage protein complex (CPC) which is essential for chromosome alignment and segregation during mitosis and cytokinesis	Cytoskeleton; Cytosol; Nucleus		[4,9]		[6,10]
NUSAP1	Cell cycle: stabilizes microtubules; controls centrosome numbers.	Nucleus		[12]		[12,13]
PRAME	Cell cycle: repressor of retinoic acid receptor; prevents retinoic acid-induced cell proliferation arrest, differentiation and apoptosis.	Nucleus		[14]		[15]
HMGA2	Cell cycle regulation.	Nucleus	[16]	[17]		[18]
CENPF	Cell cycle: involved in the regulation of DNA synthesis and hence cell cycle progression.	Cytoskeleton; Cytosol; Nucleus		[19]		[19]
PRC1	Cell cycle: Regulator of cytokinesis.	Cytoskeleton; Cytosol; Nucleus; Plasma Membrane		[20]		[21]
CCNB2	Cell cycle: control the cell cycle at the G2/M (mitosis) transition.	Cytoskeleton; Cytosol; Nucleus		[22]		[23]
RASL11B						
WIF1	Cell cycle: binds to WNT proteins and inhibits their activities.	Extracellular space		[24]	[25]	[26]
CCNB1	Cell cycle: controls the cell cycle at the G2/M (mitosis) transition.	Cytoskeleton; Cytosol; Nucleus		[27,28]	[29]	[28]
ASPM	Cell cycle: role in mitotic spindle regulation and coordination of mitotic processes	Cytoskeleton; Nucleus		[30]		[31]
TPX2	Cell cycle	Cytoskeleton; Nucleus		[32]		[33]
CKS2	Cell cycle: binds to the catalytic subunit of the cyclin dependent kinases and is essential for their biological function.			[34]		[35]
DTL	Cell cycle	Cytoskeleton; Nucleus		[36]		[37]
CDK1	Cell cycle: key role in the control of the eukaryotic cell cycle by modulating the centrosome cycle as well as mitotic onset.	Cytoskeleton; Cytosol; Nucleus; Extracellular space; Mitochondrion		[38]		[38]
PROM1	Cell cycle: key regulator of disk morphogenesis (retina)	Endoplasmic Reticulum; Extracellular space; Plasma membrane		[39]		[39,40]
CENPA	Cell cycle	Nucleus		[41]		[42]
PLEKHB1	Signal transduction (nervous system)			[43]		[44]
CRYAB	Protein conformation	Cytosol; Extracellular space; Nucleus		[45]	[46]	[45]
SOX10	Development (nervous system)	Nucleus	[16]	[47]	[4]	[47]
CBX7	Cell cycle	Nucleus			[48]	[49]
ENDOD1	Inflammatory response (brain)				[50]	
CFD	Immune response	Extracellular space		[51]	[52,53]	
GSN	Tumor suppression	Cytoskeleton; Cytosol; Extracellular space; Plasma membrane		[54,55]	[54]	[55]
CHL1	Neural signal transduction	Extracellular space			[56]	
MAL	Cell trafficking	Endoplasmic Reticulum; Plasma membrane			[57]	[58]
P2RY14	Inflammation mediation	Plasma membrane		[59]	[52]	
ASPA	Synthesis of acetate	Cytosol; Extracellular space			[60]	[61]
TNXB	ECM integrity	Extracellular space		[62]	[63]	[64]
FGL2	Immune response	Extracellular space		[65]	[66]	[67]
ADH1B	Catabolism of alcohol	Cytosol		[68]	[69]	[68]
CDH19	Cell-cell junction; cell integrity	Plasma membrane		[70]	[71]	[70]
PMP2	Myelin formation	Extracellular space			[72]	
ANGPTL7	ECM formation	Extracellular space		[73]	[74]	[74]
ADIRF	Lipid metabolism: promotes adipogenic differentiation and stimulates transcription initiation of master adipogenesis factors.				[75]	[75]
S100B	Signal transduction; immune response	Extracellular space; Nucleus	[76]	[77]	[4]	[77]
PTGDS	Signal transduction	Endoplasmic Reticulum; Extracellular space			[78]	[78]

[1]Gene symbol from HUGO Gene Nomenclature Committee.

[2]Transcription Factor.

1. Goss KL, Gordon DJ. Gene expression signature based screening identifies ribonucleotide reductase as a candidate therapeutic target in Ewing sarcoma. Oncotarget. 2016;5:1–17.

2. Aird KM, Zhang G, Li H, Tu Z, Bitler BG, Garipov A, et al. Suppression of Nucleotide Metabolism Underlies the Establishment and Maintenance of Oncogene-Induced Senescence. Cell Rep. 2013;3:1252–65.

3. Wong N, Yeo W, Wong W-L, Wong NL-Y, Chan KY-Y, Mo FK-F, et al. TOP2A overexpression in hepatocellular carcinoma correlates with early age onset, shorter patients survival and chemoresistance. Int J Cancer. 124:644–52.

4. Lévy P, Vidaud D, Leroy K, Laurendeau I, Wechsler J, Bolasco G, et al. Molecular profiling of malignant peripheral nerve sheath tumors associated with neurofibromatosis type 1, based on large-scale real-time RT-PCR. Mol Cancer. 2004;3:20.

5. Bai Y, Li L-D, Li J, Lu X. Targeting of topoisomerases for prognosis and drug resistance in ovarian cancer. J Ovarian Res. 2016;9:35.

6. Kolberg M, Høland M, Lind GE, Ågesen TH, Skotheim RI, Sundby Hall K, et al. Protein expression of BIRC5, TK1, and TOP2A in malignant peripheral nerve sheath tumours - A prognostic test after surgical resection. Mol Oncol. 2015;9:1129–39.

7. Jain M, Zhang L, Patterson EE, Kebebew E. KIAA0101 Is Overexpressed, and Promotes Growth and Invasion in Adrenal Cancer. PLoS ONE. 2011;6:e26866.

8. Kato T, Daigo Y, Aragaki M, Ishikawa K, Sato M, Kaji M. Overexpression of KIAA0101 predicts poor prognosis in primary lung cancer patients. Lung Cancer. 2012;75:110–8.

9. Fukuda S, Pelus LM. Survivin, a cancer target with an emerging role in normal adult tissues. Mol Cancer Ther. 2006;5:1087–98.

10. Stauber RH, Mann W, Knauer SK. Nuclear and cytoplasmic survivin: molecular mechanism, prognostic, and therapeutic potential. Cancer Res.2007;67:5999–6002.

11. Kotian S, Banerjee T, Lockhart A, Huang K, Catalyurek U V, Parvin JD. NUSAP1 influences the DNA damage response by controlling BRCA1 protein levels. Cancer Biol Ther.2014;15:533–43.

12. Fang L, Zhang M, Chen L, Xiong H, Ge Y, Lu W, et al. Downregulation of nucleolar and spindle-associated protein 1 expression suppresses cell migration, proliferation and invasion in renal cell carcinoma. Oncol Rep. 2016;36:1506–16.

13. Chen L, Yang L, Qiao F, Hu X, Li S, Yao L, et al. High Levels of Nucleolar Spindle-Associated Protein and Reduced Levels of BRCA1 Expression Predict Poor Prognosis in Triple-Nega□ve Breast Cancer. PLoS One. 2015;10:e0140572.

14. Hermes N, Kewitz S, Staege MS. Preferentially Expressed Antigen in Melanoma (PRAME) and the PRAME Family of Leucine-Rich Repeat Proteins. Curr Cancer Drug Targets. 2016;16:400–14.

15. Field MG, Decatur CL, Kurtenbach S, Gezgin G, van der Velden PA, Jager MJ, et al. PRAME as an Independent Biomarker for Metastasis in Uveal Melanoma. Clin Cancer Res. 2016;22:1234–42.

16. Vaquerizas JM, Kummerfeld SK, Teichmann SA, Luscombe NM. A census of human transcription factors: function, expression and evolution. Nat Rev Genet. 2009;10:252–63.

17. Wei L, Liu X, Zhang W, Wei Y, Li Y, Zhang Q, et al. Overexpression and oncogenic function of HMGA2 in endometrial serous carcinogenesis. Am J Cancer Res. 2016;6:249–59.

18. Lee J, Ha S, Jung C-K, Lee HH. High-mobility-group A2 overexpression provokes a poor prognosis of gastric cancer through the epithelial-mesenchymal transition. Int J Oncol. 2015;46:2431–8.

19. Zhuo Y-J, Xi M, Wan Y-P, Hua W, Liu Y-L, Wan S, et al. Enhanced expression of centromere protein F predicts clinical progression and prognosis in patients with prostate cancer. Int J Mol Med. 2015;35:966–72.

20. Shimo A, Nishidate T, Ohta T, Fukuda M, Nakamura Y, Katagiri T. Elevated expression of protein regulator of cytokinesis 1, involved in the growth of breast cancer cells. Cancer Sci. 2007;98:174–81.

21. Chen J, Rajasekaran M, Xia H, Zhang X, Kong SN, Sekar K, et al. The microtubule-associated protein PRC1 promotes early recurrence of hepatocellular carcinoma in association with the Wnt/β-catenin signalling pathway. Gut. 2016;65:1522–34.

22. Lei C, Wang W, Zhu Y, Fang W, Tan W. The decrease of cyclin B2 expression inhibits invasion and metastasis of bladder cancer. Urol Oncol. 2016;34:237.e1-10.

23. Takashima S, Saito H, Takahashi N, Imai K, Kudo S, Atari M, et al. Strong expression of cyclin B2 mRNA correlates with a poor prognosis in patients with non-small cell lung cancer. Tumour Biol. 2014;35:4257–65.

24. Boerboom D, White LD, Dalle S, Courty J, Richards JS. Dominant-stable beta-catenin expression causes cell fate alterations and Wnt signaling antagonist expression in a murine granulosa cell tumor model. Cancer Res. 2006;66:1964–73.

25. Vassallo I, Zinn P, Lai M, Rajakannu P, Hamou M-F, Hegi ME. WIF1 re-expression in glioblastoma inhibits migration through attenuation of non-canonical WNT signaling by downregulating the lncRNA MALAT1. Oncogene. 2016;35:12–21.

26. Paluszczak J, Sarbak J, Kostrzewska-Poczekaj M, Kiwerska K, Jarmuż-Szymczak M, Grenman R, et al. The negative regulators of Wnt pathway-DACH1, DKK1, and WIF1 are methylated in oral and oropharyngeal cancer and WIF1 methylation predicts shorter survival. Tumour Biol. 2015;36:2855–61.

27. Gustafsson N, Zhao C, Gustafsson J-A, Dahlman-Wright K. RBCK1 drives breast cancer cell proliferation by promoting transcription of estrogen receptor alpha and cyclin B1. Cancer Res. 2010;70:1265–74.

28. Nakayama Y, Yamaguchi N. Role of cyclin B1 levels in DNA damage and DNA damage-induced senescence. Int Rev Cell Mol Biol. 2013;305:303–37.

29. Fang Y, Liang X, Jiang W, Li J, Xu J, Cai X. Cyclin b1 suppresses colorectal cancer invasion and metastasis by regulating ecadherin. PLoS One. 2015;10:e0126875.

30. Wang W-Y, Hsu C-C, Wang T-Y, Li C-R, Hou Y-C, Chu J-M, et al. A gene expression signature of epithelial tubulogenesis and a role for ASPM in pancreatic tumor progression. Gastroenterology. 2013;145:1110–20.

31. Alsiary R, Brüning-Richardson A, Bond J, Morrison EE, Wilkinson N, Bell SM. Deregula□on of microcephalin and ASPM expression are correlated with epithelial ovarian cancer progression. PLoS One. 2014;9:e97059.

32. Garrido G, Vernos I. Non-centrosomal TPX2-Dependent Regulation of the Aurora A Kinase: Functional Implications for Healthy and Pathological Cell Division. Front Oncol. 2016;6:88.

33. Neumayer G, Belzil C, Gruss OJ, Nguyen MD. TPX2: of spindle assembly, DNA damage response, and cancer. Cell Mol Life Sci. 2014;71:3027–47.

34. You H, Lin H, Zhang Z. CKS2 in human cancers: Clinical roles and current perspectives (Review). Mol Clin Oncol. 2015;3:459–63.

35. Yu M-H, Luo Y, Qin S-L, Wang Z-S, Mu Y-F, Zhong M. Up-regulated CKS2 promotes tumor progression and predicts a poor prognosis in human colorectal cancer. Am J Cancer Res. 2015;5:2708–18.

36. Zhu L, Xiao F, Yu Y, Wang H, Fang M, Yang Y, et al. KSP inhibitor SB743921 inhibits growth and induces apoptosis of breast cancer cells by regulating p53, Bcl-2, and DTL. An□cancer Drugs. 2016;27:863–72.

37. Benamar M, Guessous F, Du K, Corbett P, Obeid J, Gioeli D, et al. Inactivation of the CRL4-CDT2-SET8/p21 ubiquitylation and degradation axis underlies the therapeutic efficacy of pevonedistat in melanoma. EBioMedicine. 2016;10:85-100.

38. Wookyeom Y, Hanbyoul C, Ha-Yeon S, Joon-Yong C, Kang ES, Eun-Ju L, et al. Accumulation of cytoplasmic Cdk1 is associated with cancer growth and survival rate in epithelial ovarian cancer. Oncotarget. 2016;7:49481-49497.

39. Raso A, Mascelli S, Biassoni R, Nozza P, Kool M, Pistorio A, et al. High levels of PROM1 (CD133) transcript are a potential predictor of poor prognosis in medulloblastoma. Neuro Oncol. 2011;13:500–8.

40. Qiu Z-X, Zhao S, Mo X-M, Li W-M. Overexpression of PROM1 (CD133) confers poor prognosis in non-small cell lung cancer. Int J Clin Exp Pathol. 2015;8:6589–95.

41. Hi□ E, Bakheet T, Al-Souhibani N, Moghrabi W, Al-Yahya S, Al-Ghamdi M, et al. Systematic Analysis of AU-Rich Element Expression in Cancer Reveals Common Functional Clusters Regulated by Key RNA-Binding Proteins. Cancer Res. 2016;76:4068-4080.

42. Qiu J-J, Guo J-J, Lv T-J, Jin H-Y, Ding J-X, Feng W-W, et al. Prognostic value of centromere protein-A expression in patients with epithelial ovarian cancer. Tumour Biol. 2013;34:2971–5.

43. Johansson FK, Göransson H, Westermark B. Expression analysis of genes involved in brain tumor progression driven by retroviral insertional mutagenesis in mice. Oncogene. 2005;24:3896–905.

44. Colin C, Baeza N, Bartoli C, Fina F, Eudes N, Nanni I, et al. Identification of genes differentially expressed in glioblastoma versus pilocytic astrocytoma using Suppression Subtractive Hybridization. Oncogene. 2006;25:2818–26.

45. Shi C, He Z, Hou N, Ni Y, Xiong L, Chen P. Alpha B-crystallin correlates with poor survival in colorectal cancer. Int J Clin Exp Pathol. 2014;7:6056–63.

46. Al□ntas DM, Allioli N, Decaussin M, de Bernard S, Ruffion A, Samarut J, et al. Differentially Expressed Androgen-Regulated Genes in Androgen-Sensitive Tissues Reveal Potential Biomarkers of Early Prostate Cancer. PLoS One. 2013;8:e66278.

47. Zhao Y, Liu Z-G, Tang J, Zou R-F, Chen X-Y, Jiang G-M, et al. High expression of Sox10 correlates with tumor aggressiveness and poor prognosis in human nasopharyngeal carcinoma. OncoTargets Ther. 2016;9:1671–7.

48. Meseure D, Vacher S, Alsibai KD, Nicolas A, Chemlali W, Caly M, et al. Expression of ANRIL-Polycomb Complexes-CDKN2A/B/ARF Genes in Breast Tumors:

Identification of a Two-Gene (EZH2/CBX7) Signature with Independent Prognostic Value. Mol Cancer Res. 2016;14:623–33.

49. Pallante P, Sepe R, Puca F, Fusco A. High mobility group a proteins as tumor markers. Front Med (Lausanne). 2015;2:15.

50. Marques RB, Dits NF, Erkens-Schulze S, van Ijcken WFJ, van Weerden WM, Jenster G. Modula□on of androgen receptor signaling in hormonal therapy-resistant prostate cancer cell lines. PLoS One. 2011;6:e23144.

51. Hamilton G, Rath B, Klameth L, Hochmair MJ. Small cell lung cancer: Recruitment of macrophages by circulating tumor cells. Oncoimmunology. 2016;5:e1093277.

52. Uhlén M, Fagerberg L, Hallström BM, Lindskog C, Oksvold P, Mardinoglu A, et al. Proteomics. Tissue-based map of the human proteome. Science. 2015;347:1260419.

53. Mangano A, Messina L, Birgillito S, Stivala F, Bernardini A. Complement and its fractions (C3-C4) pa□ern in subjects with neoplasia. J Immunopharmacol. 1984;6:147–62.

54. Deng B, Fang J, Zhang X, Qu L, Cao Z, Wang B. Role of gelsolin in cell proliferation and invasion of human hepatocellular carcinoma cells. Gene. 2015;571:292–7.

55. Ma X, Sun W, Shen J, Hua Y, Yin F, Sun M, et al. Gelsolin promotes cell growth and invasion through the upregulation of p-AKT and p-P38 pathway in osteosarcoma. Tumour Biol. 2016;37:7165–74.

56. Chen J, Fu L, Zhang L-Y, Kwong DL, Yan L, Guan X-Y. Tumor suppressor genes on frequently deleted chromosome 3p in nasopharyngeal carcinoma. Chin J Cancer. 2012;31:215–22.

57. Ma R, Xu YE, Wang M, Peng W. Suppression of MAL gene expression is associated with colorectal cancer metastasis. Oncol Lett. 2015;10:957–61.

58. Iwasaki T, Matsushita M, Nonaka D, Nagata K, Kato M, Kuwamoto S, et al. Lower expression of CADM1 and higher expression of MAL in Merkel cell carcinomas are

associated with Merkel cell polyomavirus infection and better prognosis. Human Pathol. 2016;48:1–8.

59. Wu X, Zang W, Cui S, Wang M. Bioinformatics analysis of two microarray gene-expression data sets to select lung adenocarcinoma marker genes. Eur Rev Med Pharmacol Sci. 2012;16:1582–7.

60. Long P, Moffe□ J, Namboodiri A. N-acetylaspartate (NAA) and N-acetylaspartylglutamate (NAAG) promote growth and inhibit differentiation of glioma stem-like cells. J Biol Chem. 2013; 288:26188-200.

61. Long PM, Stradecki HM, Minturn JE, Wesley U V, Jaworski DM. Differential aminoacylase expression in neuroblastoma. Int J Cancer. 2011;129:1322–30.

62. Kim Y-S, Hwan J Do, Bae S, Bae D-H, Shick WA. Identification of differentially expressed genes using an annealing control primer system in stage III serous ovarian carcinoma. BMC Cancer. 2010;10:576.

63. Lévy P, Ripoche H, Laurendeau I, Lazar V, Ortonne N, Parfait B, et al. Microarray-based identification of Tenascin C and Tenascin XB, genes possibly involved in tumorigenesis associated with neurofibromatosis type 1. Clin Cancer Res. 2007;13:398–407.

64. Yuan Y, Nymoen DA, Stavnes HT, Rosnes AK, Bjørang O, Wu C, et al. Tenascin-X is a novel diagnostic marker of malignant mesothelioma. Am J Surg Pathol. 2009;33:1673–82.

65. Liu Y, Xu L, Zeng Q, Wang J, Wang M, Xi D, et al. Downregulation of FGL2/prothrombinase delays HCCLM6 xenograft tumour growth and decreases tumour angiogenesis. Liver Int. 2012;32:1585–95.

66. Kohno T, Moriuchi R, Katamine S, Yamada Y, Tomonaga M, Matsuyama T. Identification of genes associated with the progression of adult T cell leukemia (ATL). Jpn J Cancer Res. 2000;91:1103–10.

67. Yan J, Kong L-Y, Hu J, Gabrusiewicz K, Dibra D, Xia X, et al. FGL2 as a Multimodality Regulator of Tumor-Mediated Immune Suppression and Therapeutic Target in Gliomas. J Natl Cancer Inst. 2015;107.

68. Tucker SL, Gharpure K, Herbrich SM, Unruh AK, Nick AM, Crane EK, et al. Molecular biomarkers of residual disease after surgical debulking of high-grade serous ovarian cancer. Clin Cancer Res. 2014;20:3280–8.

69. Ye H, Yu T, Temam S, Ziober BL, Wang J, Schwartz JL, et al. Transcriptomic dissection of tongue squamous cell carcinoma. BMC Genomics. 2008;9:69.

70. Zorniak M, Clark PA, Kuo JS. Myelin-forming cell-specific cadherin-19 is a marker for minimally infiltrative glioblastoma stem-like cells. J Neurosurg. 2015;122:69–77.

71. Bujko M, Kober P, Mikula M, Ligaj M, Ostrowski J, Siedlecki JA. Expression changes of cell-cell adhesion-related genes in colorectal tumors. Oncol Lett. 2015;9:2463–70.

72. Cai Y, Zhong X, Wang Y, Yang J. Screening feature genes of astrocytoma using a combined method of microarray gene expression profiling and bioinformatics analysis. Int J Clin Exp Med. 2015;8:18004–12.

73. Parri M, Pietrovito L, Grandi A, Campagnoli S, De Camilli E, Bianchini F, et al. Angiopoietin-like 7, a novel pro-angiogenetic factor over-expressed in cancer. Angiogenesis. 2014;17:881–96.

74. Lim SY, Gordon-Weeks A, Allen D, Kersemans V, Beech J, Smart S, et al. Cd11b(+) myeloid cells support hepatic metastasis through down-regulation of angiopoietin-like 7 in cancer cells. Hepatology. 2015;62:521–33.

75. Øverbye A, Skotland T, Koehler CJ, Thiede B, Seierstad T, Berge V, et al. Identification of prostate cancer biomarkers in urinary exosomes. Oncotarget. 2015;6:30357–76.

76. Sorci G, Giovannini G, Riuzzi F, Bonifazi P, Zelante T, Zagarella S, et al. The Danger Signal S100B Integrates Pathogen– and Danger–Sensing Pathways to Restrain Inflammation. PLoS Pathog. 2011;7:114–9.

77. Holla FK, Postma TJ, Blankenstein MA, van Mierlo TJM, Vos MJ, Sizoo EM, et al. Prognostic value of the S100B protein in newly diagnosed and recurrent glioma patients: a serial analysis. J Neurooncol. 2016;1–8.

78. Thompson VC, Day TK, Bianco-Miotto T, Selth LA, Han G, Thomas M, et al. A gene signature identified using a mouse model of androgen receptor-dependent prostate cancer predicts biochemical relapse in human disease. Int J Cancer. 2012;131:662–72.
