# Supplementary material for: Gene signature associated with benign neurofibroma transformation to malignant peripheral nerve sheath tumors
Source: PLoS One. 2017 May 24;12(5):e0178316. doi: 10.1371/journal.pone.0178316 (PMC5443557; doi:10.1371/journal.pone.0178316)
Supplement: S11 Table — (PDF) [file pone.0178316.s011.pdf]

S11 Table: Up-regulated genes from the MPNST vs. NF signature previously related with NF and/or MPNST.

Rank	ENSGENE	hgnc_symbol	chrom_name	band	Relationship with NF or MPNST	Reference
2	ENSG00000131747	TOP2A	17	q21.2	Up-regulated gene in MPNST vs. pNF.<br>Increased copy number when it appeared overexpressed in human malignant brain tumors.<br>Up-regulated in MPNST vs. benign NF; poor prognosis marker.	[1]<br>[2]<br>[3]
4	ENSG00000089685	BIRC5	17	q25.3	Up-regulated gene in MPNST vs. pNF.<br>Increased copy number when it appeared overexpressed in human malignant brain tumors.<br>Highly expressed in human MPNSTs at both mRNA and protein level in both nucleus and cytoplasm; viable target for therapeutic treatment .<br>Up-regulated in MPNST vs. benign NF; poor prognosis marker.	[1]<br>[2]<br>[4]<br>[3]
7	ENSG00000149948	HMGA2	12	q14.3	High expression in MPNST; marker to differentiate MPNST from synovial sarcoma.	[5]
15	ENSG00000088325	TPX2	20	q11.21	Regulator of AURKA, therapeutic target for MPNST.	[6]
32	ENSG00000148773	MKI67	10	q26.2	Proliferation index of neurofibromas in NF1 patients.<br>Up-regulated gene in MPNST vs. pNF.	[7]<br>[1]
39	ENSG00000167900	TK1	17	q25.3	Up-regulated in MPNST vs. benign NF; poor prognosis marker.	[3]
42	ENSG00000168078	PBK	8	p21.1	Kinase overexpressed (mRNA and protein) with malignant transformation.	[8]
46	ENSG00000111206	FOXM1	12	p13.33	Amplification and increased protein expression; marker for poor survival in MPNST patients.	[9]
50	ENSG00000156970	BUB1B	15	q15.1	Kinase overexpressed (mRNA and protein) in malignant transformation.	[8]
70	ENSG00000134013	LOXL2	8	p21.3	Candidate target for amplification in its respective genomic region.<br>Significant amplification aberration in MPNSTs.	[10]<br>[11]
74	ENSG00000072571	HMMR	5	q34	Up-regulated gene in MPNST vs. pNF.<br>Regulator of AURKA; silencing of HMMR sensitizes MPNST cells against AURKA inhibition.<br>Significant deletion aberration in MPNSTs.	[1]<br>[6]<br>[11]
77	ENSG00000117650	NEK2	1	q32.3	Kinase overexpressed (mRNA and protein) in malignant transformation.	[8]
104	ENSG00000112319	EYA4	6	q23.2	Overexpressed in MPNST cell lines; its inhibition reduced cell adhesion and migration inducing cellular necrosis.<br>Genomic aberration in MPNSTs.	[12]<br>[11]

114	ENSG00000106462	EZH2	7	q36.1	Its inhibition blocks MPNST cell growth and induced apoptosis.	[13]
138	ENSG00000118785	SPP1	4	q22.1	Overexpressed in MPNST vs. NF.	[14]
140	ENSG00000104415	WISP1	8	q24.22	Significantly up-regulated in MPNSTs.	[15]
158	ENSG00000137745	MMP13	11	q22.2	Up-regulated gene in MPNST vs. pNF.<br>MPNST-specific DNA deletion; copy number changes observed in 43% of malignant tumors.<br>Significant deletion aberration in MPNSTs.	[1]<br>[16]<br>[11]
166	ENSG00000104313	EYA1	8	q13.3	Overexpressed in MPNSTs; its inhibition induced MPNST necrosis.	[12]
181	ENSG00000126778	SIX1	14	q23.1	Overexpressed in MPNSTs.	[12]
201	ENSG00000073756	PTGS2	1	q31.1	Overexpression in MPNSTs associated with poor outcome.	[17]
211	ENSG00000100985	MMP9	20	q13.12	Up-regulated gene in MPNST vs. pNF.<br>Up-regulated gene in pNF vs. dNF; it discriminates pNFs prone to malignant transformation.	[1]<br>[18]
216	ENSG00000087586	AURKA	20	q13.2	Potential MPNST therapeutic target.<br>Silenced through an AURKA therapeutic inhibitor.	[19]<br>[6]
236	ENSG00000122691	TWIST1	7	p21.1	Overexpressed neural crest stem cell marker in MPNST cell lines vs. normal Schwann cells.	[20]
238	ENSG00000123374	CDK2	12	q13.2	Sporadic and NF1-associated MPNSTs expressed normal sized protein.	[21]
260	ENSG00000146674	IGFBP3	7	p12.3	IGFBP3 serum levels low in a NF1 patient with growth hormone insensitivity.	[22]
281	ENSG00000132646	PCNA	20	p12.3	It potentially discriminates benign peripheral nerve sheath tumors from MPNSTs.	[23]

1. Lévy P, Vidaud D, Leroy K, Laurendeau I, Wechsler J, Bolasco G, et al. Molecular profiling of malignant peripheral nerve sheath tumors associated with neurofibromatosis type 1, based on large-scale real-time RT-PCR. Mol Cancer. 2004;3:20.

2. Storlazzi C, Brekke H, Mandahl N, Brosjö O, Smeland S, Lothe R, et al. Identification of a novel amplicon at distal 17q containing the *BIRC5/SURVIVIN* gene in malignant peripheral nerve sheath tumours. J Pathol. 2006;209:492–500.

3. Kolberg M, Høland M, Lind GE, Ågesen TH, Skotheim RI, Sundby Hall K, et al. Protein expression of BIRC5, TK1, and TOP2A in malignant peripheral nerve sheath tumours - A prognostic test after surgical resection. Mol Oncol. 2015;9:1129–39.

4. Ghadimi MP, Young ED, Belousov R, Zhang Y, Lopez G, Lusby K, et al. Survivin Is a Viable Target for the Treatment of Malignant Peripheral Nerve Sheath Tumors. Clin Cancer Res. 2012;18:2545–57.

5. Hui P, Li N, Johnson C, De Wever I, Sciot R, Manfioletti G, et al. HMGA proteins in malignant peripheral nerve sheath tumor and synovial sarcoma: preferential expression of HMGA2 in malignant peripheral nerve sheath tumor. Mod Pathol . 2005;18:1519–26.

6. Mohan P, Castellsague J, Jiang J, Allen K, Chen H, Nemirovsky O, et al. Genomic imbalance of HMMR/RHAMM regulates the sensitivity and response of malignant peripheral nerve sheath tumour cells to aurora kinase inhibition. Oncotarget . 2010;1:80–93.

7. Friedrich RE, Hagel C, Brehme Z, Kluwe L, Mautner VF. Ki-67 proliferation-index (MIB-1) of neurofibromas in neurofibromatosis type 1 patients. Anticancer Res . 2003;23:953–5.

8. Stricker TP, Henriksen KJ, Tonsgard JH, Montag AG, Krausz TN, Pytel P. Expression profiling of 519 kinase genes in matched

malignant peripheral nerve sheath tumor/plexiform neurofibroma samples is discriminatory and identifies mitotic regulators BUB1B, PBK and NEK2 as overexpressed with transformation. Mod Pathol. 2013;26:930–43.

9. Yu J, Deshmukh H, Payton JE, Dunham C, Scheithauer BW, Tihan T, et al. Array-Based Comparative Genomic Hybridization Identifies CDK4 and FOXM1 Alterations as Independent Predictors of Survival in Malignant Peripheral Nerve Sheath Tumor. Clin Cancer Res . 2011;17:1924–34.

10. Kresse SH, Skårn M, Ohnstad HO, Namløs HM, Bjerkehagen B, Myklebost O, et al. DNA copy number changes in high-grade malignant peripheral nerve sheath tumors by array CGH. Mol Cancer. 2008;7:48.

11. Yang J, Du X. Genomic and molecular aberrations in malignant peripheral nerve sheath tumor and their roles in personalized target therapy. Surg Oncol. 2013;22:e53–7.

12. Miller SJ, Lan ZD, Hardiman A, Wu J, Kordich JJ, Patmore DM, et al. Inhibition of Eyes Absent Homolog 4 expression induces malignant peripheral nerve sheath tumor necrosis. Oncogene. 2010;29:368–79.

13. Zhang P, Yang X, Ma X, Ingram DR, Lazar AJ, Torres KE, et al. Antitumor effects of pharmacological EZH2 inhibition on malignant peripheral nerve sheath tumor through the miR-30a and KPNB1 pathway. Mol Cancer. 2015;14:55.

14. Thomas LE, Winston J, Rad E, Mort M, Dodd KM, Tee AR, et al. Evaluation of copy number variation and gene expression in neurofibromatosis type-1-associated malignant peripheral nerve sheath tumours. Hum Genomics. 2015;9:3.

15. Pasmant E, Ortonne N, Rittiè L, Laurendeau I, Lèvy P, Lazar V, et al. Differential Expression of *CCN1* / *CYR61* , *CCN3/NOV* , *CCN4/WISP1* , and *CCN5/WISP2* in Neurofibromatosis Type 1 Tumorigenesis. J Neuropathol Exp Neurol. 2010;69:60–9.

16. Mantripragada KK, Spurlock G, Kluwe L, Chuzhanova N, Ferner RE, Frayling IM, et al. High-Resolution DNA Copy Number Profiling of Malignant Peripheral Nerve Sheath Tumors Using Targeted Microarray-Based Comparative Genomic Hybridization. Clin Cancer Res. 2008;14:1015–24.

17. Hakozaki M, Tajino T, Konno S, Kikuchi S, Yamada H, Yanagisawa M, et al. Overexpression of Cyclooxygenase-2 in Malignant Peripheral Nerve Sheath Tumor and Selective Cyclooxygenase-2 Inhibitor-Induced Apoptosis by Activating Caspases in Human Malignant Peripheral Nerve Sheath Tumor Cells. PLoS One. 2014;9:e88035.

18. Lévy P, Bièche I, Leroy K, Parfait B, Wechsler J, Laurendeau I, et al. Molecular Profiles of Neurofibromatosis Type 1-Associated Plexiform Neurofibromas. Clin Cancer Res. 2004;10.

19. Patel A V, Eaves D, Jessen WJ, Rizvi TA, Ecsedy JA, Qian MG, et al. Ras-driven transcriptome analysis identifies aurora kinase A as a potential malignant peripheral nerve sheath tumor therapeutic target. Clin Cancer Res. 2012;18:5020–30.

20. Miller SJ, Rangwala F, Williams J, Ackerman P, Kong S, Jegga AG, et al. Large-scale molecular comparison of human Schwann cells to malignant peripheral nerve sheath tumor cell lines and tissues. Cancer Res. 2006;66:2584–91.

21. Ågesen TH, Flørenes VA, Molenaar WM, Lind GE, Berner J-M, Plaat BEC, et al. Expression Patterns of Cell Cycle Components in Sporadic and Neurofibromatosis Type 1-Related Malignant Peripheral Nerve Sheath Tumors. J Neuropathol Exp Neurol. 2005;64.

22. Kang, J.H., Kim, O.S., Kim, J.H. et al. A novel mutation of exon 7 in growth hormone receptor mRNA in a patient with growth hormone insensitivity syndrome and neurofibromatosis type I. Int J Mol Med. 2012;

23. Kindblom LG, Ahldén M, Meis-Kindblom JM, Stenman G. Immunohistochemical and molecular analysis of p53, MDM2, proliferating cell nuclear antigen and Ki67 in benign and malignant peripheral nerve sheath tumours. Virchows Arch. 1995;427:19–26.
