# Supplementary material for: Gene signature associated with benign neurofibroma transformation to malignant peripheral nerve sheath tumors
Source: PLoS One. 2017 May 24;12(5):e0178316. doi: 10.1371/journal.pone.0178316 (PMC5443557; doi:10.1371/journal.pone.0178316)
Supplement: S12 Table — (PDF) [file pone.0178316.s012.pdf]

S12 Table: Down-regulated genes from the MPNST vs. NF signature previously related with NF and/or MPNST.

Rank	ENSGENE	hgnc_symbol	chrom_name	band	Relationship with NF or MPNST	Reference
235	ENSG00000188580	NKAIN2	6	q22.31	Deletion of its chromosomal region in NF1-associated cutaneous neurofibromas.	[1]
218	ENSG00000196154	S100A4	1	q21.3	Immunostaining reaction variable in malignant melanoma, and benign and malignant peripheral nerve sheath tumors.	[2]
208	ENSG00000197956	S100A6	1	q21.3	Its expression differentiates desmoplastic melanomas from MPNSTs but its diagnostic use is limited by overlapping.<br>Differential expression in malignant melanoma, and benign and malignant peripheral nerve sheath tumors.	[3]<br>[2]
163	ENSG00000197971	MBP	18	q23	Overexpressed in NF1-associated compared to sporadic MPNST.<br>One of the proteins complexed in RNA granules with NF1:LRPPRC.	[4]<br>[5]
147	ENSG00000152061	RABGAP1L	1	q25.1	Deletion of its chromosomal region in NF1-associated cutaneous neurofibromas.	[1]
139	ENSG00000105855	ITGB8	7	p21.1	MPNST-specific copy number variations.	[6]
129	ENSG00000168329	CX3CR1	3	p22.2	Chemokine receptor expressed from high to moderate levels in benign and malignant nerve sheath tumors.	[7]
128	ENSG00000107562	CXCL12	10	q11.21	High to moderate levels of expression in benign and malignant nerve sheath tumors.<br>Chemokine promoting cell proliferation in MPNSTs.	[7]<br>[8]
123	ENSG00000277632	CCL3	17	q12	Probably involved in recruitment of inflammatory cells by tumorigenic cells that could play a role in the auto-degeneration of Schwann cell tumors.	[9]
72	ENSG00000109099	PMP22	17	p12	Down-regulated in MPNST cell lines compared to normal Schwann cells.	[10]
63	ENSG00000261857	MIA	19	q13.2	Potential serum biomarker for tumor load in NF1.	[11]
59	ENSG00000158887	MPZ	1	q23.3	Down-regulated Schwann cell-specific gene in the comparison MPNST vs. pNF.	[12]
56	ENSG00000169554	ZEB2	2	q22.3	Transcription factor associated to the epithelial-mesenchymal transition in neurofibromatosis type 1, up-regulated in NF1-associated neurofibromas and Schwann cells.	[13]
42	ENSG00000189058	APOD	3	q29	Its expression increases with the formation of NFs and is down-regulated during malignant transformation of NFs.	[14]
41	ENSG00000189058	TIMP4	3	p25.2	Down-regulated in MPNST vs. pNF	[12]

					comparison.	
33	ENSG00000198910	L1CAM	X	q28	Down-regulated in MPNST vs. pNF comparison.	[12]
					Overexpressed in NF1-associated vs. sporadic MPNSTs.	[4]
					Highly expressed in benign NF1 tumors and down-regulated in MPNST vs. benign NF1-associated tumors.	[15]
28	ENSG00000065361	ERBB3	12	q13.2	Its expression is rare in NF1-related tumors, particularly in MPNST.	[16]
					Down-regulated in MPNST vs. pNF comparison.	[12]
					Variable expression in NFs and MPNSTs.	[17]
					It is present in MPNST invadopodia.	[18]
23	ENSG00000132470	ITGB4	17	q25.1	Down-regulated in MPNST vs. pNF comparison.	[12]
					Its genomic region was amplified; copy number changes of this gene were observed in 32% of MPNSTs.	[19]
					Included in a significant gain aberration.	[20]
21	ENSG00000101265	RASSF2	20	p13	Down-regulated in MPNST vs. pNF comparison.	[12]
					Included in a significant deletion aberration.	[20]
18	ENSG00000100146	SOX10	22	q13.1	Down-regulated in MPNST vs. pNF comparison.	[12]
					Down-regulated in comparison MPNST vs. Schwann cells.	[10]
					More specific than S100 in diagnosis of neural crest-derived tumors.	[21]
					Its complete silencing discriminates MPNSTs and cellular schwannomas.	[22]
9	ENSG00000168477	TNXB	6	p21.32	Down-regulated in MPNST vs. pNF comparison.	[12]
5	ENSG00000071991	CDH19	18	q22.1	Significantly up-regulated in NF1 tumors. Potential role in dermal neurofibroma initiation.	[23]
2	ENSG00000160307	S100B	21	q22.3	Down-regulated in MPNST vs. pNF comparison.	[12]
					Diffuse staining in malignant melanoma and benign peripheral nerve sheath tumors; it reacts in 30% of MPNSTs.	[2]

1. Asai A, Karnan S, Ota A, Takahashi M, Damdindorj L, Konishi Y, et al. High-resolution 400K oligonucleotide array comparative genomic hybridization analysis of neurofibromatosis type 1-associated cutaneous neurofibromas. Gene. 2015;558:220–6.

2. Nonaka D, Chiriboga L, Rubin BP. Differential expression of S100 protein subtypes in malignant melanoma, and benign and malignant peripheral nerve sheath tumors. J Cutan Pathol. 2008;35:1014–9.

3. Fullen DR, Garrisi AJ, Sanders D, Thomas D. Expression of S100A6 protein in a broad spectrum of cutaneous tumors using tissue microarrays. J Cutan Pathol. 2008;35:28–34.

4. Watson MA, Perry A, Tihan T, Prayson RA, Guha A, Bridge J, et al. Gene expression profiling reveals unique molecular subtypes of Neurofibromatosis Type I-associated and sporadic malignant peripheral nerve sheath tumors. Brain Pathol . 2004;14:297–303.

5. Arun V, Wiley JC, Kaur H, Kaplan DR, Guha A. A novel neurofibromin (NF1) interaction with the leucine-rich pentatricopeptide repeat motif-containing protein links neurofibromatosis type 1 and the french canadian variant of leigh’s syndrome in a common molecular complex. J Neurosci Res. 2013;91:494–505.

6. Upadhyaya M, Spurlock G, Thomas L, Thomas NST, Richards M, Mautner VF, et al. Microarray-based copy number analysis of neurofibromatosis type-1 (NF1)-associated malignant peripheral nerve sheath tumors reveals a role for Rho-GTPase pathway genes in NF1 tumorigenesis. Hum Mutat. 2012;33:763–76.

7. Hattermann K, Li G, Hugo H-H, Mentlein R, Mehdorn HM, Held-Feindt J. Expression of the chemokines CXCL12 and CX3CL1 and their receptors in human nerve sheath tumors. Histol Histopathol. 2013;28:1337–49.

8. Mo W, Chen J, Patel A, Zhang L, Chau V, Li Y, et al. CXCR4/CXCL12 Mediate Autocrine Cell- Cycle Progression in NF1-Associated Malignant Peripheral Nerve Sheath Tumors. Cell. 2013;152:1077–90.

9. Mori K, Chano T, Yamamoto K, Matsusue Y, Okabe H. Expression of macrophage inflammatory protein-1alpha in Schwann cell tumors. Neuropathology. 2004;24:131–5.

10. Miller SJ, Rangwala F, Williams J, Ackerman P, Kong S, Jegga AG, et al. Large-scale molecular comparison of human Schwann cells to malignant peripheral nerve sheath tumor cell lines and tissues. Cancer Res. 2006;66:2584–91.

11. Kolanczyk M, Mautner V, Kossler N, Nguyen R, Kühnisch J, Zemojtel T, et al. MIA is a potential biomarker for tumour load in neurofibromatosis type 1. BMC Med. 2011;9:82.

12. Lévy P, Vidaud D, Leroy K, Laurendeau I, Wechsler J, Bolasco G, et al. Molecular profiling of malignant peripheral nerve sheath tumors associated with neurofibromatosis type 1, based on large-scale real-time RT-PCR. Mol Cancer. 2004;3:20.

13. Arima Y, Hayashi H, Kamata K, Goto TM, Sasaki M, Kuramochi A, et al. Decreased expression of neurofibromin contributes to epithelial-mesenchymal transition in neurofibromatosis type 1. Exp Dermatol. 2009;19:e136–41.

14. HUNTER S, WEISS S, OU C, JAYE D, YOUNG A, WILCOX J, et al. Apolipoprotein D is down-regulated during malignant transformation of neurofibromas. Hum Pathol. 2005;36:987–93.

15. Blessmann M, Gröbe A, Quaas A, Kaifi JT, Mistakidis G, Bernreuther C, et al. Adhesion molecule L1 is down-regulated in malignant peripheral nerve sheath tumors versus benign neurofibromatosis type 1–associated tumors. Oral Surg Oral Med Oral Pathol Oral Radiol. 2012;113:239–44.

16. Li H, Velasco-Miguel S, Vass WC, Parada LF, DeClue JE. Epidermal growth factor receptor signaling pathways are associated with tumorigenesis in the Nf1:p53 mouse tumor model. Cancer Res. 2002;62:4507–13.

17. Stonecypher MS, Byer SJ, Grizzle WE, Carroll SL. Activation of the neuregulin-1/ErbB signaling pathway promotes the proliferation of neoplastic Schwann cells in human malignant peripheral nerve sheath tumors. Oncogene. 2005;24:5589–605.

18. Eckert JM, Byer SJ, Clodfelder-Miller BJ, Carroll SL. Neuregulin-1β and neuregulin-1α differentially affect the migration and invasion of malignant peripheral nerve sheath tumor cells. Glia. 2009 ;57:1501–20.

19. Mantripragada KK, Spurlock G, Kluwe L, Chuzhanova N, Ferner RE, Frayling IM, et al. High-Resolution DNA Copy Number Profiling of Malignant Peripheral Nerve Sheath Tumors Using Targeted Microarray-Based Comparative Genomic Hybridization. Clin Cancer Res. 2008;14:1015–24.

20. Yang J, Du X. Genomic and molecular aberrations in malignant peripheral nerve sheath tumor and their roles in personalized target therapy. Surg Oncol. 2013;22:e53–7.

21. Karamchandani JR, Nielsen TO, van de Rijn M, West RB. Sox10 and S100 in the Diagnosis of Soft-tissue Neoplasms. Appl Immunohistochem Mol Morphol. 2012;20:445–50.

22. Pekmezci M, Reuss DE, Hirbe AC, Dahiya S, Gutmann DH, von Deimling A, et al. Morphologic and immunohistochemical features of malignant peripheral nerve sheath tumors and cellular schwannomas. Mod Pathol. 2015;28:187–200.

23. Iribar H, Jaka A, Ormaechea N, Tuneu A, Izeta A, Gutiérrez-Rivera A. Does Schwann cell dedifferentiation originate dermal neurofibromas? Exp Dermatol. 2016;25:901–3.
