# Supplementary material for: Gene signature associated with benign neurofibroma transformation to malignant peripheral nerve sheath tumors
Source: PLoS One. 2017 May 24;12(5):e0178316. doi: 10.1371/journal.pone.0178316 (PMC5443557; doi:10.1371/journal.pone.0178316)
Supplement: S13 Table — (PDF) [file pone.0178316.s013.pdf]

S13 Table: Head and tail of GSE66743 data extracted from differential expression analysis between MPNST and NF phenotypes with other factors required to compute gene scores.

ENSGENE	hgnc_symbol	logFC[1]	logFC_m[2]	pval	adj.pval	B	MAD	FII[3]	S(logFC_m)[4]	score[5]
ENSG00000185686	PRAME	5.41	7.11	1.14e-04	4.02e-04	0.97	1.74	0.76	1.00	0.76
ENSG00000006283	CACNA1G	5.10	5.84	9.27e-06	9.27e-06	3.37	1.92	0.67	0.93	0.63
ENSG00000131747	TOP2A	4.46	4.73	5.63e-10	5.63e-10	12.72	1.16	0.75	0.76	0.57
ENSG00000089685	BIRC5	4.40	4.59	3.10e-09	3.10e-09	11.08	1.22	0.73	0.73	0.54
ENSG00000128045	RASL11B	4.12	4.66	8.23e-06	8.23e-06	3.48	1.38	0.70	0.74	0.52
ENSG00000166803	KIAA0101	4.44	4.56	1.86e-10	1.86e-10	13.78	1.39	0.70	0.73	0.51
ENSG00000142973	CYP4B1	-4.79	-5.24	4.11e-06	5.14e-05	4.15	2.17	0.56	-0.80	-0.47
ENSG00000114854	TNNC1	-5.00	-6.17	1.31e-04	4.38e-04	0.84	3.04	0.51	-0.94	-0.48
ENSG00000171819	ANGPTL7	-5.27	-5.89	8.29e-06	8.29e-05	3.48	2.49	0.58	-0.90	-0.52
ENSG00000107317	PTGDS	-5.87	-5.65	4.46e-07	4.46e-07	6.29	2.24	0.60	-0.86	-0.52
ENSG00000148671	ADIRF	-5.19	-5.43	6.95e-07	6.95e-07	5.86	1.89	0.65	-0.83	-0.54
ENSG00000196616	ADH1B	-6.38	-6.74	5.28e-06	5.28e-06	3.91	2.95	0.56	-1.00	-0.56

[1]Fold Change computed based on the mean of expression ratios between phenotypes (obtained from limma).

[2]Fold Change computed according to the median of expression ratios between phenotypes.

[3]Factor II included in the formula to compute the scores: $[1-(MAD_{ij}/abs(logFC\_m_{ij}))]$.

[4]Normalized positive and negative values of logFC_m regarding logFC_m quantiles 0.9995 and 0.0005, respectively.

[5]$S(logFC\_m_{ij})*[1-(MAD_{ij}/abs(logFC\_m_{ij}))]*(1-pval_{ij})$
