# Supplementary figures and images for: Gene signature associated with benign neurofibroma transformation to malignant peripheral nerve sheath tumors
Source: PLoS One. 2017 May 24;12(5):e0178316. doi: 10.1371/journal.pone.0178316 (PMC5443557; doi:10.1371/journal.pone.0178316)

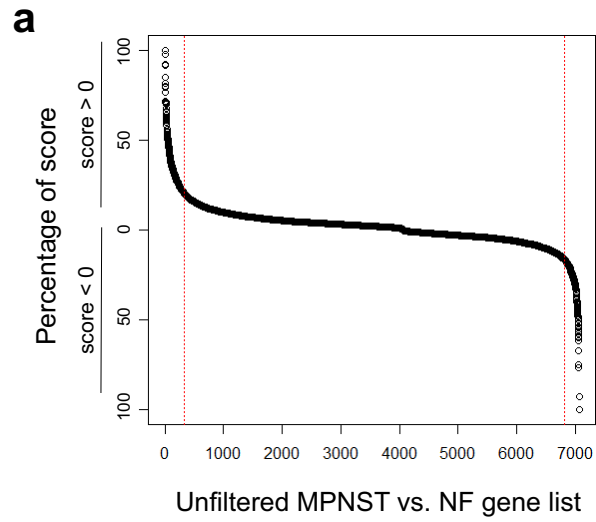

**b**

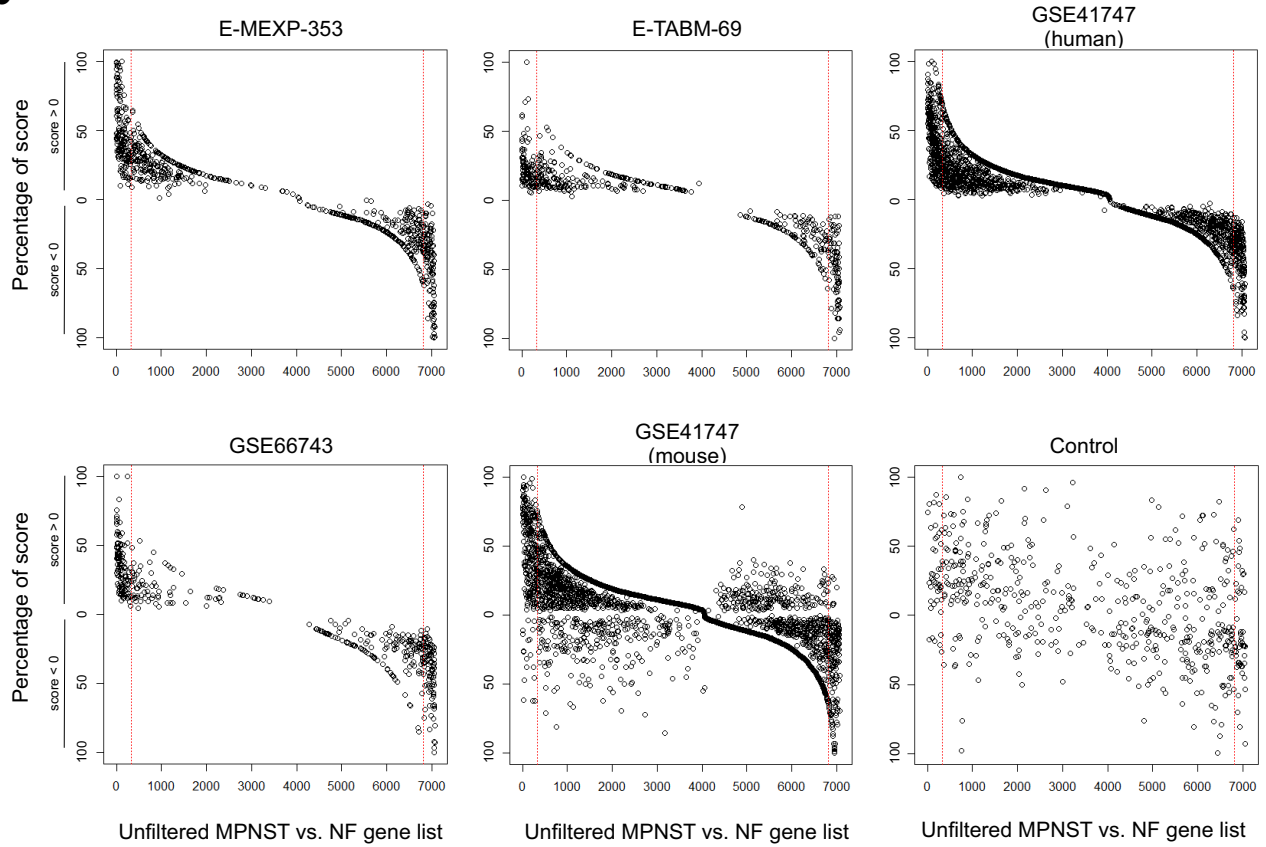

Supplement: S1 Fig — a. Plot showing the percentage of score values for up- (score>0) and down- (score<0) regulated genes of the MPNST vs. NF unfiltered list. This list contains the score values of the 7064 unique ENSEMBL human genes sorted in x-axis from the highest to the lowest score value (Table A in S2 Table). Vertical red dot lines discriminate the first 336 up- and the last 243 downregulated genes with the highest absolute score values, included in the MPNST vs. NF gene signature. b. Plots with x and y axes equal to plot A showing the percentage of score values from non-null score genes represented in each of the five studies integrated in the MPNST vs. NF gene signature (E-MEXP-353, E-TABM-69, GSE41747 (human), GSE66743 and GSE41747 (mouse)). Unlike these five plots, that contain differentially expressed genes derived from the MPNST vs. NF comparison, the last plot (Control), as negative control, includes non-null score genes obtained in the NF vs. Control cell culture comparison from the GSE14038 accession. (PDF) [file pone.0178316.s015.pdf]

**a**

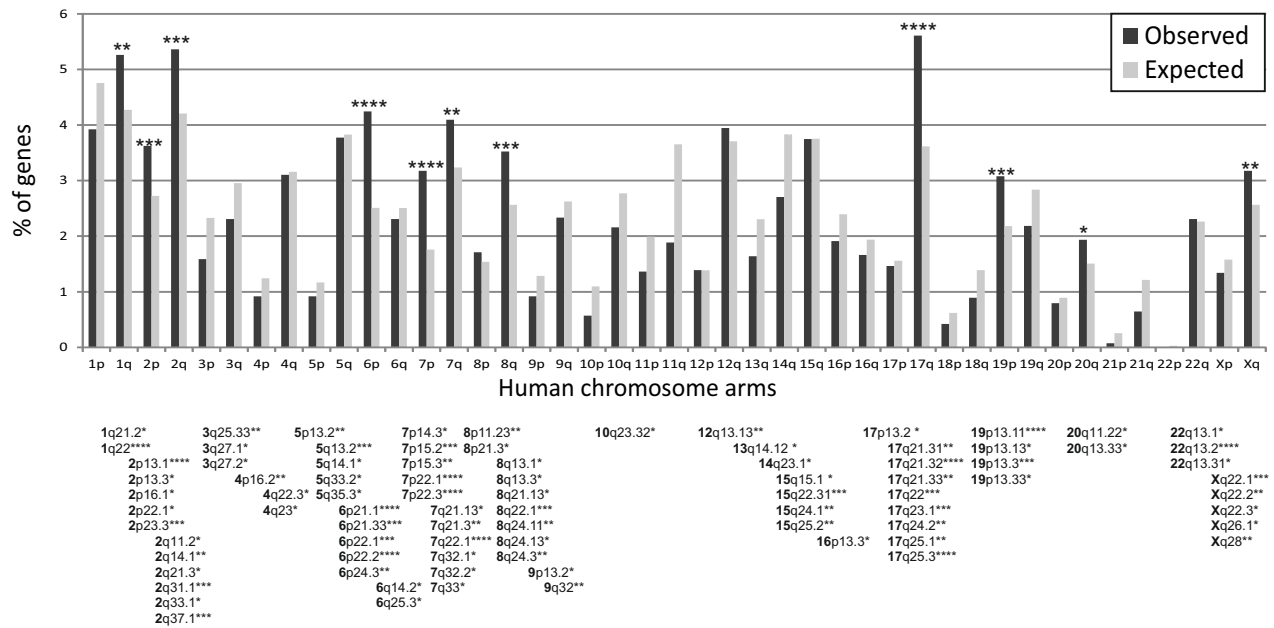

**b**

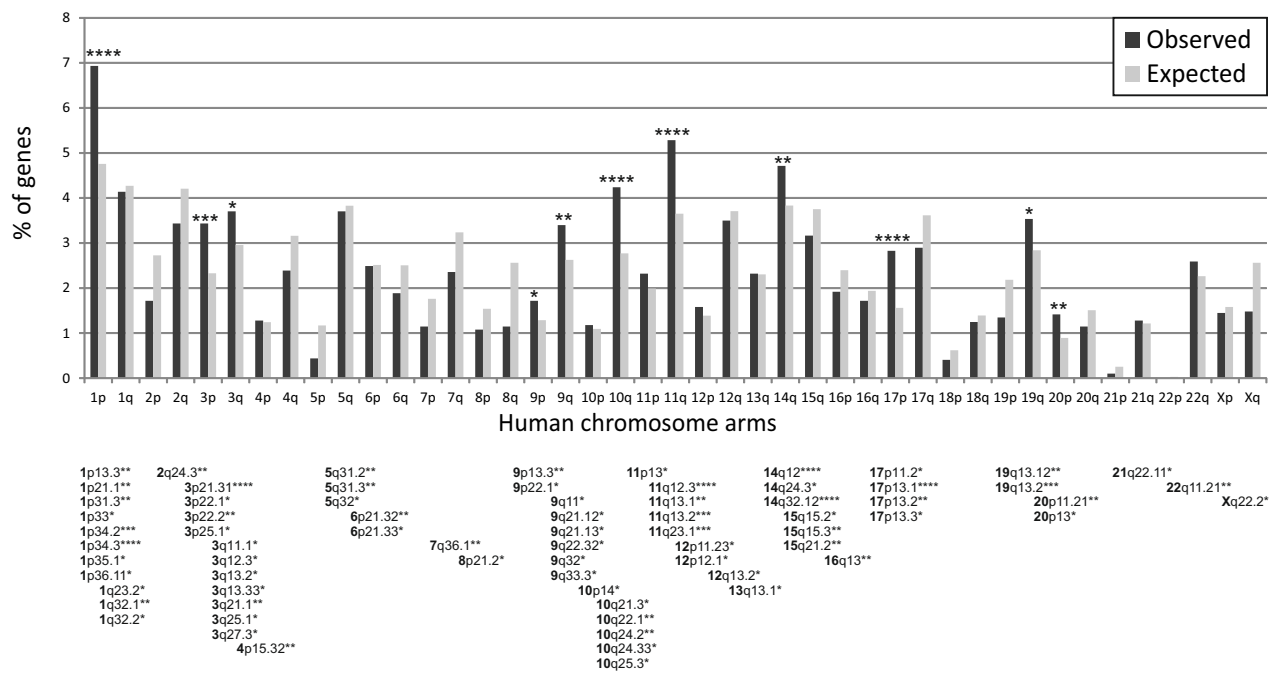

Supplement: S2 Fig — The distribution was calculated from 4059 genes with positive score (a), and from 3005 genes with negative score (b). Bar diagrams compare the observed distribution of MPNST vs. NF gene percentage in the human chromosome arms (blue bars) with the expected distribution according to the human ENSEMBL database (red bars). Statistical significance of the gene signature over-represented chromosome arms is above the bars. Over-represented human chromosome bands in the MPNST vs. NF gene signature are shown below each chart. Their statistical significance is shown at the top right side of band names. (****) P(X≥x) < 0.0001, (***) 0.0001< P(X≥x) < 0.001, (**) 0.001< P(X≥x) < 0.01, (*) 0.01< P(X≥x) < 0.05. (PDF) [file pone.0178316.s016.pdf]

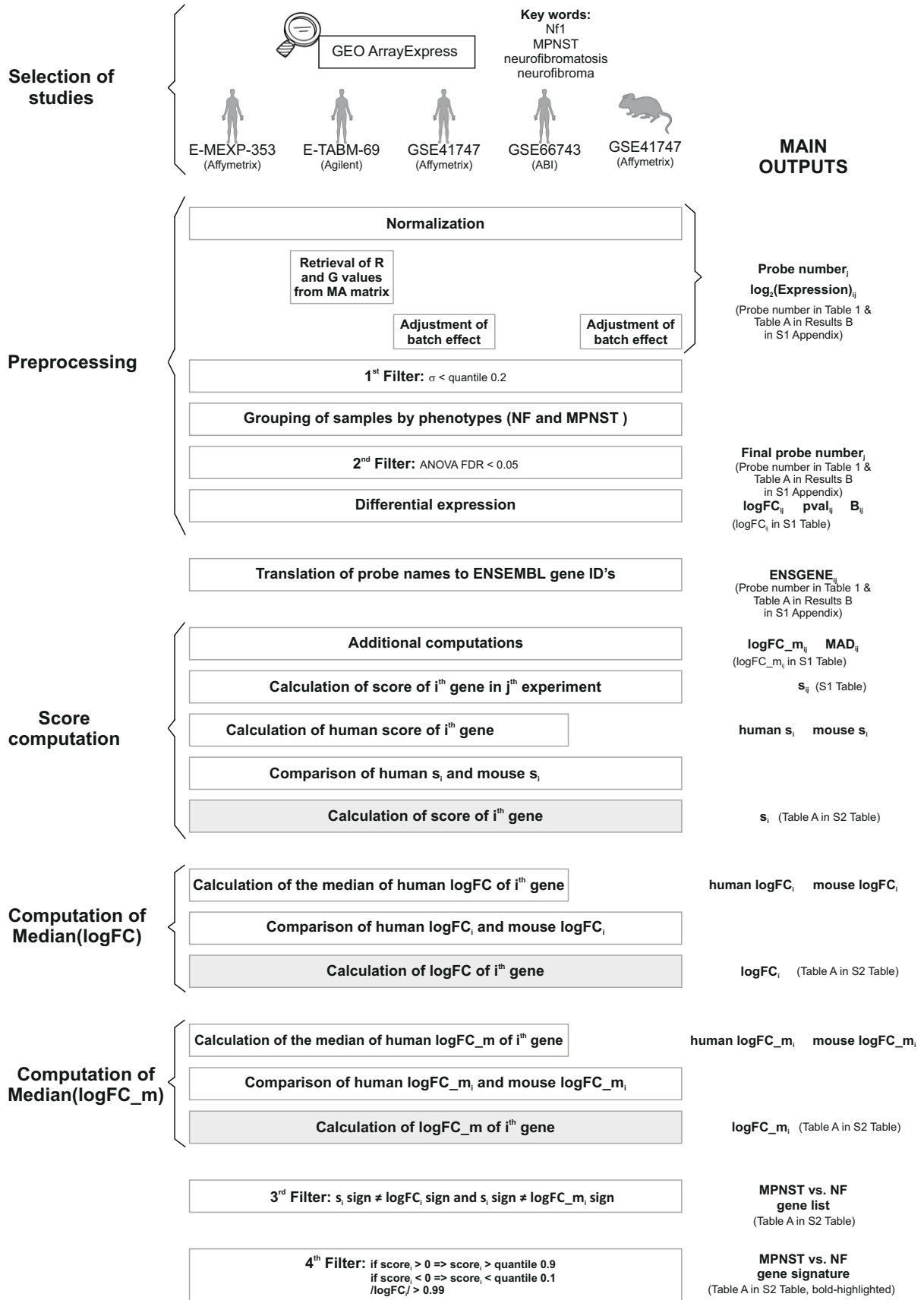

Supplement: S4 Fig — Main outputs of some individual steps appear on the right. i: Each individual gene. j: Each individual study. (PDF) [file pone.0178316.s018.pdf]

**GSE14038** 36,6571 final probes

- MPNST\_cells
- MPNST
- NFHSC
- NF
- NHSC

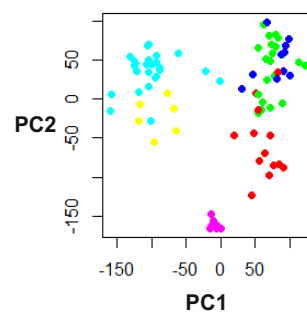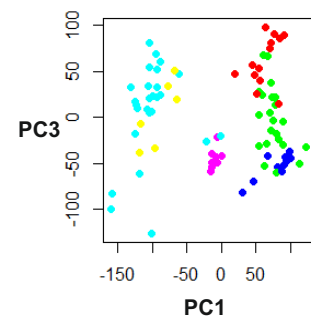

**GSE39764** 33,418 final probes

- MPNST
- NHSC

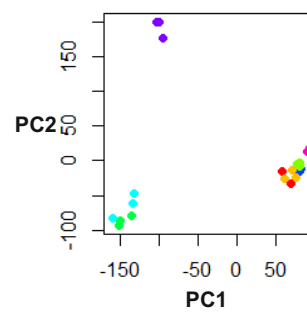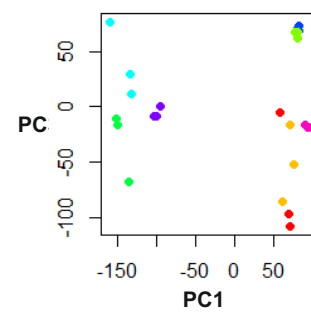

Supplement: S6 Fig — The final number of probes (Table A in Results B in S1 Appendix) considered in the computation of PCA plots is shown. Colored circles on the left show the sample phenotypes compared in the analyses. PCA plot from GSE14038 (cell cultures) includes the samples from the previously described comparison MPNST vs. NF from GSE41747 (human tumor tissue) depicted in S5 Fig. (PDF) [file pone.0178316.s020.pdf]
