# Supplementary material for: Gene signature associated with benign neurofibroma transformation to malignant peripheral nerve sheath tumors
Source: PLoS One. 2017 May 24;12(5):e0178316. doi: 10.1371/journal.pone.0178316 (PMC5443557; doi:10.1371/journal.pone.0178316)
Supplement: S3 Fig — Numbers over the gray scale diagram indicate the number of genes included in each cluster. The interval of color scale values is shown below the diagram. The right side of diagram details the number of genes in each cluster, the number of biological process GO terms over-represented in each cluster, and the summary of that GO term enrichment as functional characterization of clusters. A complete list of terms is shown in S9 Table. (PDF) [file pone.0178316.s017.pdf]

Cell cultures			Nerve tumors						
NF_vs_Control	MPNST_vs_Control	MPNST_vs_NF	MPNST_vs_NF	MPNST_vs_Control	NF_vs_Control	Cluster	Nº of genes	GO terms	Functional enrichment
0	9	3	23	9	1	1	26		
0	27	11	13	12	1	2	30		
0	49	31	38	11	5	3	65	1	peripheral nervous system development
13	2	8	5	4	6	4	29		
6	6	0	4	9	1	5	12	12	positive regulation of cell proliferation; heart development; outflow tract morphogenesis
2	1	0	6	1	1	6	8	6	skin morphogenesis; renal filtration; extracellular matrix organization
2	0	3	1	5	1	7	9	2	extracellular matrix organization
15	70	4	3	4	2	8	76		
0	9	0	6	3	0	9	11		
6	9	0	2	10	3	10	13		
3	9	0	0	1	2	11	10		
3	2	1	0	0	0	12	5		
10	5	0	1	2	0	13	10		
0	21	3	0	5	4	14	22		
0	12	0	1	0	1	15	13		
2	123	67	17	27	23	16	167	51	immune response; response to external substances; regulation of signal transduction; development
0	2	0	24	4	49	17	70		
1	10	5	0	10	23	18	29	3	nervous system development
0	3	0	0	3	27	19	27		
3	8	3	5	49	152	20	171	23	cell adhesion; synapsis; behavior; nervous system development
2	5	0	16	90	47	21	108	2	single-organism cellular process
1	3	0	52	78	6	22	100	25	extracellular matrix organization; skeletal system development; ossification
1	5	13	26	35	0	23	49	34	DNA replication; mitosis; regulation of cell cycle process
0	8	5	40	22	2	24	51	25	DNA replication; DNA repair; mitosis; regulation of mitotic cell cycle phase transition
2	0	6	136	124	2	25	153	181	cell proliferation; DNA replication; mitosis; positive regulation of mitosis; meiosis
0	1	0	19	40	13	26	45		
1	6	0	10	17	3	27	21		
0	0	0	2	15	0	28	16		
0	2	1	2	10	3	29	13	2	cellular response to jasmonic acid stimulus
8	2	1	2	8	7	30	19		
1	7	0	4	126	109	31	171		
2	6	7	1	49	127	32	147		
0	31	1	27	12	0	33	53		
1	3	0	42	14	7	34	49		
13	68	2	0	0	2	35	68	15	urogenital system development; regulation of cell migration; response to abiotic stimulus
8	26	0	0	9	10	36	30	1	carbohydrate derivative biosynthetic process
8	29	0	12	11	2	37	29	15	outer ear morphogenesis; embryonic limb morphogenesis; vascular system development
0	37	26	2	1	1	38	45		
2	52	33	3	5	3	39	69		
0	24	25	8	9	1	40	35		
0	19	25	3	7	3	41	33	3	anterior/posterior pattern specification
0	20	4	1	9	4	42	23	1	embryonic hindlimb morphogenesis
0	21	0	1	7	2	43	21		
0	31	3	3	4	0	44	31	2	mesoderm development
0	7	2	7	6	2	45	16		
1	7	2	5	2	1	46	11	2	commissural neuron axon guidance; chondrocyte differentiation

0% 23%
