# Supplementary material for: Gene signature associated with benign neurofibroma transformation to malignant peripheral nerve sheath tumors
Source: PLoS One. 2017 May 24;12(5):e0178316. doi: 10.1371/journal.pone.0178316 (PMC5443557; doi:10.1371/journal.pone.0178316)
Supplement: S5 Fig — The final number of probes (Table A in Results B in S1 Appendix) considered in the computation of PCA plots is shown. The legend of colored circles on the left shows sample phenotypes compared. (PDF) [file pone.0178316.s019.pdf]

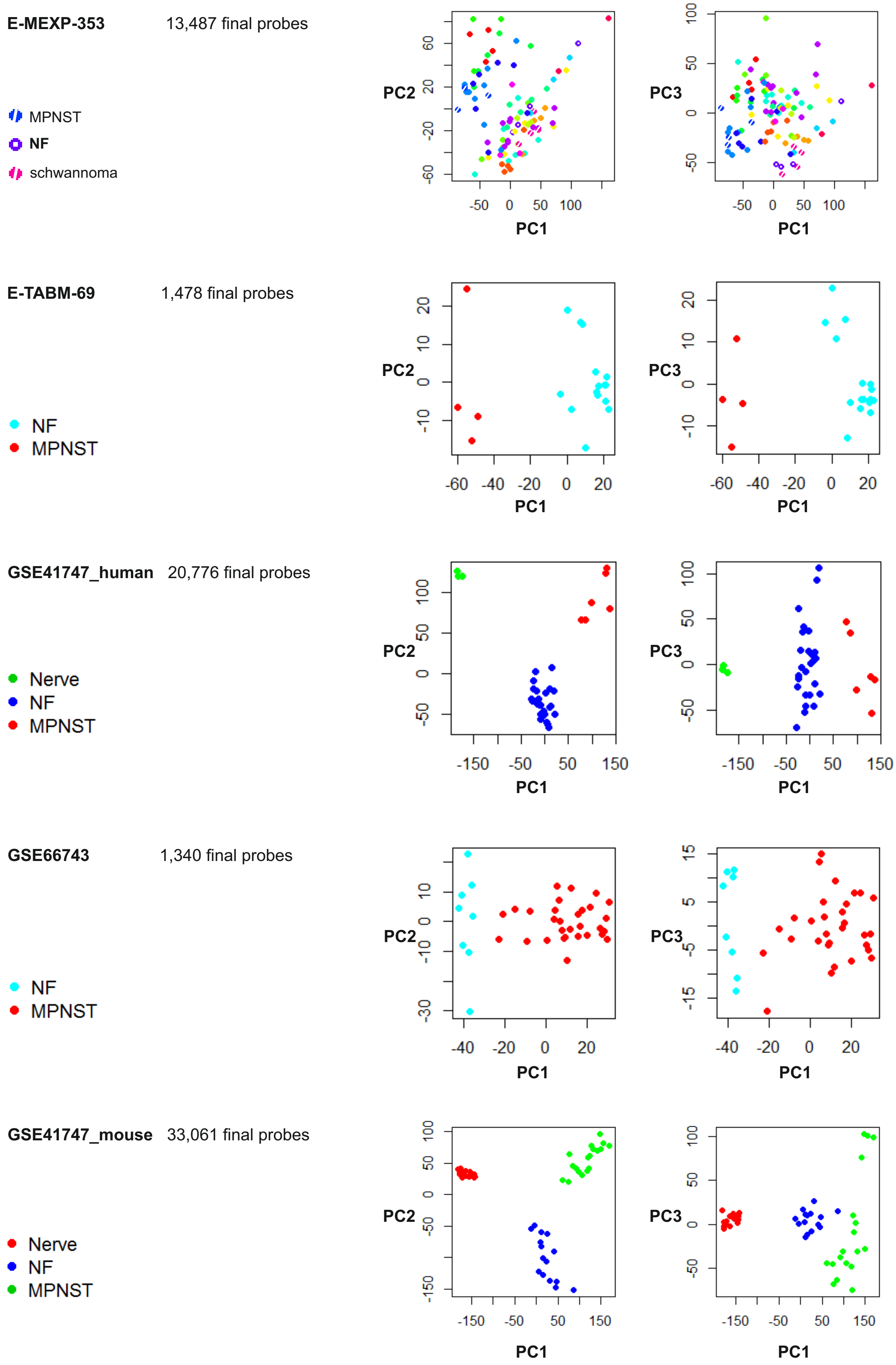

E-MEXP-353
13,487 final probes
MPNST
NF
schwannoma
PC1
PC2
PC3
E-TABM-69
1,478 final probes
NF
MPNST
GSE41747_human
20,776 final probes
Nerve
NF
MPNST
GSE66743
1,340 final probes
NF
MPNST
GSE41747_mouse
33,061 final probes
Nerve
NF
MPNST
