# Supplementary material for: Gene signature associated with benign neurofibroma transformation to malignant peripheral nerve sheath tumors
Source: PLoS One. 2017 May 24;12(5):e0178316. doi: 10.1371/journal.pone.0178316 (PMC5443557; doi:10.1371/journal.pone.0178316)
Supplement: S1 Appendix — Results A: Relationships between score values and additional attributes in the MPNST vs. NF gene signature. Results B: Contribution of individual studies to the MPNST vs. NF gene signature. Results C: Genes included in main functional pathways associated to the MPNST vs. NF signature. Results D: Characterization of additional NF1-related gene signatures. Results E: Homogeneity of gene profiles in each SOTA cluster. Discussion A: Comparison between over-represented chromosome regions in the MPNST vs. NF gene signature and previously described MPNST aberrant chromosome modifications. Discussion B: Expression profile differences between cultured cells and nerve tumors observed in SOX9, SUZ12, EGFR, SPP1 and BMP2 genes. Discussion C: Panel of genes potentially silenced by hypermethylation of their CpG-island promoter region. Discussion D: HDAC inhibitors counteract repression of CBX7 and over-expression of EZH2. Discussion E: Supplementation with acetate precursors as coadjuvant chemotherapy. Materials and methods A: Microarray data pre-processing. Materials and methods B: Translation from probe names to human ENSEMBL gene IDs, HUGO IDs and mapping in human chromosome arms. Materials and methods C: Scores of genes across studies and final score for each gene in a comparison between two phenotypes. Materials and methods D: Computation of bias in score values among studies: Bhattacharya distance (BD) ratio. Materials and methods E: DNA methylation analysis. (PDF) [file pone.0178316.s021.pdf]

S1 Appendix

Index

Results A: Relationships between score values and additional attributes in the MPNST vs. NF gene signature.
Results B: Contribution of individual studies to the MPNST vs. NF gene signature.
Results C: Genes included in main functional pathways associated to the MPNST vs. NF signature.
Results D: Characterization of additional NF1-related gene signatures.
Results E: Homogeneity of gene profiles in each SOTA cluster.

Discussion A: Comparison between over-represented chromosome regions in the MPNST vs. NF gene signature and previously described MPNST aberrant chromosome modifications.
Discussion B: Expression profile differences between cultured cells and nerve tumors observed in *SOX9*, *SUZ12*, *EGFR*, *SPP1* and *BMP2* genes.
Discussion C: Panel of genes potentially silenced by hypermethylation of their CpG-island promoter region.
Discussion D: HDAC inhibitors counteract repression of CBX7 and over-expression of EZH2.
Discussion E: Supplementation with acetate precursors as coadjuvant chemotherapy.

Materials and methods A: Microarray data pre-processing.
Materials and methods B: Translation from probe names to human ENSEMBL gene IDs, HUGO IDs and mapping in human chromosome arms.
Materials and methods C: Scores of genes across studies and final score for each gene in a comparison between two phenotypes.
Materials and methods D: Computation of bias in score values among studies: Bhattacharya distance (BD) ratio.
Materials and methods E: DNA methylation analysis.

References

Results A: Relationships between score values and additional attributes in the MPNST vs. NF gene signature

Gene logFC values across the studies from the MPNST vs. NF signature (bold-highlighted in Table A in S2 Table) were plotted regarding gene score values (Fig A (a)). A positive correlation between gene logFC and score values was inferred (Pearson's product-moment correlation 0.94; pval 2.2e-16; 95% confidence interval from 0.93 to 0.95). Red dots identify 114 genes for which mouse scores were not included in the final computation of gene scores, due to the absence of those genes in the mouse study, or because mouse and human studies showed different behavior. Most of genes ignoring mouse data located in the interval [-1, 1] of score values. Although the majority of these genes accumulated around the logFC interval [-2, 2], the highest logFC absolute values were similar in genes considering or not mouse data. Due to a high correlation between logFC and logFC_m values (Pearson's product-moment correlation 0.9962; pval 2.2e-16; 95% confidence interval from 0.9956 to 0.9969; Fig A (b)), a similar plot could be inferred when logFC_m and score values were compared.

To assess the homogenous contribution of each study to the final gene scores, we computed the Bhattacharyya distance ratio (BD-ratio). The lower was the BD-ratio, the more homogeneous was the contribution of the studies. 530 genes, 91.54% of the total gene signature, showed BD-ratios between 0 and 100. These genes, represented by more than one study, showed higher absolute scores compared with genes with BD-ratios 0 or 100, as the distribution of genes indicates considering both BD-ratio and score values (Fig A (c)). Null BD-ratios were assigned to 5 genes (less than 1%) that were in only one study, to distinguish them from 44 genes (7.60%), present in more than one study, with all score values null except one, which results in a BD-ratio of 100. This maximum value for BD-ratio remarks the unequal contribution of studies to the gene score computation of these 44 genes. A slight negative correlation existed between BD-ratios and absolute score values for genes with BD-ratios in the interval (0, 100) (Pearson's product-moment correlation -0.097; pval < 0.01968; 95% confidence interval from -0.177 to -0.016). Again, red dots denote genes excluding mouse data. Genes with absolute score values higher than 1 and lowest BD-ratios, for which individual experiments similarly contribute to the final score, mostly included mouse data in the computation of gene scores.

The number of genes included in the MPNST vs. NF signature with final score

calculated starting from 5, 4, 3, 2, or 1 study, both for up- and downregulated genes, is shown in Fig A (d). As the chart indicates, the highest amount of genes depended on 5 studies (56.31%), 4 from human and one from mouse. 26.94%, 10.88%, 5% and 0.86% of genes depended on 4, 3, 2 and 1 study, respectively. As expected from the addition of five individual scores, the highest absolute average scores were obtained for genes represented in a higher number of studies.

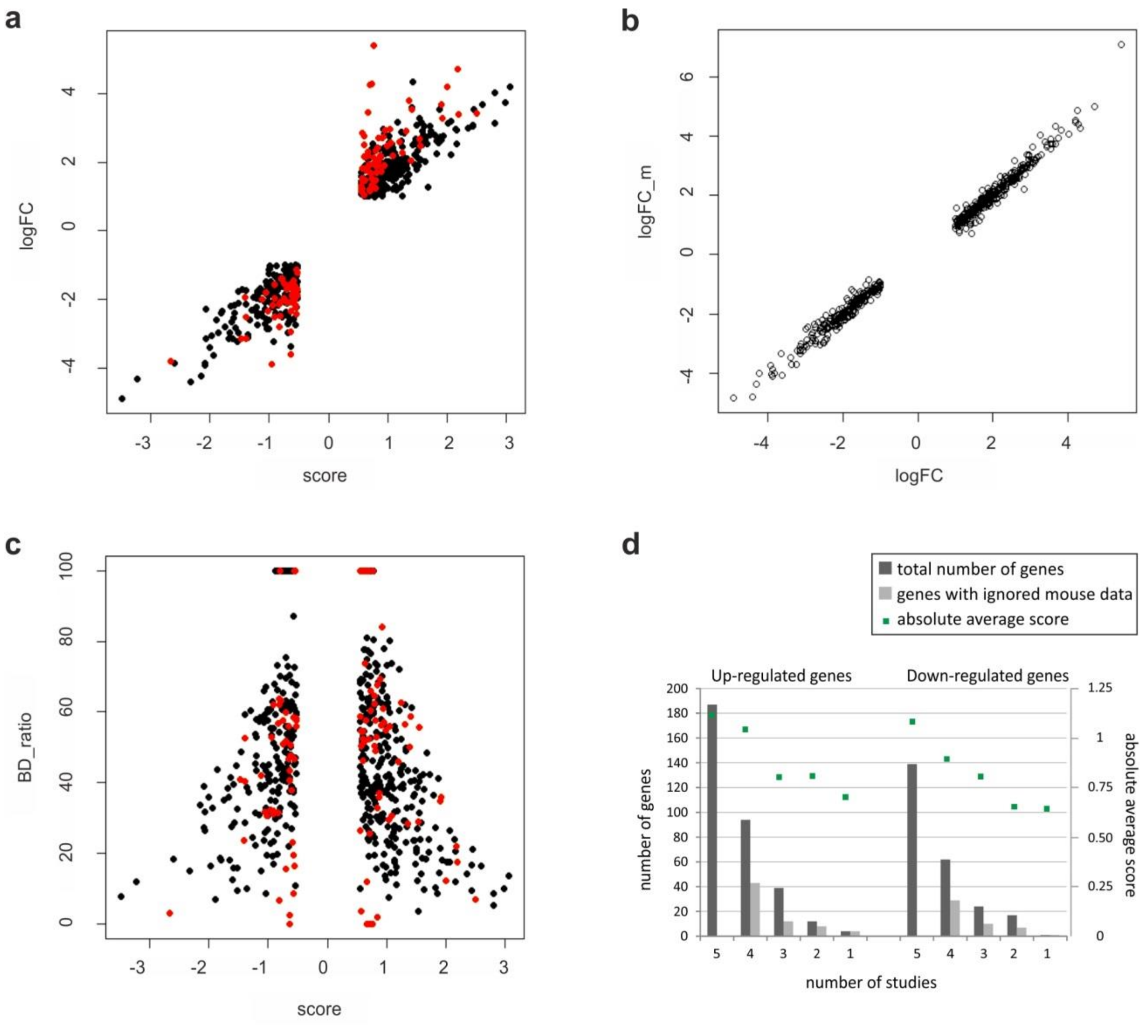

Fig A: Relationships between score values and additional attributes from MPNST vs. NF gene signature. a. The dot plot represents the 579 genes from the MPNST vs. NF gene signature. The plot compares for each gene the logFC with the score value. Red dots represent 114 genes in which mouse data were ignored in the computation of final score and only human data were taken into account. **b.** Same plot from a to compare values of logFC and logFC_m. **c.** Dot plot that compares BD_ratios with score values from genes included in MPNST vs. NF signature. The meaning of red dots was indicated in a. **d.** Chart comparing the number of genes from up- and down-regulated genes included in the MPNST vs. NF signature, and the absolute average score, with the number of studies covering those genes.

Results B: Contribution of individual studies to the MPNST vs. NF gene signature

Although the five individual studies show similar patterns of gene score distribution (S1 Fig), they contribute unequally to the MPNST vs. NF gene signature. Both human and mouse studies from the GSE41747 accession contributed with the highest number of genes to the gene signature, and studies E-TABM-69 and GSE66743 with the lowest (S1 Fig and Table A). However, the amount of genes from studies E-TABM-69 and GSE66743 present in the MPNST vs. NF gene signature considerably increased in comparison with the previous unfiltered gene list. Only 7.86 and 6.66% of genes with non-null score from individual studies E-TABM-69 and GSE66743, respectively, were in the unfiltered gene list, whereas these ratios arose to 34.71% and 39.72% in the MPNST vs. NF gene signature. The addition of the gene number from all studies that contribute to the unfiltered gene list covers 1.32 times the length of the unfiltered gene list (first column score ≠ 0 divided by 7064). In contrast, the total amount of genes from all studies included in the MPNST vs. NF gene signature covered 2.82 times the length of the gene signature (second column score ≠ 0 divided by 579). This indicates that genes included in the final gene signature relied on score values computed from a higher amount of studies than genes from the unfiltered list. This result agreed with the above described BD-ratios in which 91.53% of genes from the gene signature relied on score values computed from more than one study.

Table A: Microarray studies selected from GEO and ArrayExpress databases included in MPNST vs. NF meta-analysis

Organism	Accession	Probe number	Final probe number[1]	adj.pval[2] <0.05 (ENSEMBL gene number)	Unfiltered MPNST vs. NF gene list (7064 genes)				MPNST vs. NF gene signature (579 genes)			
					score ≠ 0 (ENSEMBL gene number)	max (adj.pval[2])	max(logFC[2]) [score < 0]	min(logFC[2]) [score > 0]	score ≠ 0 (ENSEMBL gene number)	max (adj.pval[2])	max(logFC[2]) [score < 0]	min(logFC[2]) [score > 0]
Human	E-MEXP-353[3]	22283	13,487	2,670	850	0.00351	-0.5328	0.5878	293	0.00351	-0.7374	0.8759
	E-TABM-69[3]	19061	1,478	1,352	555	0.00054	-0.4648	0.4649	201	0.00052	-0.6420	0.7297
	GSE41747[4,5]	54675	20,776	7,909	3,108	0.00082	-0.3467	0.3194	512	0.00079	-0.9829	0.6547
	GSE66743[4]	33025	1,340	989	471	0.00071	-0.9010	0.8691	230	0.00071	-1.3152	0.9312
Mouse	GSE41747[4]	45101	33,061	10,982	4,307	0.00036	-0.1959	0.2262	394	0.00025	-0.4115	0.4626

[1]Obtained after the first two screening steps of preprocessing of microarray studies.

[2]adj.pval and logFC values obtained from MPNST vs. NF differential gene expression analysis with limma R package.

[3]Accession number from ArrayExpress database.

[4]Accession number from GEO database.

[5]Data included in GSE14038 accession.

The maximum adj.pval for genes with non-null score, for every study both in the gene signature and in the unfiltered list, was lower than the commonly used 0.05 to select differentially expressed genes, indicating that score selected genes were significantly

up- or downregulated, respectively, in every individual study (Table A). However, as columns max(logFC) and min(logFC) show, gene scores s_{ij} did not discriminate for high absolute logFC values. Due to the last filter step, these logFC values resulted slightly higher in the gene signature compared to the unfiltered gene list.

Results C: Genes included in main functional pathways associated to the MPNST vs. NF signature

Most upregulated genes were implicated in DNA replication and cell cycle, which are the main pathways involved in cell proliferation. Replication pathway concentrated genes involved in initiation of replication (CDC6, CDC45, CDT1, DBF4, GINS1, GINS2, MCM2, MCM4, MCM6), in elongation (RFC4), in sealing of nicks (LIG1), in control of replication (GMNN, PCNA), and in providing precursors for DNA synthesis (RRM2, and TYMS, targets of a cyclin-dependent kinase inhibitor [1], and of the chemotherapeutic agent 5-fluorouracil [2], respectively, in colorectal cancer). AURKA, AURKB, BIRC5 and CDCA8 are mitotic genes, members of the chromosomal passenger (CPC) complex, essential for alignment and segregation of chromosomes; PTTG1 regulates chromosome stability; SPDL1 and centromere proteins CENPA, CENPE, CENPF, CENPH, CENPI, CENPK, CENPN and CENPU were involved in protein recruitment to the centromere and kinetochore, mitotic progression and chromosome segregation; CASC5, KNTC1, MAD2L1, ZWINT, ZWILCH, SKA1, and NDC80 kinetochore complex components NDC80, NUF2, SPC24, SPC25, were required for chromosome segregation and spindle checkpoint activity; BUB1, BUB1B, CDK2, CHEK1, CKS1B, NEK2 appeared as mitotic checkpoint kinases, whereas PLK4 kinase regulates centriole duplication; CCNA2, CCNB1 and CCNB2 are cyclins that bind and activate mitotic kinases to promote cell cycle transitions; CDC20 and UBE2C allow cell cycle progression by destruction of mitotic cyclins; FBXO5 and SKP2 are involved in proteasome degradation of target proteins; chromosome cohesion was due to RAD21, SGOL1/SGO1 and, in meiosis, SGOL2/SGO2; NCAPG, NCAPH, NCAPD2, NCAPG2 and SMC4 are members of the condensin complex; and KIF18A, KIF18B, KIF20A, KIF23, KIF2C, and ECT2 take part in the kinesin complex. The KEGG extracellular matrix (ECM)-receptor pathway contains the potent apoptosis suppressor COMP, the motility receptor HMMR, the adhesion integrin ITGA4, and the osteopontin SPP1, relevant in interactions between the cell and the ECM. The KEGG ECM-receptor pathway is essential for cell maintenance and tissue and organ morphogenesis, involves fibril forming collagen from connective and cartilaginous tissues. We found that some genes in this pathway were shared with the Reactome pathway of

interactions with the neural adhesion immunoglobulin NCAM1.

Downregulated genes included in the immunity pathway related with complement activation incorporated regulatory elements of that pathway (CD55, A2M, PROS1, SERPING1 and CD59) in addition to complement components (C1S, C3, C4A, C4B and CFD). KEGG pathway of cell adhesion molecules (CAMs) groups glycoproteins located in the cell surface: integrins such as ITGA6 and ITGB8; immunoglobulins such as JAM2, CD58 and others related with the nervous system, such as L1CAM, the synaptic and potential tumor supressor CADM1, the brain specific receptor CADM3, the neuronal growth regulator NEGR1; selectins such as SELPLG; NLGN3, a neuroligin; MPZ, the main structural component of the peripheral myelin sheath; and cadherins CDH19 and PCDH20. Down-regulated genes also showed over-representation of circulating particles released into the extracellular space (blood microparticles).

Results D: Characterization of additional NF1-related gene signatures

In addition to the MPNST vs. NF comparison meta-analysis, we carried out other five comparisons: 2 meta-analyses to integrate nerve tumor tissue from human and mouse in comparisons NF vs. control and MPNST vs. control; 1 meta-analysis that combines human cell cultures to compare MPNST vs. control (MPNST cell lines vs. NHSC); 2 differential analyses from human cultured cells in NF vs. control (primary NFSC vs. NHSC) and MPNST vs. NF (MPNST cell lines vs. primary NFSC) comparisons. The number of genes that exhibited non-null scores in all comparisons of NF1-related signatures, both in cell cultures and in tumor tissue, is shown in Table B. The comparison MPNST vs. control showed the highest number of changes in gene expression in both types of tissue. The comparison NF vs. control showed the lowest amount of changes, especially in cultured cells.

Table B: Number of genes included in Neurofibromatosis gene signatures.

Tissue	Organism	Gene list	NF vs. control			MPNST vs. control			MPNST vs. NF		
			Up-regulated (score >0)	Down-regulated (score < 0)	Total	Up-regulated (score > 0)	Down-regulated (score < 0)	Total	Up-regulated (score >0)	Down-regulated (score < 0)	Total
Cell cultures	Human	Unfiltered list[1]	623	533	1156	4486	3900	8386	1591	1342	2933
		Gene signature[2]	63	54	117	449	390	839	160	135	295
Nerve tumors	Human/ Mouse	Unfiltered list[1]	3687	2892	6579	5204	3602	8806	4059	3005	7064
		Gene signature[2]	369	290	659	521	363	884	336	243	579

[1]Complete lists included in S2 Table.

[2]Gene lists bold-highlighted in S2 Table .

In tumor tissues, the MPNST vs. NF signature shared more genes with the gene signature MPNST vs. control than with the NF vs. control. Respective 67 and 36% of up- and downregulated genes from comparison MPNST vs. NF behaved similarly in MPNST vs. control, *i.e.* the score sign was identical in both comparisons. There were no genes showing opposite behavior, *i.e.* upregulated genes in one comparison being downregulated in the other one. Similar percentages of 66 and 48% were obtained comparing up- and downregulated genes from unfiltered lists of MPNST vs. NF and MPNST vs. control. In this case, a small percentage of 4 and 6% of up- and downregulated genes in MPNST vs. NF list showed score of opposite sign in MPNST vs. control gene list. In contrast, only 3 and 5% of up- and downregulated genes from MPNST vs. NF signature (25 and 20% in the unfiltered gene lists), respectively, appeared included in NF vs. control gene signature, indicating a lower similarity between these two signatures. Moreover, 1 and 5% of up- and downregulated genes, respectively, in the MPNST vs. NF signature exhibited opposite behavior in the NF vs. control signature. The amount of genes that showed opposite behavior in respective unfiltered gene lists increased to around 10 and 20%. The two additional tumor tissue gene signatures obtained from comparisons MPNST vs. control and NF vs. control also shared a quite large amount of genes. 23 and 38% of genes from the MPNST vs. control signature were also up- and down- regulated, respectively, in the NF vs. control gene signature. These percentages increased to 48 and 59% when we compared the unfiltered gene lists.

Compared to tumor tissue, the MPNST vs. NF signature shared a similar high number

of genes with the MPNST vs. control comparison in cell cultures. 56 and 77% of up- and down- regulated genes, respectively, from the MPNST vs. NF signature showed the same behavior in the MPNST vs. control comparison. There were no gene scores with opposite sign. Analogously, percentages 75 and 81 were computed for the unfiltered gene lists. Less than 1% of genes from MPNST vs. NF signature had scores with opposite sign in MPNST vs. control comparison. In contrast, and in agreement with tumor tissues, gene signatures from comparisons MPNST vs. NF and NF vs. control showed low similarity in cell cultures. Only 1 and 3% (4 and 13% comparing unfiltered gene lists) of respective up- and downregulated genes from MPNST vs. NF signature behaved in the same way in NF vs. control gene signature. Concerning cell culture comparisons MPNST vs. control and NF vs. control, most part of the small NF vs. control gene signature was included in the MPNST vs. control signature (60 and 59% of up and down- regulated genes, respectively). Higher percentages were observed for unfiltered signatures (68 and 83%, respectively).

To further examine similarities and differences between tumor tissue and cell cultures, we compared independently gene signatures from each comparison (MPNST vs. NF, MPNST vs. control and NF vs. control) in both types of tissue. High differences were observed between tumor tissue and cell cultures. Tumor tissue comparisons MPNST vs. NF, MPNST vs. control and NF vs. control only shared 12, 18 and 3% of genes with their respective cultured cell signatures. Although these percentages arose to 21, 42 and 6% in unfiltered gene lists, the amount of genes showing opposite behavior also increased between 3 and 5 times. Differences associated to the nature of the tissue probably contributed to such big different behavior between tumor tissue and cultured cells.

Due to the differences between cultured cells and tumor tissue in terms of gene expression, we expected analogous differences in the functional characterization of these gene signatures in the three comparisons considered: MPNST vs. NF, MPNST vs. control and NF vs. control. S14 Table summarizes biological process GO term enrichment for up- and down- regulated genes of each comparison.

As anticipated, a lot of similarities between GO terms associated to upregulated genes from MPNST vs. NF and MPNST vs. control were observed in tumor tissue. The MPNST vs. control signature showed a high number of GO terms associated to cell proliferation (52%), as well as morphogenesis and development of several systems according specification patterns, particularly nervous, skeletal, gastric, urogenital,

reproductive systems, hearth, and sense organs. Other GO terms were related to transcriptional activity and processes involved in malignancy such as collagen catabolic process, epithelial cell differentiation and cell migration (Table C in S14 Table). Unlike the MPNST vs. NF downregulated gene signature, no immunity GO terms associated to the MPNST vs. control downregulated genes were observed. This difference was still maintained when we compare the unfiltered gene lists. Instead, in common with the MPNST vs. NF signature, downregulated genes from MPNST vs. control comparison also seemed to control the peripheral nervous system development. Terms related to muscle system organization, regulation of membrane potential and regulation of phosphatidylinositol 3-kinase signaling also occurred (Table D in S14 Table).

The same differences and similarities observed between cultured cells and tumor tissue in the MPNST vs. NF comparison were observed in the MPNST vs. control comparison. GO terms related with cell proliferation from tumor tissue signatures for MPNST vs. NF and MPNST vs. control were nearly absent from signatures for these comparisons in culture cells. This difference was not observed when we evaluated the unfiltered gene lists for these two comparisons, which suggested lower scores for cell proliferation genes in cultured cells than in tumor tissue. In contrast, immune response positively associated to downregulated genes was observed for tumor tissue and cultured cells of MPNST vs. NF, but only for cultured cells of MPNST vs. control and not in the tumor tissue comparison. This difference was maintained when we compared unfiltered gene lists. In turn, many terms involving development, signal transduction, cell communication and migration can also be observed in MPNST vs. control gene signature (Table E in S14 Table). In MPNST vs. control downregulated genes, several GO terms associated to acquired immune response, development of peripheral nervous system, ECM organization and cell migration were over-represented (Table F in S14 Table).

Finally, the biggest difference in GO term representation was seen between comparisons MPNST vs. NF and NF vs. control. In tumor tissue NF vs. control comparison, most GO terms associated to upregulated genes were related with synaptic transmission (Ca^{2+} transport, membrane potential and synaptic exocytosis) (Table G in S14 Table). However, GO terms associated to cultured cells were related to growth and development, including branching morphogenesis of epithelial tubes and regulation of developmental bone morphogenetic (BMP) signaling pathway. Regulation of epithelial and mesenchymal cell proliferation, and ECM organization were also over-represented in GO terms associated to upregulated genes from cultured cells of NF vs.

control comparison (Table I in S14 Table). Considering the unfiltered lists of upregulated genes, several GO terms associated to innate immune response can be observed in NF vs. control comparison for tumor tissue and cultured cells. Instead, axonogenesis was the unique GO term linked to downregulated genes.

Results E: Homogeneity of gene profiles in each SOTA cluster

To check homogeneity of gene patterns in each cluster, Fig B illustrates the particular behavior of each gene in SOTA clusters.

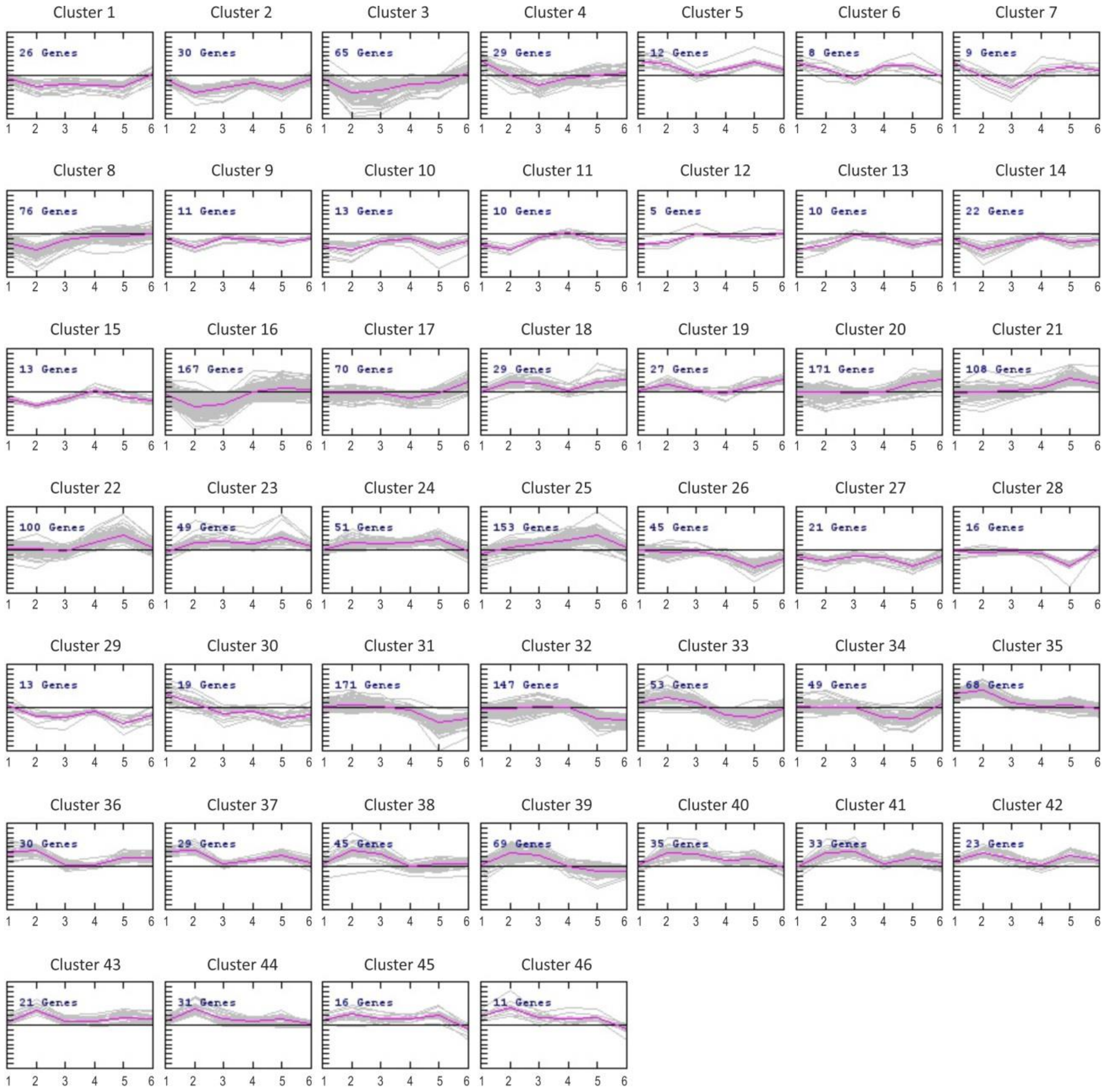

Fig B: Gene profiles in the SOTA clusters. Graphs from logFC_m values of genes grouped through SOTA in 46 clusters. Samples 1, 2 and 3 represent cell culture comparisons NF vs. control, MPNST vs. control and MPNST vs. NF, respectively. Samples 4, 5 and 6 represent nerve tumor comparisons MPNST vs. NF, MPNST vs. control and NF vs. control, respectively. Pink lines represent cluster centroids.

Although in general gene profiles fairly fit centroid vectors, some of the most populated clusters 16, 20-23 and 31 showed gene profiles that slightly differed from consensus centroids. The landscape of cluster profiles shows similarity among certain profiles according with their proximity in the SOTA tree. Additionally, these profiles remark the lack of symmetry in cell cultures regarding nerve tumors, particularly in clusters 7, 8, 16, 17, 20-22, 25, 26, 28, 30-35, 38 and 39. Cluster 33 presented the highest asymmetry between cell cultures and nerve tumor comparisons because most of genes were upregulated in cell cultures and downregulated in nerve tumors.

Discussion A: Comparison between over-represented chromosome regions in the MPNST vs. NF gene signature and previously described MPNST aberrant chromosome modifications

In order to correlate gene expression with gain or loss of chromosomic regions, we included as attribute for each gene the encoding chromosomic region. Despite differences reported in genomic aberrations (reviewed by Yang and Du [3]), our results corroborated the enrichment in chromosome regions previously described. Typical gain regions located in chromosome arms 7p, 8q and 17q, and we have found these regions over-represented, containing upregulated genes of the MPNST vs. NF gene signature and of the unfiltered list. In addition, we also identified gene enrichment in chromosome arm 4q, proposed as over-represented region [4]. The chromosome arm 15q [5], also reported as gene enriched, resulted only over-represented when we considered the unfiltered gene list, which indicates that genes included in this arm showed lower scores. Nevertheless, the over-representation of three bands from chromosome arm 15q illustrates the relevance of this region including cell proliferation genes, particularly four from the top 20 upregulated genes *(KIAA0101, NUSAP1, PRC1* and *CCNB2).* Over-representation of chromosome bands from the distal part of chromosome arm 17q included *TOP2A, BIRC5* and *TK1* genes. As this region was gained in five patients with poor outcome, a prognostic value has been associated to this amplification [6,7]. Besides these genes, *FOXM1, LOXL2* and *EYA4* were also described as upregulated and included in MPNST aberrant amplified genomic regions, in agreement with our results. In contrast with their over-expression in the gene signature, *HMMR* and *MMP13* were described as included in significant deleted chromosome regions (S11 Table).

We identified chromosome arm 3p over-represented for downregulated genes with the lowest cumulative probability and, with increasing probability, chromosome arms 1p, 5q, and 11q. Chromosome arms 1p, 3p and 11q also showed gene enrichment in the unfiltered downregulated gene list. Whereas chromosome arm 1p was reported as included in significant deleted regions, 3p and 11q were not previously described. Chromosome arm 3p includes a heterogeneous set of genes mainly involved in cell adhesion, cell communication, immune response, nervous system development and axon guidance. Some of them are related with cancer, particularly encoding genes for the Ser/Thr kinase TGFBR2 and the metalloproteinase inhibitor TIMP4. Chromosome arm 11q includes three of the 20 bottom signature genes *(PLEKHB1, CRYAB* and

ENDOD1). Unlike MPNST vs. NF gene signature, the whole unfiltered list also showed over-representation of 9p and 17p chromosome arms. Over-representation of band 17p13.1 for downregulated genes agreed with the MPNST deletion of this region encoding the tumor suppressor TP53 [8,9]. In turn, we did not find either over-representation among downregulated genes of band 9p21, the most frequently deleted region in MPNST [10]. In fact, *CDKN2A* and *CDKN2B,* located in that region, were only slightly downregulated in the unfiltered gene list. Considering reported downregulated genes related with NF and/or MPNST (S12 Table), in agreement with its downregulation, *RASSF2* is located in a significantly deleted region, whereas ITGB4, although downregulated, it locates in a MPNST amplified region, the distal part of chromosome arm 17q. *NKAIN2* and *RABGAP1L*, unexpectedly, were associated to deleted chromosomal regions in cutaneous NF1 neurofibromas.

Discussion B: Expression profile differences between cultured cells and nerve tumors observed in *SOX9*, *SUZ12*, *EGFR*, *SPP1* and *BMP2* genes

SOX9 was highly upregulated in cell culture comparisons whereas only a slight and moderate upregulation was seen in MPNST vs. NF and MPNST vs. control comparisons (S8 Table). In fact, the chosen score threshold of MPNST vs. NF gene signature avoided the inclusion of *SOX9* in this signature. In agreement with our results, Kolberg et al., [6] were unable to find upregulation for *SOX9*. *SUZ12*, the gene encoding one of the members of the silencing epigenetic complex PRC2, that in agreement with De Raedt et al. [11] was downregulated in the comparison MPNST vs. NHSC, did not show downregulation in tumor tissue. The gene encoding the epidermal growth factor receptor EGFR, reported to be amplified and upregulated in MPNST in several studies [12–14], was clearly upregulated in our data in the three comparisons from cell cultures, but it only showed to be upregulated in MPNST vs. control comparison in tumor tissue. Again, in agreement with Kolberg et al. [6], *EGFR* was not upregulated in the MPNST vs. NF comparison in tumor tissue. More paradoxical resulted the encoding gene for the osteopontin SPP1, that in agreement with Thomas et al. [15], resulted upregulated in the comparison MPNST vs. NF of tumor tissue, but it showed to be downregulated in cell cultures. In the unfiltered gene lists (Table J in S2 Table), the gene encoding the bone morphogenetic protein BMP2 was differentially up-regulated in MPNST cultured cells compared to control cells [16], but it was excluded from that respective gene signature due to the score threshold. In contrast with cultured cells, *BMP2* did not show upregulation in tumor MPNST vs. NF comparison. All these comparisons between our results and others previously reported suggest that some

differences in gene expression, imputed to intrinsic variation among tumors, might be due to the nature of tissue.

Discussion C: Panel of genes potentially silenced by hypermethylation of their CpG-island promoter region

In addition to RASSF1, other proteins seem to contribute to control the cell cycle arrest in different ways (S10 Table): the Ras oncogene family member RAB40B, involved in proteasomal degradation of target proteins; the cell growth suppressor FAM107A; the transcriptional repressor FOXS1; RHBDF1, the indirect activator of EGFR, that regulates cell survival, proliferation and migration; the mediator of cell survival NGFR; the regulator of cell cycle progression MX2; the proto-oncogene FGR, that negatively regulates cell migration and adhesion; INPP5D, the negative regulator of proliferation and survival of myeloid cells; the anti-apoptotic STAT6, an important diagnostic marker for solitary fibrous tumor [17]; the tumor suppressor CADM4; and the regulator of cell survival and apoptosis TRAF1.

As regulators of immune responses, S10 Table includes the GTPase activating protein RAP1GAP2, FGR, the proto-oncogene HCK, the arrestin ARRB1, the atypical chemokine receptor ACKR1, and the marker for neural crest Schwann cell lineage S100B. The EF-hand binding protein S100B is involved in intra and extracellular activities as regulator and signal [18]. S100B is downregulated in astrocytes by the epidermal growth factor EGF and the pro-inflammatory cytokine interferon (IFN)-γ [19]. It shows a complex transcriptional regulation that has not been addressed in the MPNST progression. S100B regulates Schwann cell proliferation and myelination with its co-regulator SOX10, described as hypermethylated [20]. However, due to our restrict threshold conditions, SOX10 was only found differentially hypermethylated in MPNST vs. control comparison. Besides S100B, other proteins involved in nervous system development and function are the signal transduction phosphatase inhibitor PPP1R1B, the nerve growth factor receptor NGFR, the scaffolding protein GRASP, a linker of receptors for phosphoinositides to neuronal proteins, the Ras protein RASGRF2, the transcription factor SOX8, that has also been related with some types of cancer [21], the axon outgrowth suppressor SLITRK2, the regulator of calcineurin RCAN1, and several proteins involved in structural functions such as PRIMA1, required to anchor acetylcholinesterase to the membrane of neuronal synapses in brain, the sialyltransferase ST6GALNAC2, the integrin ITGB4, and the myelin components MBP and MPZ.

In addition to genes *MBP, RABGAP1L, MPZ, ITGB4* and *S100B* (S12 Table), genes *PPP1R1B, FGR, NGFR* and *INPP5D* were related with MPNST and/or NF. PPP1R1B is a target of hippocampal dopamine. Reduction of dopamine levels thus decreased the PP1R1B phosphorylation levels in NF1 male mice [22]. FGR was not hyperexpressed in a neurofibrosarcoma compared to control tissue of a NF1 patient [23]. NGFR is a Schwann cell differentiation marker, downregulated in MPNST cell lines vs. control Schwann cells [24], and functional in NF cells [25]. NGFR acts as tumor suppressor that controls survival and death of neural cells, axonal growth and synaptic plasticity [26–29]. Its expression has been detected in human cancers of thyroid, stomach and liver. The NGFR hypermethylation status has been associated with cell proliferation, invasion, formation of colonies, and induced cell apoptosis in human colorectal cancer. NGFR silencing in this type of cancer reduced overall survival and disease-free survival [30]. Also in agreement with our results, loss of genomic region containing INPP5D seemed to cause silencing of INPP5D in cutaneous neurofibromas.

Not only downregulated genes resulted hypermethylated, but also many upregulated genes. Promoters of *KRT18, MEST, and WT1* genes were differentially hypermethylated in comparisons MPNST vs. NF and MPNST vs. control Schwann cells. These genes showed a similar pattern of gene upregulation. In agreement with our results, keratin KRT18 was reported as upregulated [6,31]. KRT18 is a biomarker for clinical diagnosis of cancer [32], although it has not been related with hypermethylation. In contrast, the imprinted genes *MEST* (mesoderm specific transcript) and the transcription factor *WT1 (*Wilms tumor) were related with hypermethylation. The modification in methylation levels at regulatory regions of imprinted genes, that exhibit preferential expression from a paternal allele, has been related with cancer. Aberrant methylation of *MEST* was associated to cervical cancer [33]. Upregulated in several cancers, *WT1* acts as an oncogene rather than a tumor suppressor [34] and aberrant hypermethylation of this gene was reported in hepatocellular carcinoma [35].

Discussion D: HDAC inhibitors counteract repression of CBX7 and over-expression of EZH2

The tightly regulation of cell migration and invasion by silencing CDH1 not only depends on HMGA2 and GSN, but also on the epigenetic Polycomb Repressor Complexes PRC1 and PRC2. Protein subunits of PRC1 and PRC2 control

developmental programs, whose deregulation drives to develop various types of cancer. PRC1 and PRC2, involved in epigenetic regulation of transcription by chromatin remodeling, act as transcriptional repressors by histone modification in two sequential steps. The first step involves trimethylation of histone H3 by PRC2; in the second step, PRC1 monoubiquitinates histone H2A [36]. PRC1 and PRC2 cooperate to condensate chromatin because trimethylated histones seem to recruit PRC1 to the promoter of target genes and, in addition, PRC1 and PRC2 show coordinate regulation in prostate cancer through microRNAs [37]. In MPNST some protein subunits from both PRC1 and PRC2 were differentially expressed and contribute to the inactivation of these two fundamental epigenetic regulators. The presence among top 20 downregulated genes in MPNST vs. NF signature of encoding gene for CBX7, the histone chromatin remodeling enzyme, component of the epigenetic repressor complex PRC1, suggested that PRC1 could also play a role on epigenetic regulation, probably impaired during NF malignant transformation. Our results also suggest dysfunction of PRC2 due to the over-expression of EZH2, subunit of PRC2. Loss of PRC2 has a favorable effect on transcription of genes from the Ras signaling pathway [11]. *EZH2* upregulation and *CBX7* downregulation are reported in breast tumors [36].

CBX7, a protein subunit of PRC1 is silenced in several cancers and proposed for evaluation as prognostic marker [38]. CBX7 is involved in the regulation of cell cycle and proliferation genes [39]. The loss of expression of *CBX7* drives to poor prognosis and progression to malignancy due to its essential role in epithelial to mesenchymal transition (EMT) [38]. Because CBX7 inhibits the silencing activity of HDAC2 over the *CDH1* promoter, the downregulation of CBX7 drives to EMT and to the malignant phenotype [40], not only by its lack of binding to *CDH1* promoter, but also by the lack of repression of the osteopontin gene *SPP1* (rank position 141 in Table A in S2 Table) and other genes associated with cell migration and invasion [41,42]. HDAC inhibitors avoid the silencing effect of HDAC2 over *CDH1* promoter, neutralizing the silencing of CBX7 and restoring expression levels of CDH1, which contributes to avoid metastasis. Being regulated by a member of the HMGA protein family, *CBX7* could also be an indirect target of HDAC inhibitors [38].

The upregulation of the PRC2 protein component EZH2 (rank position 114 in Table A in S2 Table), has also been reported to promote the invasive phenotype by inhibiting the expression of CDH1 by histone methylation at the *CDH1* promoter region. TSA and vorinostat HDAC inhibitors would reverse CDH1 silencing by reducing the PRC2 occupancy on the *CDH1* promoter, attenuating the metastatic phenotype [43]. PRC2

inactivation depends on co-deletion/mutation of *NF1* and *SUZ12* and/or *EED*, genes encoding two subunits of PRC2 [11]. Whereas *SUZ12* and *EED* were not significantly deregulated in MPNST vs. NF gene signature, *EZH2* appeared highly induced. This contradictory upregulation of EZH2, reported in multiple types of cancers, and correlated with poor prognosis, may be explained by a oncogenic activity of EZH2 independent of its transcriptional repression function [44], or as a consequence of the high cell proliferation rate rather than a cause of that proliferation [45]. In fact, *EZH2* expression correlated with the expression of the proliferation marker MKI67, ranking in position 32 in the gene signature. Consequently, the inhibition of EZH2 has shown antitumor effects in MPNST [46]. In agreement with De Raedt *et al.* [11], at least in MPNST showing co-deletion/mutation of *SUZ12* and *EED,* the use of EZH2 inhibitors as therapeutic agents could exacerbate the malignant phenotype. However, TSA and vorinostat HDAC inhibitors would prevent the activity of EZH2 without disturbing PRC2 protein-protein interactions and without altering the expression of PRC2 members, including HDAC1 [43].

Discussion E: Supplementation with acetate precursors as coadjuvant chemotherapy

The encoding gene for the aspartatoacylase ASPA, which metabolizes N-acetyl-L-aspartic acid (NAA) to aspartate and acetate, ranks in position 10 among negative score genes from MPNST vs. NF gene signature. Although ASPA protein is better known by its role in myelin lipid biosynthesis in central nervous system, essential to maintain intact the white matter, it is also involved in neuroblastoma, a cancer of the sympathetic nervous system in which low expression correlates with poor prognosis [47]. It is downregulated at both mRNA and protein levels in esophageal squamous cell carcinoma, glioma and glioblastoma [48–50]. The metabolic reprogramming due to the lack of ASPA causes the starvation of its final product, acetate, the essential precursor for oligodendrocyte myelination and histone acetylation. This metabolic inhibition of histone acetylation induces tumor cell transformation. The supplementation with an external source of acetate could solve the metabolic problem. In fact, a preclinical study testing the supplementation of glycerol triacetate (GTA) as a chemotherapeutic coadjuvant has been reported in glioma [51]. GTA is hydrophobic and able to cross the blood-brain barrier and plasma membranes. The cytostatic growth arrest caused by GTA was similar to the growth arrest obtained with the HDAC inhibitor vorinostat. However, this growth arrest was not associated with apoptosis or differentiation, but to the increased acetylation of proteins involved in cell cycle regulation [52].

In addition to ASPA, the enzyme ADH1B could be de-regulated in the provision of acetate. It ranks in position 7 among negative gene scores of the MPNST vs. NF signature. This enzyme catalyzes the conversion of alcohol in acetaldehyde. In a second step of alcohol degradation, the aldehyde dehydrogenase ALDH2 oxidizes acetaldehyde to acetate. Although the upregulation of ADH1B has been associated with poor prognosis in high-grade serous ovarian cancer [53], it was downregulated in tongue squamous cell carcinoma [54], and appeared upregulated or downregulated at later stages of fetal lung development [55]. The lack of ADH1B in MPNST points to a similar role as ASPA to generate acetylation precursors. Then, supplementation with GTA as coadjuvant chemotherapy could complement two metabolic pathways involved in the synthesis of acetate, involving ASPA and ADH1B. The required experimental confirmation is indispensable to validate this hypothesis.

Materials and methods A: Microarray data pre-processing

Affymetrix platform data were normalized by the function rma from affy package [56] that uses the robust multi-array average (RMA) expression measure. We normalized Agilent platform data within and between arrays by methods loess and quantiles by using the limma R package [57]. After normalization, R and G normalized values were retrieved from MA matrix values to obtain a normalized expression set, including reference values of the two color platform by using the R conversion formula `R=2^((1/2)*(M+2A)); G=2^((1/2)*(-M+2A))`. ABI platform data was normalized by quantiles through the function qnNormalize from ABarray package [58]. When sample batch information was provided in the experiment, we adjusted for batch effects the expression set obtained for each study by the function ComBat of sva package [59] by using the R code:

```
###Exprs set batch adjustment
library(sva)
Batch = targets$batch
mod = model.matrix(~as.factor(phenotype), data=targets)
Exprs_adjusted = ComBat(dat=Exprs_0, batch=Batch, mod=mod,
par.prior=TRUE, prior.plots=FALSE)
```

The resulting expression set was filtered twice to remove features showing little variation. The first filter ruled out features with expression values with standard deviation lower than quantile 0.2 by varFilter function from genefilter package [60] or a similar R script:

```
###Filtering
library(genefilter)
esetIQR <- varFilter(eset, var.func=sd, var.cutoff=0.2,
filterByQuantile=TRUE)
Exprs_filtered<-exprs(esetIQR)
###or
Exprs_filtered<-matrix(nrow= nrow, ncol= 1, apply(Exprs, 1, sd))
rownames(Exprs_filtered)<-rownames(Exprs)
Exprs_filtered<- Exprs [rownames(as.matrix(Exprs_filtered
[Exprs_filtered [,1] > quantile (Exprs_filtered [,1], 0.2),])), ]
```

R code to discard features with FDR values >0.05 obtained by ANOVA in the limma package [57]:

```
library(limma)
phenotypes<-factor(colnames(Exprs_filtered))
aof<-function(x){m<-data.frame(phenotypes,x);anova(aov(x~phenotypes,m))}
anovaresults<-apply(Exprs_filtered, 1, aof)
pvalues<-data.frame(lapply(anovaresults,function(x){x["Pr(>F)"][1,]}))
tpvalues<-t(pvalues)
colnames(tpvalues)<-"pvalue"
fdr.result<-p.adjust(tpvalues[,1], "BH")
bhtresh<-cbind(tpvalues, fdr.result)
order_bhtresh<-bhtresh[order(fdr.result),]
row.names(order_bhtresh)<-
substring(row.names(order_bhtresh),2,nchar(row.names(order_bhtresh)))
```

```
Exprs_filtered_anova<- Exprs_filtered [rownames(Exprs_filtered) %in%
rownames(order_bhtresh[order_bhtresh[,2] < 0.05,]),]
```

R code used to analyze principal components (PCA):

```
Exprs_2<- Exprs_filtered_anova
Exprs_2_t<-t(Exprs_2)
Exprs_2_t_out=prcomp(Exprs_2_t,scale=TRUE)
Cols = function(vec) {
       cols = rainbow(length(unique(vec)))
       return(cols[as.numeric(as.factor(vec))])
       }
###plot in two dimensions
par(mfrow = c(1,2))
plot(Exprs_2_t_out$x[,1:2],col = Cols(classes),pch =19,
       xlab ="PC1",ylab ="PC2")
plot(Exprs_2_t_out$x[,c(1,3)], col = Cols(classes),pch =19,
       xlab ="PC1",ylab ="PC3")
```

R code to determine differentially expressed genes with the limma package [57]:

```
library(limma)
phenotypes<-factor(colnames(Exprs_2))
###Levels: Phenotype1   Phenotype2   Phenotype3
design<-model.matrix(~0+phenotypes)  #or#
design<- modelMatrix(targets,ref="Universal_RNA")
colnames(design)<-levels(phenotypes)
fit <-lmFit(Exprs_2, design) #or# fit<-lmFit(MA, design)
cont.matrix<-makeContrasts("Phenotype1vsPhenotype2"= Phenotype1  -
Phenotype2   , "Phenotype1vsPhenotype3"= Phenotype1-Phenotype3,
"Phenotype2vsPhenotype3"= Phenotype2-Phenotype3, levels=design)
fit2<-contrasts.fit(fit, cont.matrix)
fit2<-eBayes(fit2)

###Comparison Phenotype1vsPhenotype2
toptable_coef_Phenotype1vsPhenotype2<-toptable(fit2,coef="
Phenotype1vsPhenotype2", n=nrow(fit), adjust.method="BH")
```

Materials and methods B: Translation from probe names to human ENSEMBL gene IDs, HUGO IDs and mapping in human chromosome arms

R code to translate probe names of Affymetrix U133 Plus 2.0 platform (example) by using the biomaRt package ([61]:

```
library(biomaRt)
ensembl = useMart(biomart =
"ENSEMBL_MART_ENSEMBL",dataset="hsapiens_gene_ensembl", host =
"sep2015.archive.ensembl.org")
humanensembl<-useDataset("hsapiens_gene_ensembl", mart=ensembl)
probe_to_gene<-getBM(attributes = c("affy_hg_u133_plus_2",
"ensembl_gene_id","hgnc_symbol", "chromosome_name", "band"),
mart=humanensembl)
```

Materials and methods C: Scores of genes across studies and final score for each gene in a comparison between two phenotypes

The score s_{ij} for each gene (i) in each comparison study (j) was calculated modifying the previously proposed formula [62], as follows:

$$s_{ij} = \begin{cases} S(logFC\_m_{ij})\left(1 - \dfrac{MAD_{ij}}{|logFC\_m_{ij}|}\right)(1 - pval_{ij}) & pval_{ij} < 0.1,\ \ 1 - \dfrac{MAD_{ij}}{|logFC\_m_{ij}|} > 0,\ \ B > 0 \\ 0 & otherwise \end{cases}$$

$$S(logFC\_m_{ij})\ scales\ logFC\_m_{ij} > 0 \quad between\ 0\ and\ +1\ and$$
$$logFC\_m_{ij} < 0 \quad between\ -1\ and\ 0$$

$pval_{ij}$ and B_{ij} factors for each gene in each study were obtained from computation of the differential expression between two phenotypes by using the data expression sets and the limma R package [57]. MAD_{ij} is the median deviation of each ratio between two phenotypic samples to the $logFC\_m_{ij}$. The robust $logFC\_m_{ij}$ value was calculated as the log_2 median of all expression ratios among the samples of the phenotypes compared. Phenotype expression values were obtained as 2 raised to the expression value derived from each microarray normalization step. The normalization step of positive and negative $logFC\_m_{ij}$ values regarding $logFC\_m_{ij}$ quantiles 99.95% and 0.05%, respectively, yielded scaled $S(logFC\_m_{ij})$ values normalized between -1 and +1. Values of $logFC\_m_{ij}$ > or < than quantiles 99.95% and 0.05%, respectively, were mapped to 1 or -1, respectively. R code used to compute S(logFC_mij) values:

```
scaled_logFC_median<-numeric()
for(i in 1: nrow){
      scaled_logFC_median [i]<-ifelse(logFC_median[i]>0,
      logFC_median[i]/as.numeric(quantile(logFC_median, .9995)),
      logFC_median[i]/abs(as.numeric(quantile(logFC_median, 0.0005))))}
scaled_logFC_median<- ifelse(scaled_logFC_median > 1, 1,
scaled_logFC_median)
scaled_logFC_median<- ifelse(scaled_logFC_median < -1, -1,
scaled_logFC_median)
```

The multiplication of the three factors of the formula shown above gave a s_{ij} score value between -1 and +1, positive for upregulated genes and negative for downregulated genes. Unlike the previously described score, our score retained the sign of size effect distinguishing up- and downregulated genes across the analysis. After computing gene scores in each experiment, we tested the three constraints imposed to the calculation of scores, i.e. P-values <0.1 and penalization factors due to deviations of size effect and B factors >0. If these constraints were satisfied, gene scores retained their values. Otherwise, score values were coerced to zero. R code to compute the gene scores s_{ij}:

```
score<-numeric()
for(i in 1:nrow){score[i]<-
      scaled_logFC_median [i] * (1 - P.Value[i]) * (1 -
      (MAD[i]/abs(logFC_median[i]))) }

###Conditions for score
for(i in 1:nrow){score[i]<-
      ifelse((1 - (MAD[i]/abs(logFC_median[i]))) [i] < 0, 0, score[i])}
```

```
for(i in 1:nrow){score[i]<- ifelse(P.Value[i] > 0.1, 0, score[i])}
for(i in 1:nrow){score[i]<-ifelse(B[i] < 0, 0, score[i])}
```

Materials and methods D: Computation of bias in score values among studies: Bhattacharyya distance (BD) ratio

R code (apply for all genes):

```
###nplatform: number of independent studies
###s: final score
###s1: human score 1
###s2: human score 2
###s3: human score 3
###s4: human score 4
###sm: mouse score
###max_s: maximum score (excluding mouse data)
max_s <- max(c(abs(s1), abs(s2), abs(s3), abs(s4)), na.rm=T)
###max_b: maximum score (including mouse data)
max_b <- max(c(abs(s1), abs(s2), abs(s3), abs(s4), abs(sm)), na.rm=T)
###genename: gene name
###only_human: list of genes ignoring mouse data
###Gene Bhattacharya distance
Bhattacharyya_dist<-numeric()
Bhattacharyya_dist[i]<-
             ifelse(!is.na(s) & s !=0,
                   ifelse(nplatform > 0,
                        ifelse(genename %in% only_human,

       (-1)*log((1/nplatform)*(sum(c(sqrt(abs(s1)/max_s),sqrt(abs(s2)/max_s),
       sqrt(abs(s3)/max_s), sqrt(abs(s4)/max_s)), na.rm=T))),
       (-1)*log((1/nplatform)*(sum(c(sqrt(abs(s1)/max_b),  sqrt(abs(s2)/max_b),
       sqrt(abs(s3)/max_b),     sqrt(abs(s4)/max_b),     sqrt(abs(sm)/max_b)),
       na.rm=T)))), NA), NA)
###Gene DB maximum
max_BD<-numeric()
max_BD<-ifelse(nplatform==5,(-1)*log(1/5),
               ifelse(nplatform==4, (-1)*log(1/4),
                      ifelse(nplatform==3, (-1)*log(1/3),
                            ifelse(nplatform==2, (-1)*log(1/2),
                                   ifelse(nplatform==1, 0, NA)))))
###Gene BD_ratio
BD_ratio<-numeric()
BD_ratio<-ifelse(nplatform>1, (Bhattacharyya_dist *100)/max_DB,
                                      ifelse(nplatform==1, 0, NA))
```

Materials and methods E: DNA methylation analysis

SRA files were downloaded from GEO database and transformed to fasq format by the sra.toolkit program. The human genome assembly hg19 (GRCh37) used to align fastq sequences was downloaded from UCSC Genome Bioinformatics with the wget program. The index reference genome was created by the program bwa [63]. We aligned pair end sequences against the reference genome using the program bwa. Through the samtools program [64], we transformed sam files to bam files. Appropriate alignments of resulting bam files from the 6 replicates per phenotype were displayed with the Integrative Genomics Viewer (IGV; [65]) after creating an index for bam files. We inspected data by using the MEDIPS R package [66] obtaining Pearson correlations between all pairs of samples and calculating coverage saturation and calibration plots. Concerning Pearson correlation between all pairs of samples, MPNST samples correlated better among them (median 0.95) than NF and control phenotype samples (medians 0.87 and 0.89, respectively). Comparing pairs of phenotypes, high correlation was observed between NF and control samples (median 0.86) whereas these two phenotypes showed lower correlation with MPNST (median 0.65 between MPNST and NF, and median 0.59 between MPNST and control samples). To ensure that the covering of sequences along the whole genome was sufficient to compute differential DNA methylation between pairs of phenotypes, we applied a coverage saturation analysis to individual samples. As saturation plots show in Fig C, estimated correlation between artificial subsets of sequencing data was higher than 0.9 for every sample. MPNST samples showed estimated correlation values of 0.97; samples of NF exhibited 0.92 of estimated correlation; and 0.93 was the value for control replicates. These results guaranteed enough sequencing depth and allowed us to conclude that the selected window size of 200 was appropriate to accomplish the following analysis. The calibration plots shown in Fig D indicated that the normalization step regarding CpG density assured an effective MeDIP enrichment. We also compared the resulting bam files for differential methylation by MEDIPS package. We substituted stacked reads by only one representative to avoid false positives in the comparison of conditions. Translation of coordinates to promoters was carried out downloading coordinates from the UCSC Table Browser, selecting the human assembly Feb. 2009 (GRCh37/hg19), group Genes and Gene Predictions, track RefSeq Genes, BED output format, and get BED after selecting upstream by 1500 bases (promoter).

```
###DNA methylation analysis
###Download and reprocessing sra files(command line in linux)
       ###sra-toolkit program: download fastq files
```

```
	prefetch –v SRR0427XX fasq.dump –outdir/opt/fastq/ --split-
files/home/sra_files/SRR0427XX.sra
	###wget program: download reference genome hg19
	wget
http://hgdownload.cse.ucsc.edu/goldenPath/hg19/bigZips/chromFa.tar.gz
	###bwa program: reference genome index
	bwa index –p bwa index –p hg19bwaidx –a bwtsw hg19.fa
	###bwa program: alignment of individual sequences to reference genome
	bwa mem hg19bwaidx SRR0427XX_1.fastq SRR0427XX_2.fastq > SRR0427XX_aln-
pe.sam
	###samtools program:  sam files to bam files
	samtools view –bhuS SRR0427XX_aln-pe.sam | samtools sort –
SRR0427XX_sorted
	###samtools program: index for bam files
	samtools index SRR0427XX_aln-pe.bam


###Files in the R working directory:
#SRR042748_sorted.bam
#SRR042750_sorted.bam
#SRR042752_sorted.bam
#SRR042754_sorted.bam
#SRR042756_sorted.bam
#SRR042758_sorted.bam
#SRR042760_sorted.bam
#SRR042762_sorted.bam
#SRR042764_sorted.bam
#SRR042766_sorted.bam
#SRR042768_sorted.bam
#SRR042770_sorted.bam
#SRR042772_sorted.bam
#SRR042774_sorted.bam
#SRR042776_sorted.bam
#SRR042778_sorted.bam
#SRR042780_sorted.bam
#SRR042782_sorted.bam

###Read the input and create the MEDIPS sets for the three different
phenotypes (MPNST, NF and Normal)
library(MEDIPS)
library(BSgenome.Hsapiens.UCSC.hg19)
BSgenome="BSgenome.Hsapiens.UCSC.hg19"
uniq=1e-3
extend=300
shift=0
ws=200
bam_file_MPNST<-c("SRR042748_sorted.bam", "SRR042750_sorted.bam",
"SRR042752_sorted.bam", "SRR042754_sorted.bam", "SRR042756_sorted.bam",
"SRR042758_sorted.bam")
bam_file_NF<-c("SRR042760_sorted.bam",    "SRR042762_sorted.bam",
"SRR042764_sorted.bam",   "SRR042766_sorted.bam", "SRR042768_sorted.bam",
"SRR042770_sorted.bam")
bam_file_Normal<-c("SRR042772_sorted.bam",   "SRR042774_sorted.bam",
"SRR042776_sorted.bam",  "SRR042778_sorted.bam", "SRR042780_sorted.bam",
"SRR042780_sorted.bam", "SRR042782_sorted.bam")
MPNST_MeDIP = lapply(X=bam_file_MPNST, FUN= MEDIPS.createSet, BSgenome =
BSgenome, extend = extend, shift = shift, uniq = TRUE, window_size = ws)
NF_MeDIP = lapply(X=bam_file_NF, FUN= MEDIPS.createSet, BSgenome = BSgenome,
extend = extend, shift = shift, uniq = TRUE, window_size = ws)
Normal_MeDIP = lapply(X=bam_file_Normal, FUN= MEDIPS.createSet, BSgenome =
BSgenome, extend = extend, shift = shift, uniq = TRUE, window_size = ws)

###Local density of CpGs considering the genome and window parameters
CS_MPNST = MEDIPS.couplingVector(pattern = "CG", refObj = MPNST_MeDIP[[1]])

###Exploring data and quality control of samples: Obtaining Pearson
correlations between all pairs of samples
cor.matrix = MEDIPS.correlation(MSets=c(MPNST_MeDIP, NF_MeDIP, Normal_MeDIP),
```

```
plot =T, method ="pearson")

###Exploring data and quality control of samples: Calibration plots for MPNST
MeDIP set (calibration plots were obtained in a similar way for NF and Normal
MeDIP sets)
for (i in 1:length(MPNST_MeDIP)){
        png(paste("D:/working_directory/Suppl_Figures/MPNST",
"/Suppl.Fig1_calibration_MPNST_", i, ".png", sep=""))
        MEDIPS.plotCalibrationPlot(MSet=MPNST_MeDIP[[i]], CSet=CS_MPNST)
        dev.off()
}

###Exploring data and quality control of samples: Saturation analysis for
MPNST MeDIP set (saturation analyses were carried out in a similar way for NF
and Normal MeDIP sets)
for (i in 1:length(MPNST_MeDIP)){
  png(paste("D:/working_directory/Suppl_Figures/MPNST",
"/Suppl.Fig2_saturation_cancer_", i, ".png", sep=""))
  sr=MEDIPS.saturation(file=bam_file_MPNST[i], BSgenome = BSgenome, uniq =
TRUE, extend =extend, shift= shift, window_size = ws, nit = 10, nrit = 1,
empty_bins = TRUE, rank = FALSE)
  MEDIPS.plotSaturation(sr)
  dev.off()
}

###Differential methylation analysis between MPNST and NF (comparisons between
MPNST vs. Normal and NF vs. Normal were analyzed in a similar way)
results_MPNST_vs_NF = MEDIPS.meth(MSet1 = cancer_MeDIP, MSet2 = benign_MeDIP,
CSet = CS_cancer, p.adj = "bonferroni", diff.method = "edgeR",
                                  MeDIP = T, CNV = F, minRowSum = 10)


###Selecting significant windows for MPNST vs. NF comparison (similarly were
obtained significant windows for MPNST_vs_Normal and NF_vs_Normal):
mr.edgeR_MeDIP_MPNST_vs_NF.s = MEDIPS.selectSig(results = results_MPNST_vs_NF,
p.value = 0.1, adj = T, ratio = NULL, bg.counts = NULL, CNV = F)
###Merging hypermethylated regions
DMR_MPNST_vs_NF.s.gain =
mr.edgeR_MeDIP_MPNST_vs_NF.s[which(mr.edgeR_MeDIP_MPNST_vs_NF.s[,
grep("logFC", colnames(mr.edgeR_MeDIP_MPNST_vs_NF.s))] > 0), ]
DMR_MPNST_vs_NF.s.gain.m = MEDIPS.mergeFrames(frames = DMR_MPNST_vs_NF.s.gain,
distance = 1)
###Merging hypomethylated regions
DMR_MPNST_vs_NF.s.loss =
mr.edgeR_MeDIP_MPNST_vs_NF.s[which(mr.edgeR_MeDIP_MPNST_vs_NF.s[,
grep("logFC", colnames(mr.edgeR_MeDIP_MPNST_vs_NF.s))] < 0), ]
DMR_MPNST_vs_NF.s.loss.m = MEDIPS.mergeFrames(frames = DMR_MPNST_vs_NF.s.loss,
distance = 1)
###Extraction of data from regions of interest (ROIs)
columns = names(results_MPNST_vs_NF)[grep("counts|rpkm|edgeR",
names(results_MPNST_vs_NF))]
rois_MPNST_vs_NF.s.gain.m = MEDIPS.selectROIs(results = results_MPNST_vs_NF,
rois = DMR_MPNST_vs_NF.s.gain.m, columns = columns, summarize = "avg")
rois_MPNST_vs_NF.s.loss.m = MEDIPS.selectROIs(results = results_MPNST_vs_NF,
rois = DMR_MPNST_vs_NF.s.loss.m, columns = columns, summarize = "avg")




###Annotation
#Limiting hypermethylated ROIs to chromosomes 1 to 22 and X (analogously for
hypomethylated)
rois_MPNST_vs_NF.s.gain.m_select<-
rois_MPNST_vs_NF.s.gain.m[rois_MPNST_vs_NF.s.gain.m$chr=="chr1"|rois_MPNST_vs_
NF.s.gain.m$chr=="chr2"|

rois_MPNST_vs_NF.s.gain.m$chr=="chr3"|rois_MPNST_vs_NF.s.gain.m$chr=="chr4"|
```

```
rois_MPNST_vs_NF.s.gain.m$chr=="chr5"|rois_MPNST_vs_NF.s.gain.m$chr=="chr6"|

rois_MPNST_vs_NF.s.gain.m$chr=="chr7"|rois_MPNST_vs_NF.s.gain.m$chr=="chr8"|

rois_MPNST_vs_NF.s.gain.m$chr=="chr9"|rois_MPNST_vs_NF.s.gain.m$chr=="chr10"|

rois_MPNST_vs_NF.s.gain.m$chr=="chr11"|rois_MPNST_vs_NF.s.gain.m$chr=="chr12"|

rois_MPNST_vs_NF.s.gain.m$chr=="chr13"|rois_MPNST_vs_NF.s.gain.m$chr=="chr14"|

rois_MPNST_vs_NF.s.gain.m$chr=="chr15"|rois_MPNST_vs_NF.s.gain.m$chr=="chr16"|

rois_MPNST_vs_NF.s.gain.m$chr=="chr17"|rois_MPNST_vs_NF.s.gain.m$chr=="chr18"|

rois_MPNST_vs_NF.s.gain.m$chr=="chr19"|rois_MPNST_vs_NF.s.gain.m$chr=="chr20"|

rois_MPNST_vs_NF.s.gain.m$chr=="chr21"|rois_MPNST_vs_NF.s.gain.m$chr=="chr22"|

rois_MPNST_vs_NF.s.gain.m$chr=="chrX",]


###Promoter annotation
#File of coordinates from promoters download from UCSC Table Browser:
“"promoter_1500_hg19"
promoter_1500_hg19<-read.csv("promoter_1500_hg19", sep="\t", header=FALSE)
promoter_1500_hg19<-promoter_1500_hg19[,1:4]
colnames(promoter_1500_hg19)<-c("chr","start", "end","promoter")
aaa<-as.character()
for(i in 1:57111){aaa[i]<-gsub("_up_1500_chr(([0-9]+)|X)_([0-9]+)_[f-r]", "",
as.character(promoter_1500_hg19$promoter[i]))}
promoter_1500_hg19_df<-cbind(aaa, promoter_1500_hg19[,1:3])
colnames(promoter_1500_hg19_df)[1]<-"refseq_mrna"

#Adding RefSeq promoter annotation to ROIs (hypermethylated, analogously for
hypomethylated)
rois_MPNST_vs_NF.s.gain.m_anot = MEDIPS.setAnnotation(regions =
rois_MPNST_vs_NF.s.gain.m_select, annotation = promoter_1500_hg19_df)
id<-list()
for(i in 1:((length(colnames(rois_MPNST_vs_NF.s.gain.m_select))-36){id[[i]]<-
rois_MPNST_vs_NF.s.gain.m_anot[!is.na(rois_MPNST_vs_NF.s.gain.m_anot[,36+i]),c
(1:3, 32:36, 36+i)]
colnames(id[[i]])[9]<-"ENSPROMOTER"}
rois_MPNST_vs_NF.s.gain.m_anot_promoter <- do.call("rbind", id)

#Let unique values of edgeR.logFC and edgeR.logCPM for each RefSeq annotated
promoter (hypermethylated; analogously for hypomethylated)
groups<-split(rois_MPNST_vs_NF.s.gain.m_anot_promoter,
rois_MPNST_vs_NF.s.gain.m_anot_promoter[,9])
for(i in(1:length(groups))){groups[[i]]<-
groups[[i]][abs(groups[[i]]$edgeR.logFC)==max(abs(groups[[i]]$edgeR.logFC)),][
1,]}
rois_MPNST_vs_NF.s.gain.m_anot_promoter <- do.call("rbind", groups)
rois_MPNST_vs_NF.s.gain.m_anot_promoter<-
rois_MPNST_vs_NF.s.gain.m_anot_promoter[order(rois_MPNST_vs_NF.s.gain.m_anot_p
romoter$edgeR.adj.p.value, decreasing=F),]

#Translate RefSeq annotation to ENSEMBL annotation
library(biomaRt)
ensembl = useMart(biomart =
"ENSEMBL_MART_ENSEMBL",dataset="hsapiens_gene_ensembl", host =
"feb2014.archive.ensembl.org")
humanensembl<-useDataset("hsapiens_gene_ensembl", mart=ensembl)
humangenes_mrna<-getBM(attributes = c("ensembl_gene_id", "refseq_mrna",
"hgnc_symbol"), mart=humanensembl)
humangenes_ncrna<-getBM(attributes = c("ensembl_gene_id", "refseq_ncrna",
"hgnc_symbol"), mart=humanensembl)

colnames(humangenes_mrna)[2]<-"ENSPROMOTER"
```

```
colnames(humangenes_ncrna)[2]<-"ENSPROMOTER"

rois_MPNST_vs_NF.s.gain.m_anot_promoter_genes_1<-
merge(rois_MPNST_vs_NF.s.gain.m_anot_promoter, humangenes_mrna,
by="ENSPROMOTER")
rois_MPNST_vs_NF.s.gain.m_anot_promoter_genes_2<-
merge(rois_MPNST_vs_NF.s.gain.m_anot_promoter, humangenes_ncrna,
by="ENSPROMOTER")
rois_MPNST_vs_NF.s.gain.m_anot_promoter_genes<-
rbind(rois_MPNST_vs_NF.s.gain.m_anot_promoter_genes_1,
rois_MPNST_vs_NF.s.gain.m_anot_promoter_genes_2)
rois_MPNST_vs_NF.s.gain.m_anot_promoter_genes<-
rois_MPNST_vs_NF.s.gain.m_anot_promoter_genes[!is.na(rois_MPNST_vs_NF.s.gain.m
_anot_promoter_genes$ensembl_gene_id),]
#Let unique values of edgeR.logFC and edgeR.logCPM for each ENSEMBL annotated
promoter (hypermethylated; analogously for hypomethylated)
groups<-split(rois_MPNST_vs_NF.s.gain.m_anot_promoter_genes,
rois_MPNST_vs_NF.s.gain.m_anot_promoter_genes[,10])
for(i in(1:length(groups))){groups[[i]]<-
groups[[i]][abs(groups[[i]]$edgeR.logFC)==max(abs(groups[[i]]$edgeR.logFC)),][
1,]}
rois_MPNST_vs_NF.s.gain.m_anot_promoter_transl <- do.call("rbind", groups)
rois_MPNST_vs_NF.s.gain.m_anot_promoter_transl<-
rois_MPNST_vs_NF.s.gain.m_anot_promoter_transl[order(rois_MPNST_vs_NF.s.gain.m
_anot_promoter_transl$edgeR.adj.p.value, decreasing=F),]
```

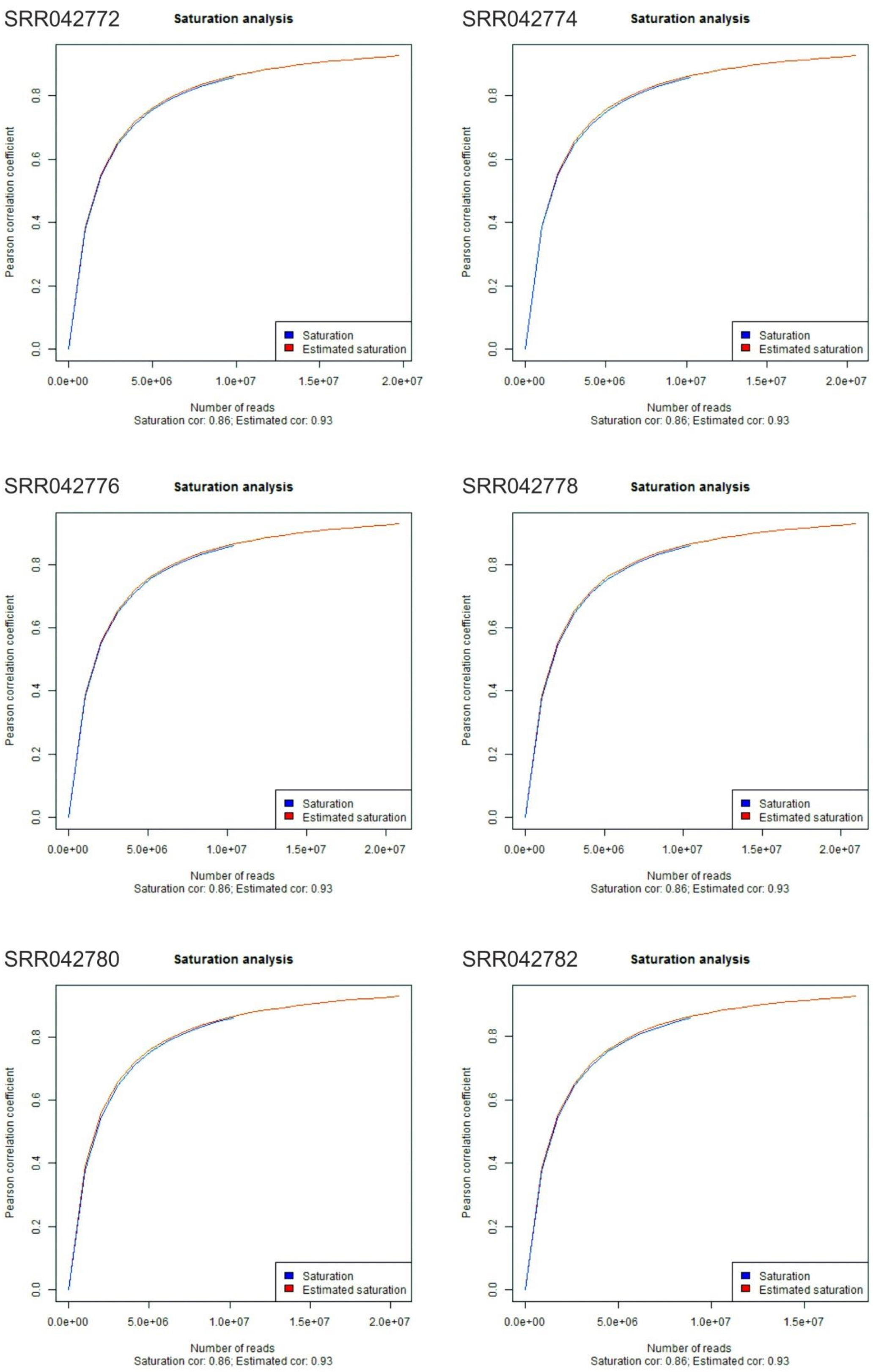

Fig C (a): Saturation plots of control Schwann cells' samples.

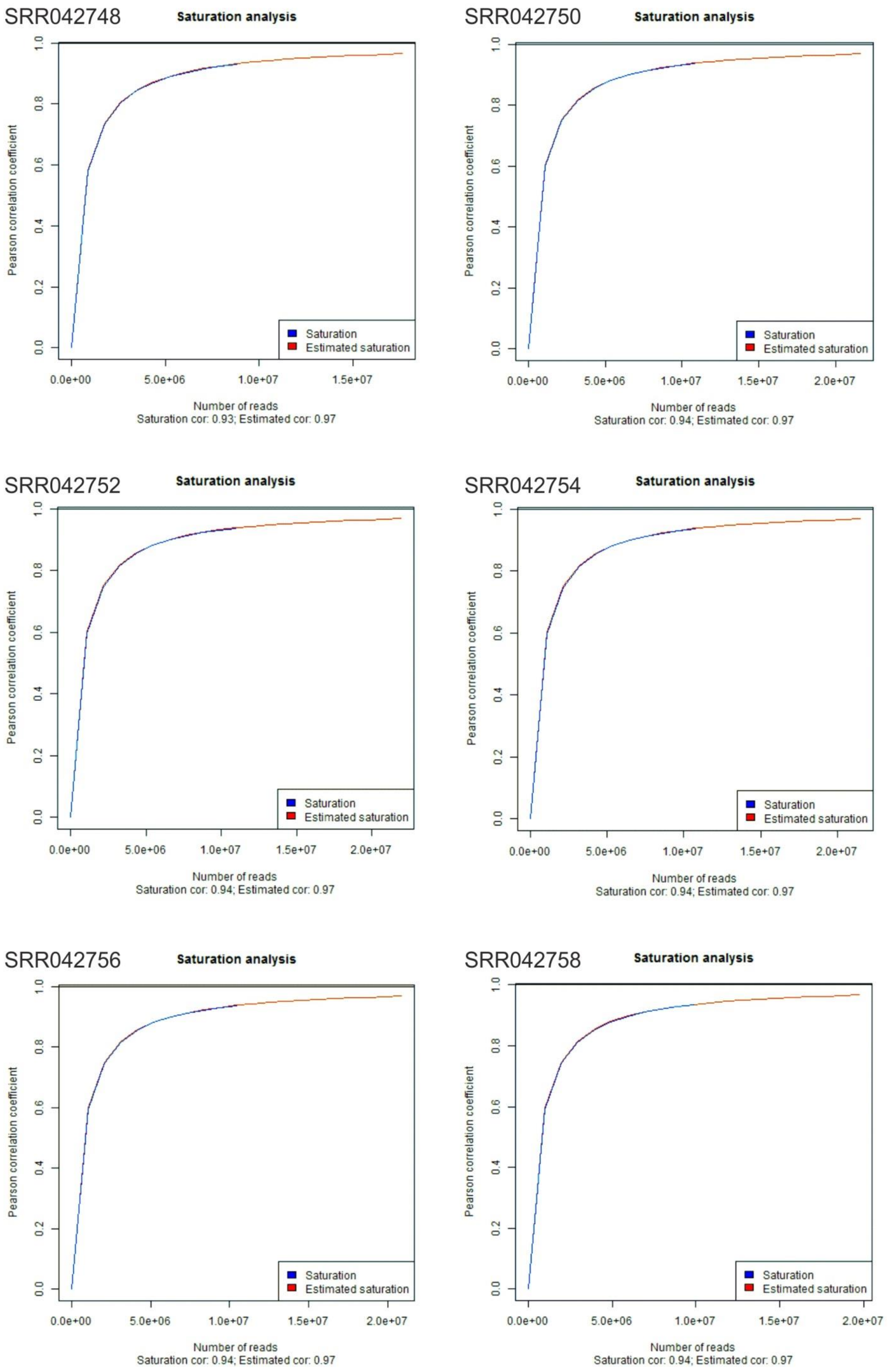

Fig C (b): Saturation plots of MPNST samples.

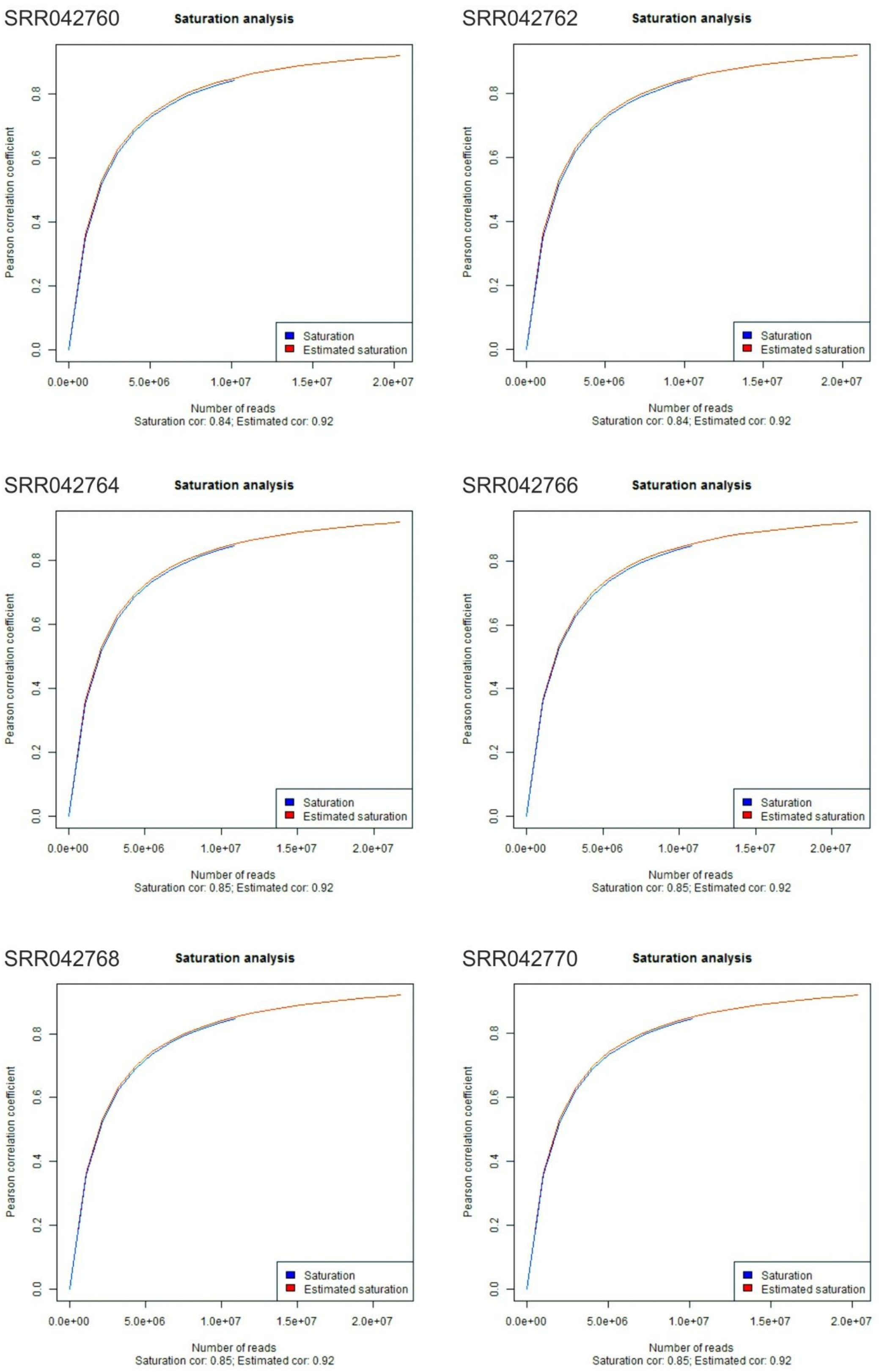

Fig C (c): Saturation plots of NF samples.

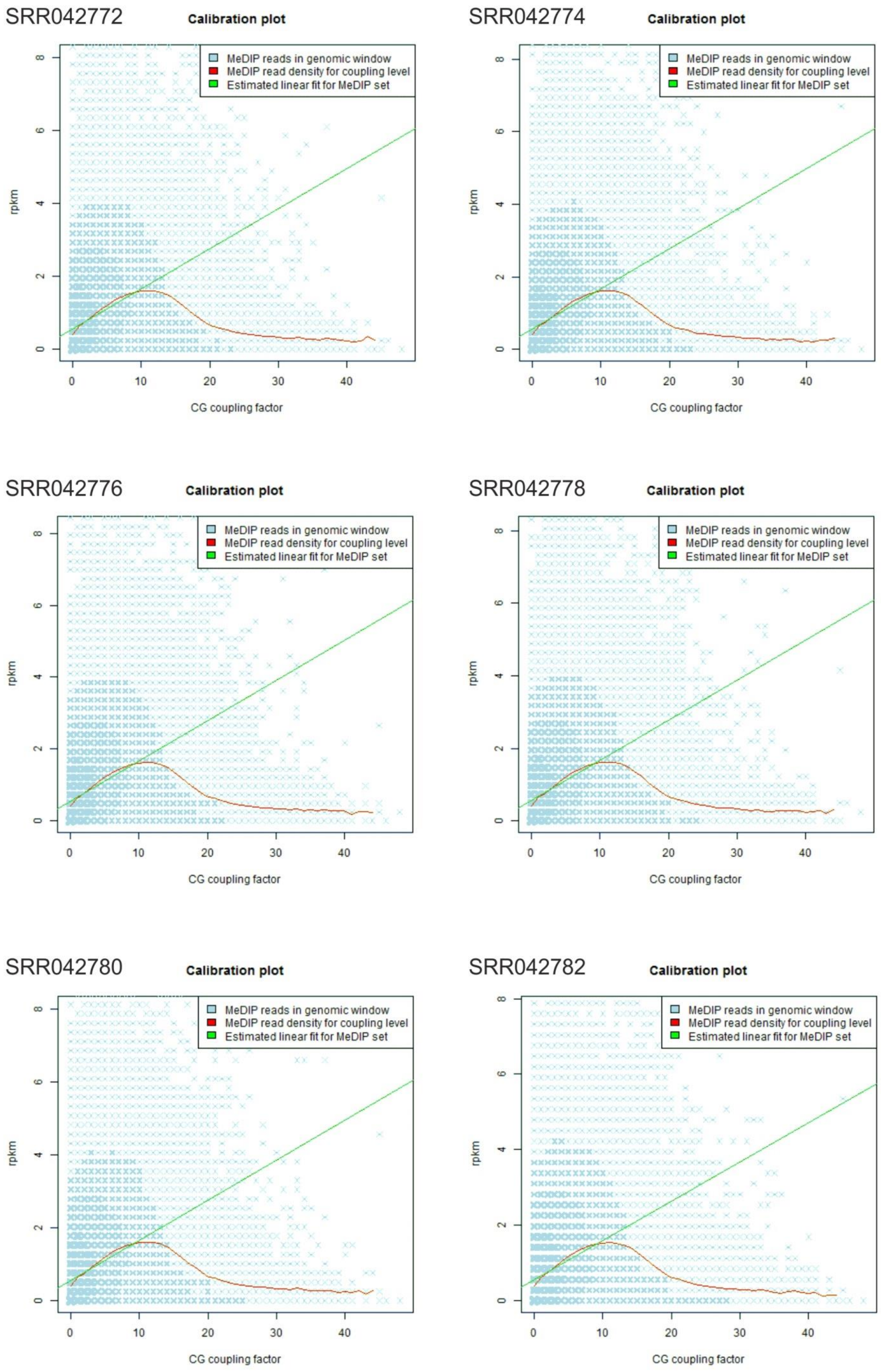

Fig D (a): Calibration plots of control Schwann cells' samples.

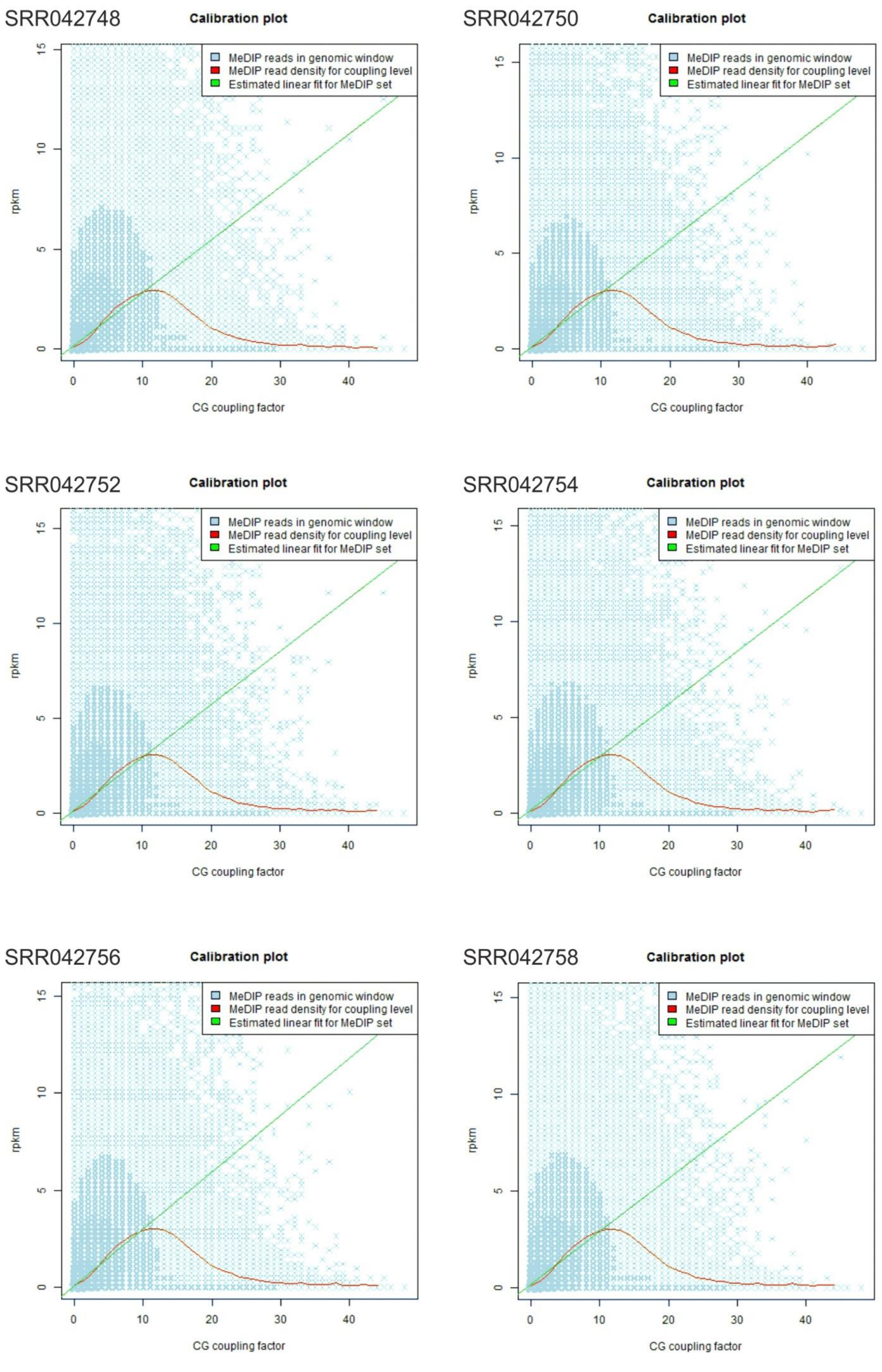

Fig D (b): Calibration plots of control Schwann cells' samples.

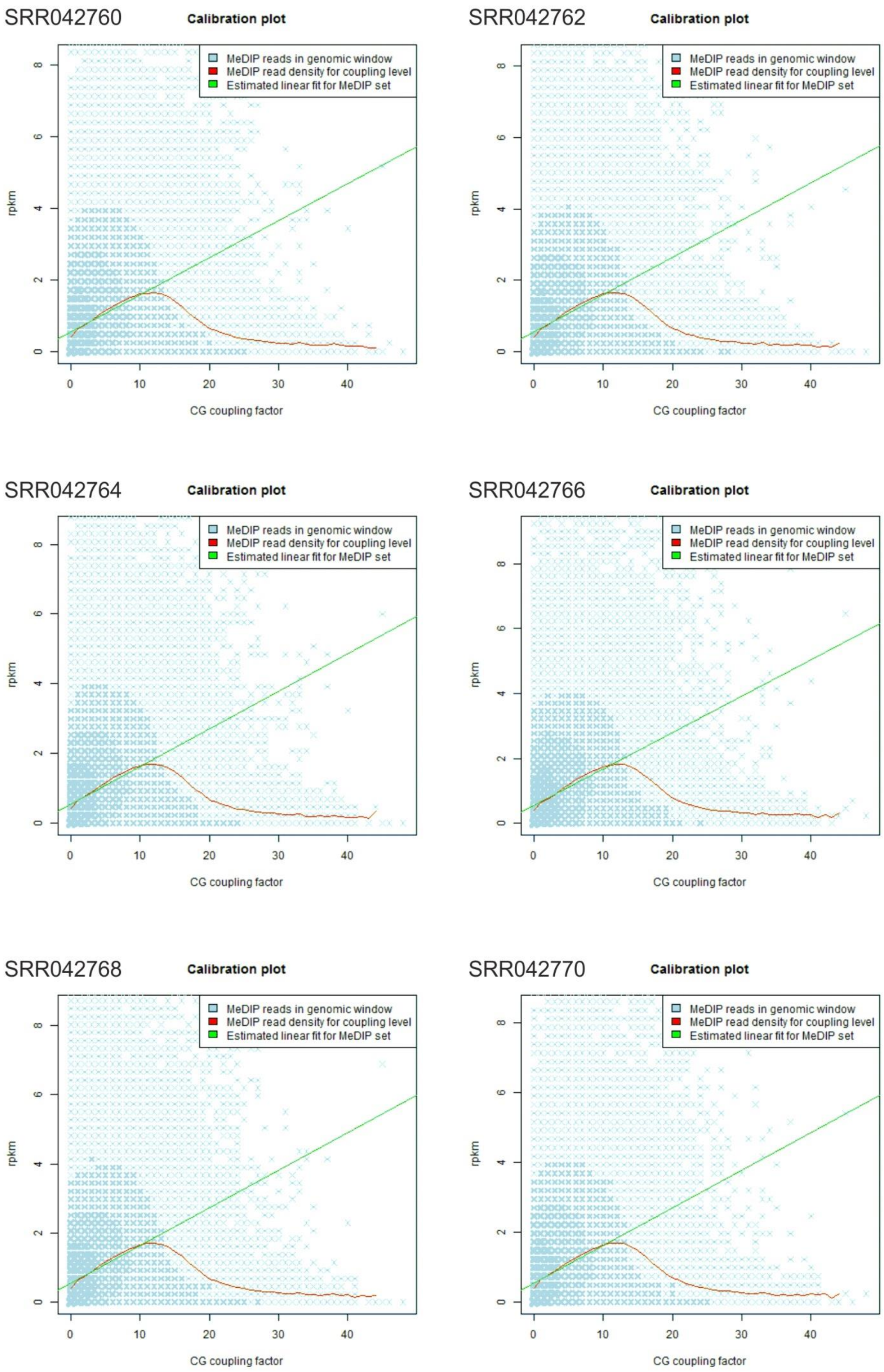

Fig D (c): Calibration plots of NF samples.

References

1. Hsieh Y-Y, Chou C-J, Lo H-L, Yang P-M. Repositioning of a cyclin-dependent kinase inhibitor GW8510 as a ribonucleotide reductase M2 inhibitor to treat human colorectal cancer. Cell Death Discov. 2016;2: 16027. doi:10.1038/cddiscovery.2016.27
2. Lecomte T, Ferraz J-M, Zinzindohoué F, Loriot M-A, Tregouet D-A, Landi B, et al. Thymidylate synthase gene polymorphism predicts toxicity in colorectal cancer patients receiving 5-fluorouracil-based chemotherapy. Clin Cancer Res. 2004;10: 5880–8. doi:10.1158/1078-0432.CCR-04-0169
3. Yang J, Du X. Genomic and molecular aberrations in malignant peripheral nerve sheath tumor and their roles in personalized target therapy. Surg Oncol. 2013;22: e53–e57. doi:10.1016/j.suronc.2013.06.003
4. Upadhyaya M, Spurlock G, Thomas L, Thomas NST, Richards M, Mautner VF, et al. Microarray-based copy number analysis of neurofibromatosis type-1 (NF1)-associated malignant peripheral nerve sheath tumors reveals a role for Rho-GTPase pathway genes in NF1 tumorigenesis. Hum Mutat. 2012;33: 763–776. doi:10.1002/humu.22044
5. Schmidt H, Taubert H, Meye A, Würl P, Bache M, Bartel F, et al. Gains in chromosomes 7, 8q, 15q and 17q are characteristic changes in malignant but not in benign peripheral nerve sheath tumors from patients with Recklinghausen’s disease. Cancer Let. 2000. doi:10.1016/S0304-3835(00)00426-2
6. Kolberg M, Høland M, Lind GE, Ågesen TH, Skotheim RI, Sundby Hall K, et al. Protein expression of BIRC5, TK1, and TOP2A in malignant peripheral nerve sheath tumours - A prognostic test after surgical resection. Mol Oncol. 2015;9: 1129–1139. doi:10.1016/j.molonc.2015.02.005
7. Kresse SH, Skårn M, Ohnstad HO, Namløs HM, Bjerkehagen B, Myklebost O, et al. DNA copy number changes in high-grade malignant peripheral nerve sheath tumors by array CGH. Mol Cancer. 2008;7: 48. doi:10.1186/1476-4598-7-48
8. Legius E, Dierick H, Wu R, Hall BK, Marynen P, Cassiman JJ, et al. TP53 mutations are frequent in malignant NF1 tumors. Genes Chromosom Cancer. 1994;10: 250–5.
9. Lothe RA, Smith-Sørensen B, Hektoen M, Stenwig AE, Mandahl N, Sæter G, et al. Biallelic inactivation ofTP53 rarely contributes to the development of malignant peripheral nerve sheath tumors. Genes Chromosom Cancer. 2001;30: 202–206. doi:10.1002/1098-2264(2000)9999:9999<::AID-GCC1079>3.0.CO;2-5

10. Sabah M, Cummins R, Leader M, Kay E. Loss of p16INK4A Expression Is Associated With Allelic Imbalance/Loss of Heterozygosity of Chromosome 9p21 in Microdissected Malignant Peripheral Nerve Sheath Tumors. Appl Immunohistochem Mol Morphol. 2006;14: 97–102. doi:10.1097/01.pai.0000143787.80564.f5
11. De Raedt T, Beert E, Pasmant E, Luscan A, Brems H, Ortonne N, et al. PRC2 loss amplifies Ras-driven transcription and confers sensitivity to BRD4-based therapies. Nature. 2014;514: 247–251. doi:10.1038/nature13561
12. Holtkamp N, Malzer E, Zietsch J, Okuducu AF, Mucha J, Mawrin C, et al. EGFR and erbB2 in malignant peripheral nerve sheath tumors and implications for targeted therapy. Neuro Oncol. 2008;10: 946–57. doi:10.1215/15228517-2008-053
13. Rahrmann EP, Moriarity BS, Otto GM, Watson AL, Choi K, Collins MH, et al. Trp53 Haploinsufficiency Modifies EGFR-Driven Peripheral Nerve Sheath Tumorigenesis. Am J Pathol. 2014;184: 2082–2098. doi:10.1016/j.ajpath.2014.04.006
14. Tabone-Eglinger S, Bahleda R, Côté J-F, Terrier P, Vidaud D, Cayre A, et al. Frequent EGFR Positivity and Overexpression in High-Grade Areas of Human MPNSTs. Sarcoma. 2008;2008: 849156. doi:10.1155/2008/849156
15. Thomas LE, Winston J, Rad E, Mort M, Dodd KM, Tee AR, et al. Evaluation of copy number variation and gene expression in neurofibromatosis type-1-associated malignant peripheral nerve sheath tumours. Hum Genomics. 2015;9: 3. doi:10.1186/s40246-015-0025-3
16. Sun D, Haddad R, Kraniak JM, Horne SD, Tainsky MA. RAS/MEK-independent gene expression reveals BMP2-related malignant phenotypes in the Nf1-deficient MPNST. Mol Cancer Res. 2013;11: 616–27. doi:10.1158/1541-7786.MCR-12-0593
17. Cheah AL, Billings SD, Goldblum JR, Carver P, Tanas MZ, Rubin BP. STAT6 rabbit monoclonal antibody is a robust diagnostic tool for the distinction of solitary fibrous tumour from its mimics. Pathology. 2014;46: 389–95. doi:10.1097/PAT.0000000000000122
18. Sorci G, Giovannini G, Riuzzi F, Bonifazi P, Zelante T, Zagarella S, et al. The Danger Signal S100B Integrates Pathogen– and Danger–Sensing Pathways to Restrain Inflammation. PLoS Pathog. Public Library of Science; 2011;7: e1001315. doi:10.1371/JOURNAL.PPAT.1001315
19. Donato R, Sorci G, Riuzzi F, Arcuri C, Bianchi R, Brozzi F, et al. S100B's double life: Intracellular regulator and extracellular signal. Biochim Biophys Acta.

2009;1793: 1008–1022. doi:10.1016/j.bbamcr.2008.11.009
20. Feber a, Wilson G, Zhang L, Presneau N, Idowu B, Down T, et al. Comparative methylome analysis of benign and malignant peripheral nerve sheath tumors. 2011; 515–524. doi:10.1101/gr.109678.110.
21. Xie C, Han Y, Liu Y, Han L, Liu J. miRNA-124 down-regulates SOX8 expression and suppresses cell proliferation in non-small cell lung cancer. Int J Clin Exp Pathol. 2014;7: 6534–42.
22. Diggs-Andrews KA, Brown JA, Gianino SM, Rubin JB, Wozniak DF, Gutmann DH. Sex Is a major determinant of neuronal dysfunction in neurofibromatosis type 1. Ann Neurol. 2014;75: 309–316. doi:10.1002/ana.24093
23. Rowley PT, Kosciolek B, Bader JL. Oncogene expression in neurofibromatosis. Ann N Y Acad Sci. 1986;486: 327–32.
24. Miller SJ, Rangwala F, Williams J, Ackerman P, Kong S, Jegga AG, et al. Large-scale molecular comparison of human Schwann cells to malignant peripheral nerve sheath tumor cell lines and tissues. Cancer Res. 2006;66: 2584–2591. doi:10.1158/0008-5472.CAN-05-3330
25. Hoshi N, Yamaki T, Hiraki H, Natsume T, Saitoh A, Watanabe K, et al. Functional nerve growth factor receptor in von Recklinghausen neurofibromatosis: an immunocytochemical and short-term culture study. Pathol Int. 1996;46: 1–8.
26. Dechant G, Barde Y-A. The neurotrophin receptor p75NTR: novel functions and implications for diseases of the nervous system. Nat Neurosci. 2002;5: 1131. doi:10.1038/NN1102-1131
27. Roux PP, Barker PA. Neurotrophin signaling through the p75 neurotrophin receptor. Prog Neurobiol. 2002;67: 203–233. doi:10.1016/S0301-0082(02)00016-3
28. Underwood CK, Coulson EJ. The p75 neurotrophin receptor. Int J Biochem Cell Biol. 2008;40: 1664–1668. doi:10.1016/j.biocel.2007.06.010
29. Vicario A, Kisiswa L, Tann JY, Kelly CE, Ibáñez CF, Beg A, et al. Neuron-type-specific signaling by the p75NTR death receptor is regulated by differential proteolytic cleavage. J Cell Sci. 2015;128: 1507–17. doi:10.1242/jcs.161745
30. Yang Z, Chen H, Huo L, Yang Z, Bai Y, Fan X, et al. Epigenetic inactivation and tumor-suppressor behavior of NGFR in human colorectal cancer. Mol Cancer Res. 2015;13: 107–19. doi:10.1158/1541-7786.MCR-13-0247
31. Patel A V, Eaves D, Jessen WJ, Rizvi TA, Ecsedy JA, Qian MG, et al. Ras-driven transcriptome analysis identifies aurora kinase A as a potential malignant peripheral nerve sheath tumor therapeutic target. Clin Cancer Res. 2012;18:

5020–30. doi:10.1158/1078-0432.CCR-12-1072

32. Weng Y-R, Cui Y, Fang J-Y. Biological functions of cytokeratin 18 in cancer. Mol Cancer Res. 2012;10: 485–93. doi:10.1158/1541-7786.MCR-11-0222

33. Vidal AC, Henry NM, Murphy SK, Oneko O, Nye M, Bartlett JA, et al. PEG1/MEST and IGF2 DNA methylation in CIN and in cervical cancer. ClinTransl Oncol. 2014;16: 266–72. doi:10.1007/s12094-013-1067-4

34. Huff V. Wilms' tumours: about tumour suppressor genes, an oncogene and a chameleon gene. Nat Rev Cancer. 2011;11: 111–21. doi:10.1038/nrc3002

35. Mžik M, Chmelařová M, John S, Laco J, Slabý O, Kiss I, et al. Aberrant methylation of tumour suppressor genes WT1, GATA5 and PAX5 in hepatocellular carcinoma. Clin Chem Lab Med. 2016;54: 1971–1980. doi:10.1515/cclm-2015-1198

36. Meseure D, Vacher S, Alsibai KD, Nicolas A, Chemlali W, Caly M, et al. Expression of ANRIL-Polycomb Complexes-CDKN2A/B/ARF Genes in Breast Tumors: Identification of a Two-Gene (EZH2/CBX7) Signature with Independent Prognostic Value. Mol Cancer Res. 2016;14: 623–33. doi:10.1158/1541-7786.MCR-15-0418

37. Cao Q, Mani R-S, Ateeq B, Dhanasekaran SM, Asangani IA, Prensner JR, et al. Coordinated regulation of polycomb group complexes through microRNAs in cancer. Cancer Cell. NIH Public Access; 2011;20: 187–99. doi:10.1016/j.ccr.2011.06.016

38. Pallante P, Sepe R, Puca F, Fusco A. High mobility group A proteins as tumor markers. Front Med. 2015;2: 15. doi:10.3389/fmed.2015.00015

39. Forzati F, Federico A, Pallante P, Abbate A, Esposito F, Malapelle U, et al. CBX7 is a tumor suppressor in mice and humans. J Clin Invest. 2012;122: 612–23. doi:10.1172/JCI58620

40. Federico A, Pallante P, Bianco M, Ferraro A, Esposito F, Monti M, et al. Chromobox protein homologue 7 protein, with decreased expression in human carcinomas, positively regulates E-cadherin expression by interacting with the histone deacetylase 2 protein. Cancer Res. American Association for Cancer Research; 2009;69: 7079–87. doi:10.1158/0008-5472.CAN-09-1542

41. Sepe R, Formisano U, Federico A, Forzati F, Bastos AU, D'Angelo D, et al. CBX7 and HMGA1b proteins act in opposite way on the regulation of the SPP1 gene expression. Oncotarget. 2015;6: 2680–92. doi:10.18632/oncotarget.2777

42. Pallante P, Sepe R, Federico A, Forzati F, Bianco M, Fusco A. CBX7 modulates the expression of genes critical for cancer progression. PLoS One. 2014;9: e98295. doi:10.1371/journal.pone.0098295

43. Cao Q, Yu J, Dhanasekaran SM, Kim JH, Mani R-S, Tomlins SA, et al. Repression of E-cadherin by the polycomb group protein EZH2 in cancer. Oncogene. 2008;27: 7274–84. doi:10.1038/onc.2008.333

44. Tan J, Yan Y, Wang X, Jiang Y, Xu HE. EZH2: biology, disease, and structure-based drug discovery. Acta Pharmacol Sin. 2014;35: 161–74. doi:10.1038/aps.2013.161

45. Wassef M, Michaud A, Margueron R. Association between EZH2 expression, silencing of tumor suppressors and disease outcome in solid tumors. Cell Cycle. 2016;15: 2256–62. doi:10.1080/15384101.2016.1208872

46. Zhang P, Yang X, Ma X, Ingram DR, Lazar AJ, Torres KE, et al. Antitumor effects of pharmacological EZH2 inhibition on malignant peripheral nerve sheath tumor through the miR-30a and KPNB1 pathway. Mol Cancer. 2015;14: 55. doi:10.1186/s12943-015-0325-1

47. Long P, Stradecki H, Minturn J. Differential aminoacylase expression in neuroblastoma. Int J Cancer. 2011;129: 1322–30.

48. Yan W, Shih J. Identification of unique expression signatures and therapeutic targets in esophageal squamous cell carcinoma. BMC Res Notes. 2012;5: 73.

49. Long PM, Moffett JR, Namboodiri AMA, Viapiano MS, Lawler SE, Jaworski DM. N-Acetylaspartate (NAA) and N-Acetylaspartylglutamate (NAAG) Promote Growth and Inhibit Differentiation of Glioma Stem-like Cells. J Biol Chem. 2013;288: 26188–26200. doi:10.1074/jbc.M113.487553

50. Long PM, Tighe SW, Driscoll HE, Fortner KA, Viapiano MS, Jaworski DM. Acetate Supplementation as a Means of Inducing Glioblastoma Stem-Like Cell Growth Arrest. J Cell Physiol. 2015;230: 1929–1943. doi:10.1002/jcp.24927

51. Tsen AR, Long PM, Driscoll HE, Davies MT, Teasdale BA, Penar PL, et al. Triacetin-based acetate supplementation as a chemotherapeutic adjuvant therapy in glioma. Int J Cancer. 2014;134: 1300–1310. doi:10.1002/ijc.28465

52. Long P, Moffett J, Namboodiri A. N-acetylaspartate (NAA) and N-acetylaspartylglutamate (NAAG) promote growth and inhibit differentiation of glioma stem-like cells. J Biol Chem. 2013;288: 26188–26200.

53. Tucker SL, Gharpure K, Herbrich SM, Unruh AK, Nick AM, Crane EK, et al. Molecular biomarkers of residual disease after surgical debulking of high-grade serous ovarian cancer. Clin Cancer Res. 2014;20: 3280–8. doi:10.1158/1078-0432.CCR-14-0445

54. Ye H, Yu T, Temam S, Ziober BL, Wang J, Schwartz JL, et al. Transcriptomic dissection of tongue squamous cell carcinoma. BMC Genomics. 2008;9: 69. doi:10.1186/1471-2164-9-69

55. Kopantzev EP, Monastyrskaya GS, Vinogradova T V., Zinovyeva M V., Kostina MB, Filyukova OB, et al. Differences in gene expression levels between early and later stages of human lung development are opposite to those between normal lung tissue and non-small lung cell carcinoma. Lung Cancer. 2008;62: 23–34. doi:10.1016/j.lungcan.2008.02.011
56. Gautier L, Cope L, Bolstad BM, Irizarry RA. affy--analysis of Affymetrix GeneChip data at the probe level. Bioinformatics. 2004;20: 307–315. doi:10.1093/bioinformatics/btg405
57. Ritchie ME, Phipson B, Wu D, Hu Y, Law CW, Shi W, et al. limma powers differential expression analyses for RNA-sequencing and microarray studies. Nucleic Acids Res. 2015;43: e47. doi:10.1093/nar/gkv007
58. Sun YA. ABarray: Microarray QA and statistical data analysis for Applied Biosystems Genome Survey Microrarray (AB1700) gene expression data. 2006.
59. Leek JT, Johnson WE, Parker HS, Jaffe AE, Storey JD. The sva package for removing batch effects and other unwanted variation in high-throughput experiments. Bioinformatics. 2012;28: 882–3. doi:10.1093/bioinformatics/bts034
60. Gentleman, R., Carey, V., Huber, W., Hahne F. genefilter: genefilter: methods for filtering genes from high-throughput experiments. Available: https://www.bioconductor.org/packages/devel/bioc/manuals/genefilter/man/genefilter.pdf
61. Durinck S, Spellman PT, Birney E, Huber W. Mapping identifiers for the integration of genomic datasets with the R/Bioconductor package biomaRt. Nat Protoc. 2009;4: 1184–91. doi:10.1038/nprot.2009.97
62. Rasche A, Al-Hasani H, Herwig R. Meta-analysis approach identifies candidate genes and associated molecular networks for type-2 Diabetes mellitus. BMC Genomics. 2008;9: 310. doi:10.1186/1471-2164-9-310
63. Li H, Durbin R. Fast and accurate long-read alignment with Burrows-Wheeler transform. Bioinformatics. 2010;26: 589–95. doi:10.1093/bioinformatics/btp698
64. Li H, Handsaker B, Wysoker A, Fennell T, Ruan J, Homer N, et al. The Sequence Alignment/Map format and SAMtools. Bioinformatics. 2009;25: 2078–9. doi:10.1093/bioinformatics/btp352
65. Thorvaldsdottir H, Robinson JT, Mesirov JP. Integrative Genomics Viewer (IGV): high-performance genomics data visualization and exploration. Br Bioinform. 2013;14: 178–192. doi:10.1093/bib/bbs017
66. Lienhard M, Grimm C, Morkel M, Herwig R, Chavez L. MEDIPS: Genome-wide differential coverage analysis of sequencing data derived from DNA enrichment experiments. Bioinformatics. 2014;30: 284–286.

doi:10.1093/bioinformatics/btt650
