# Supplementary material for: Gene signature associated with benign neurofibroma transformation to malignant peripheral nerve sheath tumors
Source: PLoS One. 2017 May 24;12(5):e0178316. doi: 10.1371/journal.pone.0178316 (PMC5443557; doi:10.1371/journal.pone.0178316)
Supplement: S2 Appendix — (PDF) [file pone.0178316.s022.pdf]

PRISMA 2009 Checklist

Section/topic	#	Checklist item	Reported on page #
**TITLE**			
Title	1	Meta-analysis to define the gene signature associated with benign neurofibroma transformation to malignant peripheral nerve sheath tumors	
**ABSTRACT**			
Structured summary	2	As first step to generate biomarkers in the evolution to malignity of neurofibromatosis disease and to develop therapeutic measures to reverse the malignant phenotype, we integrated five gene expression studies (four from human and one from mouse) by using a meta-analysis method. These studies were found in GEO and ArrayExpress databases using the key words NF1, MPNST, neurofibromatosis, and neurofibroma. Microarrays platforms Affymetrix, Agilent, ABI and Illumina were allowed for eligibility, and only studies accepted for publication and including neurofibroma and MPNST samples were considered. The method used to integrate data was a slight modification of a score-based method previously described for meta-analysis of heterogeneous data. As a result, we obtained a gene signature of 579 genes from which we classified the genes with the highest absolute scores as promising biomarkers of the evolution from benign neurofibromas to malignant tumors. As main application of the gene signature, we used it to query drug expression databases and seek therapeutic measures that could reverse the malignant phenotype. Cantharidin, tamoxifen and trichostatin A and other histone deacetylase inhibitors were identified, corroborating previous experimental approaches, as well as the reliability of our average signature.	2
**INTRODUCTION**			
Rationale	3	Despite previous individual efforts in the field of gene expression, we still lack of robust prognostic biomarkers to predict the evolution of benign neurofibromas to malignant peripheral nerve sheath tumors and to adopt early therapeutic measures in the neurofibromatosis disease.	3
Objectives	4	As first step to identify potential robust biomarkers, our aim is to integrate all those individual efforts in an average gene signature associated to the malignant transformation.	5
**METHODS**			
Protocol and registration	5	We have slightly modified the score-based meta-analysis method previously used for type-2 Diabetes mellitus and Down syndrome [1,2] to obtain more robust gene scores and to use it with expression data derived from different size platforms.	3
Eligibility criteria	6	Studies from GEO and ArrayExpress databases were eligible. These studies should contain high-throughput sequencing or microarray data from platforms Affymetrix, Agilent, ABI or Illumina, and they should have been accepted for publication and contain neurofibroma and MPNST samples.	29
Information sources	7	GEO (NCBI) and ArrayExpress (EBI) public databases.	29
Search	8	We used key words NF1, MPNST, neurofibromatosis, and neurofibroma to select studies that included high-throughput sequencing and microarray in which samples of MPNST and neurofibroma were compared. No time limits were imposed.	29

Study selection	9	The eligible studies were included in the meta-analysis.	
Data collection process	10	Each study data were extracted independently.	
Data items	11	Benign (dermal/plexiform) neurofibroma (NF); and b) Malignant peripheral nerve sheath tumors (MPNST).<br>Studies E-MEXP-353 [3], E-TABM-69 [4], GSE41747 (human) [5], GSE66743 [6], GSE41747 (mouse) [5].	6; Table 1
Risk of bias in individual studies	12	Only the expression set of features with ANOVA FDR <0.05 were included in the meta-analysis. Independent principal component analysis (PCA) was then used for assessing the appropriate separation of phenotype samples, in order to avoid sample bias in each individual study.	29
Summary measures	13	For each gene in each study, the next measures were obtained directly from limma R package: logFC, pval, adj.pval and B. In addition, we determined for each gene: logFC_m (median of expression ratios between phenotype samples) and score s (product of factors S(logFC_m), FII, and (1- pval)). The size effect measure logFC_m was normalized in the interval [-1, 0] for down-regulated genes and [0, 1] for up-regulated ones in order to avoid bias among studies and to have consistent score values for each gene. S(logFC_m) indicates the scaled logFC_m (normalized between -1 and +1). FII (second factor included in the formula to compute the scores); this factor decreases as the ratio MAD/absolute(logFC_m) increases. MAD indicates the median absolute deviation of each ratio between two phenotypic samples to the logFC_m).	30, 31
Synthesis of results	14	For each gene we calculated the final values of logFC and logFC_m, as the median of these values in each individual study, and the final score as the addition of scores from individual studies. Values of logFC, logFC_m and scores prioritized human values when discrepancies between human and mouse data were observed. To determine the gene signature, a threshold for score (10% of highest absolute values) and logFC > 0.99 was imposed to the list of scored genes.	30, 31

Section/topic	#	Checklist item	Reported on page #
Risk of bias across studies	15	As additional attributes for each gene we included three values to assess the bias across studies: the number of studies that included probes representing the particular gene in the respective platform, the inclusion or exclusion of mouse data to compute the score and the median values of logFC and logFC_m, and the Bhattacharyya distance ratio (BD_ratio). The BD_ratio in the interval [0, 100] was computed as reference of the homogeneous contribution of each study in the score computation (the lower the value, the more homogeneous contribution). This value normalizes the BD according to the number of studies included in the computation of that gene score. Exceptionally, BD_ratio equal to zero was assigned to genes for which only one study was available.	8, 31
Additional analyses	16	Use of the gene signature to query drug expression databases (CMap and DrugMatrix) by using NFFinder to search for drugs showing the opposite expression pattern of the gene signature.	9, 32
**RESULTS**			
Study selection	17	The five eligible studies were selected for the analysis.	

Study characteristics	18	E-MEXP-353 [3], E-TABM-69 [4], GSE41747 (human) [5], GSE66743 [6], GSE41747 (mouse) [5]	6; Table 1
Risk of bias within studies	19	PCA plots show appropriate samples separation in each experiment.	S4 and S5 Figs.
Results of individual studies	20	A table that contains the logFC, logFC_m and score values for each individual experiment is shown. This table derives from joining data from each study tables similar to S13 Table, that shows as an example the head and tail of this table for the study GSE66743 (these tables are not included).	S1 Table
Synthesis of results	21	We have obtained a signature of 579 unique ENSEMBL human genes with non-null score and absolute median logFC value >0.99. Showing positive and negative score, respectively, 336 and 243 genes were up- and downregulated, respectively. This gene signature is embedded in a larger list shown in S2 Table (A) from which the gene signature was filtered. Table 2 shows the 20 genes with the highest and lowest score values that will be discussed as promising biomarkers. The functional characterization and chromosomal location of signature genes is also described.	S2 Table (A);<br>8, 9; Table 2
Risk of bias across studies	22	The three measures of risk of bias across studies are shown in S2 Table (A), Table 2 and S1 Appendix (Results. A):<br><br>Concerning the number of studies considered for each signature gene, the highest amount of genes depended on 5 studies (56.31%), 4 from human and one from mouse. 26.94%, 10.88%, 5% and 0.86% of genes depended on 4, 3, 2 and 1 study, respectively. As expected from the addition of five individual scores, the highest absolute average scores were obtained for genes represented in a higher number of studies (S1 Appendix (Results. A, S6 Fig (d))).<br><br>Mice scores were not included in the final computation of gene scores for 114 genes (~20% of signature genes), due to the absence of those genes in the mouse study, or because mouse and human studies showed different behavior, thus avoiding score bias due to mouse data. Most of genes ignoring mouse data were in the interval [-1, 1] of score values, which indicates that genes with the highest scores include mouse data in the computation of score values (S1 Appendix (Results. A, S6 Fig (a))).<br><br>Concerning BD-ratios, 530 genes (91.54% of the total gene signature) showed BD-ratios between 0 and 100. These genes showed higher absolute scores compared with genes with BD-ratios 0 or 100, as the distribution of genes indicates when we compare BD-ratio and score values (S1 Appendix (Results. A, S6 Fig (c))). The 8.46% of the total gene signature (BD_ratios 0 or 100) shows the highest bias because gene score values depend on a single study (the gene was represented by probes that were in only one platform or, although the gene was represented by more than one platform, there was only one in which the gene score was not-null).	S1 Appendix
Additional analysis	23	The therapeutic drugs mainly retrieved by NFFinder to reverse the malignant phenotype were trichostatin A and other histone deacetylase inhibitors. Among other compounds, cantharidin and tamoxifen were also retrieved.	9, 10; S3 Table (A)
**DISCUSSION**			
Summary of evidence	24	The score-ranked gene signature associated to the malignant transformation of neurofibromas includes many genes that were identified in the individual studies and, in some cases, linked to patients' outcome. In general, our results agreed with reported expression data that compare malignant peripheral nerve sheath tumors with benign neurofibromas. The slight differences observed are mainly due to our strict score threshold and to the type of tissue involved in the comparison. In addition, we used this gene signature to find in-silico potential therapeutic measures	20, 21, 22-26

		able to reverse the malignant phenotype. We thus interrogate drug expression databases by using NFFinder. trichostatin A and other deacetylase inhibitors were mainly retrieved. These inhibitor compounds were previously tested in vitro and in tumor xenografts [7] and, due to their strong effect on malignant cells, they were suggested to be include in clinical trials. Cantharidin [8] and tamoxifen [9] were also previously tested for effectiveness to treat MPNST. These experimental evidences agreed with our therapeutic drug in-silico prediction and, moreover, it corroborates the reliability of our gene signature as representative of neurofibroma malignant evolution.	
Limitations	25	Main limitations of the meta-analysis derived from the heterogeneous nature of the starting data. We used expression human and disease murine models data obtained from different size array platforms. Genes could be represented in some of these platforms or even in only one. Although the score addition method rewards genes represented in all the platforms, it also allows include in the gene signature relevant genes that are only represented in some of the platforms. Inconsistencies and the biggest bias due to the inclusion of mouse data were avoided by prioritizing human data when there were discrepancies between human and mice data. Although a certain bias due to the inclusion of mouse data may still affect gene score values, we decided to include these data in order to obtain robust candidates for preclinical studies.	26, 27
Conclusions	26	This average gene signature, based on experimental evidences and representative of neurofibroma malignant evolution can thus be used to generate working hypotheses to test the highest absolute score genes as biomarkers of the malignant evolution or of the effectiveness of the treatment, as well as to search for other therapeutic recommendations. The meta-analysis method used to integrate sparse heterogeneous data could thus be generalized to other comparisons in the neurofibromatosis disease context and also in other rare diseases.	22-27
**FUNDING**			
Funding	27	Children's Tumor Foundation [2013–04–005]	

1. Rasche A, Al-Hasani H, Herwig R, Stumvoll M, Goldstein B, Haeften T, et al. Meta-Analysis Approach identifies Candidate Genes and associated Molecular Networks for Type-2 Diabetes Mellitus. BMC Genomics. 2008;9: 310. doi:10.1186/1471-2164-9-310
2. Vilardell M, Rasche A, Thormann A, Maschke-Dutz E, Pérez-Jurado LA, Lehrach H, et al. Meta-analysis of heterogeneous Down Syndrome data reveals consistent genome-wide dosage effects related to neurological processes. BMC Genomics. 2011;12: 229. doi:10.1186/1471-2164-12-229
3. Henderson SR, Guiliano D, Presneau N, McLean S, Frow R, Vujovic S, et al. A molecular map of mesenchymal tumors. Genome Biol. 2005;6: R76. doi:10.1186/gb-2005-6-9-r76
4. Lévy P, Ripoche H, Laurendeau I, Lazar V, Ortonne N, Parfait B, et al. Microarray-based identification of Tenascin C and Tenascin XB, genes possibly involved in tumorigenesis associated with neurofibromatosis type 1. Clin Cancer Res. 2007;13: 398–407. doi:10.1158/1078-0432.CCR-06-0182
5. Jessen WJ, Miller SJ, Jousma E, Wu J, Rizvi T a, Brundage ME, et al. MEK inhibition exhibits efficacy in human and mouse neurofibromatosis tumors. J Clin Invest. 2013;123: 340–347. doi:10.1172/JCI60578DS1
6. Kolberg M, Høland M, Lind GE, Ågesen TH, Skotheim RI, Sundby Hall K, et al. Protein expression of BIRC5, TK1, and TOP2A in malignant peripheral nerve sheath tumours - A prognostic test after surgical resection. Mol Oncol. 2015;9: 1129–1139. doi:10.1016/j.molonc.2015.02.005
7. Lopez G, Torres K, Liu J, Hernandez B, Young E, Belousov R, et al. Autophagic survival in resistance to histone deacetylase inhibitors:

novel strategies to treat malignant peripheral nerve sheath tumors. Cancer Res. NIH Public Access; 2011;71: 185–96. doi:10.1158/0008-5472.CAN-10-2799
8. Semenova G, Stepanova D, Deyev SM, Chernoff J. Medium throughput biochemical compound screening identifies novel agents for pharmacotherapy of neurofibromatosis type I. Biochimie. 2017;135: 1–5. doi:10.1016/j.biochi.2017.01.001
9. Byer SJ, Eckert JM, Brossier NM, Clodfelder-Miller BJ, Turk AN, Carroll AJ, et al. Tamoxifen inhibits malignant peripheral nerve sheath tumor growth in an estrogen receptor-independent manner. Neuro Oncol. 2011;13: 28–41. doi:10.1093/neuonc/noq146
